# Preventing evolutionary rescue in cancer using two-strike therapy

**DOI:** 10.1093/genetics/iyaf255

**Published:** 2025-11-26

**Authors:** Srishti Patil, Armaan Ahmed, Yannick Viossat, Robert Noble

**Affiliations:** Department of Mathematics, City St George’s, University of London, London EC1V 0HB, United Kingdom; Indian Institute of Science Education and Research, Pune 411008, India; Division of Theoretical Systems Biology, German Cancer Research Center (DKFZ), Heidelberg 69120, Germany; Department of Applied Mathematics & Statistics, Johns Hopkins University, Baltimore, MD 21218, United States; Department of Mathematics, Johns Hopkins University, Baltimore, MD 21218, United States; CEREMADE, Université Paris-Dauphine, Université PSL, CNRS, Paris 75016, France; Department of Mathematics, City St George’s, University of London, London EC1V 0HB, United Kingdom

**Keywords:** mathematical oncology, evolutionary therapy, evolutionary rescue, therapeutic resistance, cancer treatment, extinction therapy

## Abstract

First-line cancer treatment frequently fails due to initially rare therapeutic resistance. An important clinical question is then how to schedule subsequent treatments to maximize the probability of tumor eradication. Here, we provide a theoretical solution to this problem by using mathematical analysis and extensive stochastic simulations within the framework of evolutionary rescue theory to determine how best to exploit the vulnerability of small tumors to stochastic extinction. Whereas standard clinical practice is to wait for evidence of relapse, we confirm a recent hypothesis that the optimal time to switch to a second treatment is when the tumor is close to its minimum size before relapse, when it is likely undetectable. This optimum can lie slightly before or slightly after the nadir, depending on tumor parameters. Given that this exact time point may be difficult to determine in practice, we study windows of high extinction probability that lie around the optimal switching point, showing that switching after the relapse has begun is typically better than switching too early. We further reveal how treatment efficacy and tumor demographic and evolutionary parameters influence the predicted clinical outcome, and we determine how best to schedule drugs of unequal efficacy. Our work establishes a foundation for further experimental and clinical investigation of this evolutionarily-informed multi-strike treatment strategy.

## Teaser

Suppose a cancer treatment fails because a small fraction of tumour cells are resistant. Might we still be able to cure the patient by switching to another treatment at just the right time? Using mathematical analyses and simulations, Patil et al. found that the best time to switch treatments is much earlier than usual, before evidence of relapse, and the authors determined how doses and other parameters influence the cure probability. Their findings support clinical trials of a new, unconventional cancer treatment strategy.

## Introduction

Just as species in an ecosystem interact, compete for resources, adapt to changing environmental conditions and undergo natural selection, so cancer clones rise and fall in a tumor ecosystem. Darwinian principles inevitably determine therapeutic responses (Iwasa et al. 2006) including the emergence of resistance, which, despite pharmaceutical advances, remains the greatest challenge in oncology. As cancer cells can use a variety of adaptive strategies to achieve resistance (Pressley et al. 2021), targeting a single molecular mechanism often proves ineffective in the long term (Greaves and Maley 2012). Understanding intratumor evolutionary processes provides a rational foundation for developing treatment strategies that, by explicitly accounting for evolutionary dynamics, achieve better clinical outcomes (Aktipis et al. 2011; Korolev et al. 2014; Enriquez-Navas et al. 2015). Mathematical modeling of clonal dynamics and the emergence of resistance is critical for optimizing clinical treatment strategies based on evolutionary principles. Consequently, the historical development of evolutionary therapies has followed a trajectory that begins with a theoretical and mathematical exploration of associated eco-evolutionary models (West et al. 2023).

The clinical strategy we study here uses evolutionary rescue theory to inform the probability of tumor extinction under multiple treatment administrations or “strikes.” Although the term evolutionary rescue arose in a conservation context (Gomulkiewicz and Holt 1995), the same theory is applicable when extinction is the goal, such as in bacterial infections or cancer (Coldman and Goldie 1983; Alexander et al. 2014; Torres-Barceló et al. 2014). Since an oncologist can influence the tumor environment, they can anticipate the evolutionary trajectories of cancer clones and, in theory, follow a strategy to avoid evolutionary rescue and so cure the patient (Gatenby et al. 2019). The key idea is that, even if a single strike fails to eradicate cancer cells due to resistant phenotypes, it can still leave the population small and fragmented. The probability that a resistant phenotype in the population leads to evolutionary rescue decreases, rendering the population more vulnerable to stochastic extinction (Torres-Barceló et al. 2014). Moreover, a small population is less able to adapt to environmental changes owing to loss of genetic variation (Alexander et al. 2014). Cell proliferation may also slow due to Allee effects (Dennis et al. 2016) during tumor initiation due to processes like angiogenesis and growth factor signaling (Sewalt et al. 2016; Azimzade et al. 2021; Gerlee et al. 2022). Subsequent therapeutic strikes, if well timed, might be able to exploit these weaknesses to drive the cancer cell population to extinction (Gatenby and Brown 2020).

The main differences between conventional cancer treatment strategies and multi-strike therapy are in the timing of the strikes and the use of evolutionary principles to guide clinical decision making. In combination therapy, multiple drugs with collateral sensitivities are administered simultaneously (Chakrabarti and Michor 2017). In conventional sequential therapy, a second treatment is typically started during relapse, when the first treatment has evidently failed. In multi-strike therapy (also known as extinction therapy Gatenby and Brown 2020), the idea is instead to switch treatments when the tumor is at its weakest, when it may well be clinically undetectable. The success rate of multi-strike therapy is expected to be highly sensitive to the timing and severity of the second and any subsequent strikes. Based on computational modeling, Gatenby et al. (2020) have suggested that the best time to switch to the second treatment occurs while the tumor is still shrinking in response to the first treatment.

Demonstrating its potential to improve cure rates across diverse cancer types, multi-strike therapy is being investigated in three small clinical trials. A Phase 2 trial using conventional chemotherapy drugs in metastatic rhabdomyosarcoma started recruiting patients in 2020 and is expected to run until 2026 (NCT04388839). A Phase 1 trial in metastatic prostate cancer (2022-27) involves agents that exploit the hormone sensitivity of cancer cells (NCT05189457). A Phase 2 trial using targeted therapies in metastatic breast cancer began in 2024 (NCT06409390). Further trials are in development.

Yet, despite this rapid progress to clinical evaluation, many critical questions regarding the timing of the subsequent strikes, the time until extinction, the effect of environmental and demographic factors, and most importantly, the conditions under which multi-strike therapy is a feasible alternative to other therapies remain unanswered. What is the probability that a population is rescued either by preexisting mutants or those that arise during the treatment? How can we maximize the probability of the tumor being eliminated with the second strike? How do outcomes vary with the cost of resistance, density dependence, and other factors that affect clonal growth rates?

We tackle these pressing questions in two ways. First, using ideas from evolutionary rescue theory, we develop and study the first analytical model of two-strike therapy. This simple, tractable mathematical model enables us to compute extinction probabilities and to identify the optimal time for the second strike. Second, we use extensive stochastic simulations to test the robustness of our analytical results and to study the effects of additional factors. Our model only focuses on cases where single-strike therapy and other conventional therapies result in tumor relapse with high probability. The aim is to investigate the feasibility of two-strike therapy when we know that conventional treatments fail to be curative. We thus establish a necessary foundation for further theoretical, experimental, and clinical investigations of multi-strike therapy.

## Methods

To obtain general, robust insights into the factors that determine a successful two-strike treatment strategy, we combine an analytical model and a stochastic simulation model. Both models involve two stressful environments (corresponding to the two treatment strikes) and four cell types. The first treatment (or strike) creates a stressful environment that we denote $E_{1}$. After switching to the second treatment, the tumor enters the second stressful environment, $E_{2}$. We do not take into account treatment toxicity. Cells can be sensitive to both treatments (*S*), completely resistant to one treatment but sensitive to the other ($R_{1}$ and $R_{2}$), or completely resistant to both treatments ($R_{1,2}$). The time to switch to the second treatment is *τ* and the population size at this time is N(τ). Therefore, when the population size reaches the switching threshold N(τ), the first treatment is stopped and the second treatment begins.

### Analytical methods

**Fig. 1.**
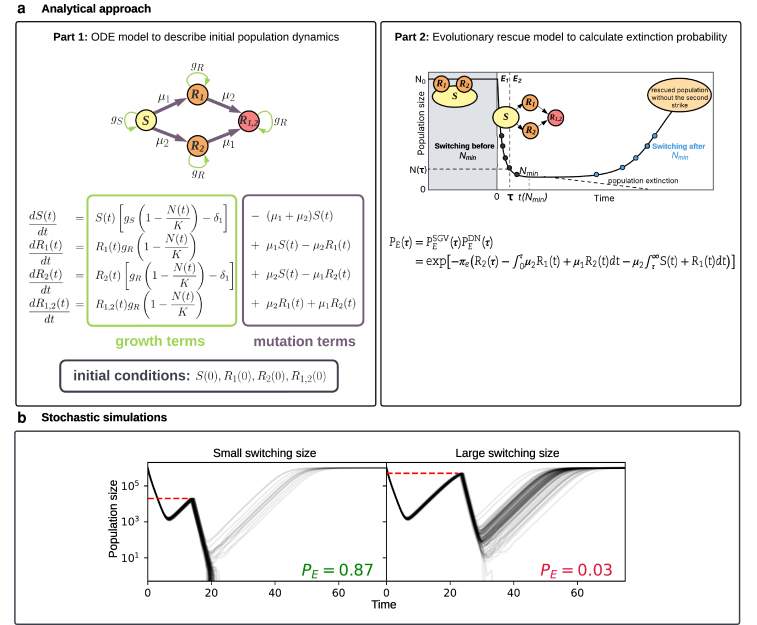
Description of the analytical approach and stochastic simulations. a) Part 1 (left) shows the ODE population growth model during the first treatment (in $E_{1}$). Part 2 (right) uses evolutionary rescue theory and output from the ODE model to calculate extinction probability after the second strike (in $E_{2}$). Sensitive cells are denoted by *S*. Cells resistant to treatment 1(2) and sensitive to treatment 2(1) are denoted by $R_{1}$($R_{2}$). Cells resistant to both treatments are denoted by $R_{1,2}$. The per capita rate of acquiring resistance to treatment 1(2) is denoted by $\mu_{1}(\mu_{2})$. Growth rates $g_{S}$ for sensitive cells and $g_{R}$ for resistant cells depend on the intrinsic birth rate, intrinsic death rate and the cost of resistance (see Table 1). The treatment-induced death rate, or treatment efficacy in $E_{1}$($E_{2}$) is denoted by $\delta_{1}$($\delta_{2}$). Initial conditions are specified by the initial population sizes of *S*, $R_{1}$, $R_{2}$ and $R_{1,2}$ cells (before the beginning of the first treatment). The total initial population N(0) is the sum of these four subpopulations. As shown in Part 2, the switch to the second treatment can occur before (dark dots on the curve) or after (paler dots) the population nadir ($N_{\min}$). The solid curve shows the population trajectory with treatment 1 only and the downward-sloping dashed line shows a possible trajectory to extinction after the switch to the second treatment at time *τ*. Equations to calculate extinction probability ($P_{E}$) are shown. b) Results of two sets of stochastic simulations are shown, illustrating the difference in extinction probabilities due to two different switching sizes (horizontal dashed lines). Extinction probabilities from the simulations are estimated for each switching size as the proportion of extinction outcomes in 100 independent runs. Pale curves show individual simulation runs. The stochastic simulation model is independent of the analytical approach, and is described in Methods.

Our analytical modeling method is composed of two stages (Fig. 1a). First, we model the population dynamics during the first treatment as a set of numerically-solved ordinary differential equations (ODEs). We then use those solutions to predict extinction probability using evolutionary rescue theory (as reviewed in Appendix B: Using results from evolutionary rescue theory). We thus use a deterministic model to represent a stochastic phenomenon. The interpretation is that the quantities S(t), $R_{1}(t)$, $R_{2}(t)$ and $R_{1,2}(t)$ in the ODE model are the expected numbers of *S*, $R_{1}$, $R_{2}$ and $R_{1,2}$ cells at time *t*, respectively. In Appendix D: Correspondence between the analytic evolutionary rescue model and the linear birth-death-mutation model, we verify that the dynamics of the ODE system and the mean dynamics of the corresponding stochastic process are in close agreement. Given this, we verify that the two approaches generate similar numbers of rescue mutants and similar extinction probabilities.

**Table 1.** List of parameters and initial conditions used in the analytical and stochastic simulation models, along with their default values.

Symbol	Description	Default value
*K*	Carrying capacity of the system	N(0)
*b*	Per capita birth rate of *S* cells	1.0
*d*	Per capita death rate of all cell types	0.1
*c*	Cost of resistance	0.5
$\mu_{1},\mu_{2}$	Mutation rate for acquiring resistance to treatment 1, 2	$2.5\times 10^{-6}$
$\delta_{1}$, $\delta_{2}$	Per capita death rate due to treatment 1,2	2.0
S(0)	Initial population of *S* cells	$10^{6}$
$R_{1}(0)$	Initial population of $R_{1}$ cells	100
$R_{2}(0)$	Initial population of $R_{2}$ cells	100
$R_{1,2}(0)$	Initial population of $R_{1,2}$ cells	0

Note that for the analytical model, we use the values of growth rates for sensitive and resistant cells, $g_{S}=b-d$ and $g_{R}=b-c-d$, respectively. The birth rate (*b*), death rate (*d*), cost of resistance (*c*) and rates of acquiring resistance ($\mu_{1},\mu_{2}$) are expressed in per-day units. For more details on parametrization, see Methods.

We use the following vocabulary. A potential rescue lineage is a lineage of cells with positive fitness at all future times. In our model these lineages comprise all $R_{1,2}$ cells (since they have a positive fitness in both $E_{1}$ and $E_{2}$), $R_{2}$ cells present at the switching time, and $R_{2}$ cells that arise after the switch. While $R_{1}$ cells have a positive fitness during treatment 1, they are counterselected during treatment 2 and so cannot rescue the population. Founders of potential rescue lineages are called potential rescue mutants. Potential rescue lineages that escape stochastic extinction and cause evolutionary rescue are called rescue lineages, and their founders are rescue mutants. Rescue mutants already present at the switching time *τ* are called *preexisting* rescue mutants, or *standing genetic variants*. Those appearing after are called *de-novo* rescue mutants.

To calculate the extinction probability of the population, we consider the expected number of rescue mutants generated throughout the course of treatment. We must therefore obtain the population composition at the beginning of the second strike. We use the system of differential equations given in Fig. 1a, describing logistic growth in environment $E_{1}$ of the four subpopulations S(t), $R_{1}(t)$, $R_{2}(t)$ and $R_{1,2}(t)$ that make up the tumor cell population N(t). The model includes mutations from less resistant to more resistant states while, for simplicity, omitting negligible back mutations.

For plausible parameter values, a tumor that grows from a single treatment-sensitive (*S*) cell is unlikely to harbor any doubly-resistant ($R_{1,2}$) cells at the time it is first treated (see Appendix A: Analytic model without competition for a reference case for initial resistant populations). If this were not so then extinction would be highly improbable. We therefore assume $R_{1,2}(0)=0$. Other default initial conditions and parameter values are listed in Table 1. By solving the differential equations numerically over the course of the first treatment (time 0 to time *τ*), we determine the expected subpopulation sizes at the time of switching to the second treatment.

Given the population dynamics using treatment switching time *τ*, we next compute the probabilities $P_{E}^{\mathrm{SGV}}(\tau )$ and $P_{E}^{\mathrm{DN}}(\tau )$. These are the probabilities of no evolutionary rescue due to standing genetic variation before the beginning of the second treatment and *de-novo* mutations during the second treatment, respectively (Part 2 in Fig. 1a). Since successful treatment requires the eradication of both preexisting and *de-novo* mutants during the second treatment period $E_{2}$, the tumor extinction probability $P_{E}(\tau )$ is the product of these two probabilities:

(1) $$\begin{array}{rl} P_{E}(\tau ) & =P_{E}^{\mathrm{SGV}}(\tau )P_{E}^{\mathrm{DN}}(\tau )\\ & =\exp\;\left[-\pi_{e}\left(R_{2}(\tau )+\int_{0}^{\tau }\mu_{2}R_{1}(t)+\mu_{1}R_{2}(t)dt+\mu_{2}\int_{\tau }^{\infty }S(t)+R_{1}(t)dt\right)\right], \end{array}$$

where $\pi_{e}$ is the probability of establishment of a single potential rescue lineage, that is, of a mutant lineage with positive fitness. This probability depends on the birth rate (*b*), death rate (*d*) and cost of resistance (*c*). See Appendix E: Derivation of the establishment probability and Appendix C: Derivation of extinction probabilities for more details on the derivation of Eq. 1. A key assumption is that the number of rescue mutants is Poisson distributed, which introduces the negative exponential in the expression. Eq. 1 also assumes that the probability of extinction during $E_{1}$ in the expression for total extinction probability is negligible. We verify this assumption at the end of Appendix A: Analytic model without competition, where we compare extinction probabilities from single-strike and two-strike therapies. This holds for all our parameter values.

With Eq. 1, we study the behavior of extinction probability as a function of *τ* under different conditions. The absolute value of the quantity in the exponent represents the total expected number of rescue mutants generated if we switch at time *τ*. The first term represents the expected number of $R_{2}$ cells present at the switching time, multiplied by the probability that they get established. The next term $\left(\pi_{e}\int_{0}^{\tau }\mu_{2}R_{1}(t)+\mu_{1}R_{2}(t)\;dt\right)$ represents the total expected number of established $R_{1,2}$ mutants generated by mutation from either $R_{1}$ or $R_{2}$ cells during $E_{1}$. The last term $\left(\pi_{e}\mu_{2}\int_{0}^{\tau }S(t)+R_{1}(t)\;dt\right)$ computes the expected number of established $R_{2}$ and $R_{1,2}$ cells generated by mutation from *S* and $R_{1}$ cells during $E_{2}$. The dynamics of the population during $E_{2}$ are modeled using the system of differential equations given in Eqs. C2–C3. There is no explicit $R_{1,2}$ term because we assume $R_{1,2}(0)=0$, and only the number of $R_{1,2}$ lineages (not the number of cells) generated throughout the entire treatment is required to calculate the extinction probability. Note that we do not compute the number of rescue mutants in the same way for $R_{1,2}$ cells and $R_{2}$ cells. As $R_{2}$ cells have a positive fitness only in environment $E_{2}$, we consider as potential founders of rescue lineages the $R_{2}$ cells that are present when switching to treatment 2 and those that arise later by mutation of *S* cells. By contrast, $R_{1,2}$ cells have positive fitness and the same establishment probability in both environments $E_{1}$ and $E_{2}$. Thus, the expected number of $R_{1,2}$ rescue mutants is simply this establishment probability multiplied by the expected number of $R_{1,2}$ lineages, which is the expected number of $R_{1,2}$ cells present at time 0 or arising by mutation, whether in $E_{1}$ or $E_{2}$.

In Appendix A: Analytic model without competition, we describe a model for two-strike therapy without competition. By removing the carrying capacity from the ODE model in Fig. 1a we obtain a system of linear differential equations which has an explicit solution (Eqs. A7–A9). Using this solution, we derive expressions for the expected number of rescue mutants (Eq. A12) and the population nadir. Since extinction probability is maximized when the expected number of rescue mutants is minimized, we find the optimal switching size and compare it to the nadir. We also compare our main results, which are derived under the assumption of a large initially resistant population, to a reference case corresponding to mutation-selection balance in an exponentially growing population. Further, we use this model to provide intuition for our results and examine the effects of changing model assumptions, such as removing the cost of resistance.

### Stochastic simulations

To test the robustness of our analytical results, we separately obtain extinction probabilities using a stochastic simulation model with the same initial conditions as the ODE system and equivalent default parameter values. The main difference between the models is that the analytical method uses evolutionary rescue theory to calculate extinction probabilities, whereas the computational approach uses the stochastic Gillespie algorithm to simulate birth, death, and mutation events. Density dependence is implemented in the birth rates used for the simulations. Each simulation ends with one of three outcomes: extinction, progression, or persistence (see Table G2). Progression is when the total population at the end of the simulation is greater than the total initial population. Persistence is when the population does not go extinct but is smaller than the initial population. The extinction probability is estimated for each switching size as the proportion of extinction outcomes in a large number of independent simulations. See Appendix G: Stochastic Simulation Model for a detailed description of the stochastic simulation model.

### Comparing results across parameter values

To compare treatment outcomes for varied parameter values, we use a summary variable $N_{q}$ to describe how small the tumor must be at the time of switching treatment to achieve a given probability of extinction. This concept is based on our observation that the extinction probability $P_{E}$(τ) generally decreases as N(τ) increases, unless N(τ) is very close to the population nadir ($N_{\min}$) that would pertain if we were to continue the first treatment instead of switching to a second treatment. Therefore, for a given extinction probability *q* (with $0\leq q\leq \max(P_{E}(\tau ))$), we can obtain a corresponding value $N_{q}$, which is the maximum population size threshold below which we achieve an extinction probability greater than or equal to *q*. In other words, if N(τ)$\leq N_{q}$, then we will achieve an extinction probability of at least *q*. Any given switching size N(τ) greater than $N_{\min}$ is reached twice in the trajectory of a population undergoing evolutionary rescue during the first treatment, once before and once after the start of relapse (see Fig. 1a, right for an illustration). $N_{q}$ is therefore defined for both the before-nadir and after-nadir switching sizes:

$$\begin{array}{rl} N_{q}^{\left(\mathrm{before}\right)} & =\max\{N(\tau )\;:\;P_{E}(\tau )\geq q,\tau <t(N_{\min})\},\\ N_{q}^{\left(\mathrm{after}\right)} & =\max\{N(\tau )\;:\;P_{E}(\tau )\geq q,\tau >t(N_{\min})\}, \end{array}$$

where $t(N_{\min})$ is the time at which the nadir would be reached in the absence of a second strike.

$N_{q}$ values tell us when and how fast the extinction probability drops from a high to a low value. For example, if the difference between $N_{0.1}^{\left(\mathrm{before}\right)}$ and $N_{0.9}^{\left(\mathrm{before}\right)}$ is slight, it means that the extinction probability goes from a low value to a high value without much change in the population size. Therefore, the extinction probability increases steeply with *N*. If $N_{0.1}^{\left(\mathrm{before}\right)}$ and $N_{0.9}^{\left(\mathrm{before}\right)}$ are far apart, there is a more gradual increase in extinction probabilities as the population size decreases. The value of *q* where the $N_{q}$ curve “cuts off” (as seen in Supplementary Fig. A.1) is the highest extinction probability possible with a given set of parameters. We plot $N_{q}$ versus *q* to analyze the trend in extinction probabilities across the range of potential switching sizes and parameter values.

When comparing different parameter values, a higher $N_{q}$ curve indicates better treatment outcome. This is because it is favorable to reach high extinction probabilities at large switching sizes. For instance, a higher value of $N_{0.9}$ implies a wider window of opportunity due to a wider range of switching sizes for implementing a successful second strike. Furthermore, if we have two $N_{q}$ curves corresponding to different sets of parameter values and one curve lies above the other, then in the case corresponding to the higher curve we can achieve a higher extinction probability for a given switching size (as illustrated in Supplementary Fig. A.1). The parameter values corresponding to the uppermost $N_{q}$ curve are therefore expected to result in a better treatment outcome in terms of ease of implementation, higher extinction probabilities, or both.

### Parametrization

The default parameters used for the stochastic simulations and numerical solutions are listed in Table 1. To facilitate comparison with previous research (Gatenby et al. 2020), we set the carrying capacity equal to the total initial population size, yet we also show that our results are highly robust to varying the carrying capacity. By default, we assume a large cost of resistance (half of the intrinsic birth rate), but we also study in detail the case in which resistance has no cost. We conservatively set the size of the initial resistant populations (before the first treatment begins) to $10^{-4}$ times the initial sensitive population, which is larger than the resistant population size predicted using a reasonable growth model (see Appendix A: Analytic model without competition for a reference case for initial resistant populations). We also consider the case $R_{2}(0)=0$.

The intrinsic birth and death rates are of the same order of magnitude as those reported in the literature and used in prior mathematical modeling (Gatenby et al. 2020). Since the rate of acquiring resistance (which we will call the mutation rate) varies depending on cancer type, treatment types, and patient attributes, we focus on a mutation rate corresponding to clinical scenarios in which two-strike therapy is worth considering. Much higher mutation rates render two-strike therapy ineffective, whereas lower mutation rates result in higher extinction probabilities (Supplementary Fig. A.7).

## Results

To enable us to uncover general principles and determine the most important factors in a successful treatment strategy, we consider the simplest case of two strikes. Since further strikes can only increase extinction probabilities, we thus obtain conservative lower bounds on potential clinical benefits. We obtain extinction probabilities using both an analytical evolutionary rescue model and a stochastic simulation model (see Methods), and we compare the two wherever possible. Unless stated otherwise, we use a default set of parameters and initial conditions (Table 1), and assume the two treatments induce identical death rates (that is, $\delta_{1}$=$\delta_{2}$=δ). For brevity, we will use “treatment efficacy” to refer to these treatment-induced death rates, which in reality also depend on pharmacodynamics and pharmacokinetics.

Our focus will be on the population size at the time of switching between the two treatments, denoted N(τ). Since the optimal N(τ) (the value corresponding to the highest extinction probability) changes as we vary parameter values, the trend of extinction probabilities obtained at a fixed N(τ) could differ from the trend obtained at the optimal N(τ). The rationale for using a fixed N(τ) for such comparisons is that it may in practice be impossible to determine the optimum. The fixed N(τ) can be implemented on either side of the population nadir reached in the absence of a second strike ($N_{\min}$). The population nadir can be calculated by numerically solving the system of differential equations shown in Fig. 1a (see Appendix A: Analytic model without competition for an analytical approximation). We consider both before-nadir and after-nadir switching sizes (Fig. 1a, right).

Throughout this section, we use two key metrics to compare different parameter values and treatment conditions. The first is the range of high-$P_{E}$ switching sizes, defined as the difference in the largest and smallest switching sizes that result in an extinction probability of at least 0.8. The second metric compares $N_{q}$ curves with the rule of thumb that a higher-lying curve indicates a better treatment outcome (see Supplementary Fig. A.1). As defined in Methods, $N_{q}$ is the maximum switching size to achieve an extinction probability greater than or equal to *q*.

### The optimal switching size is close to the population nadir

**Fig. 2.**
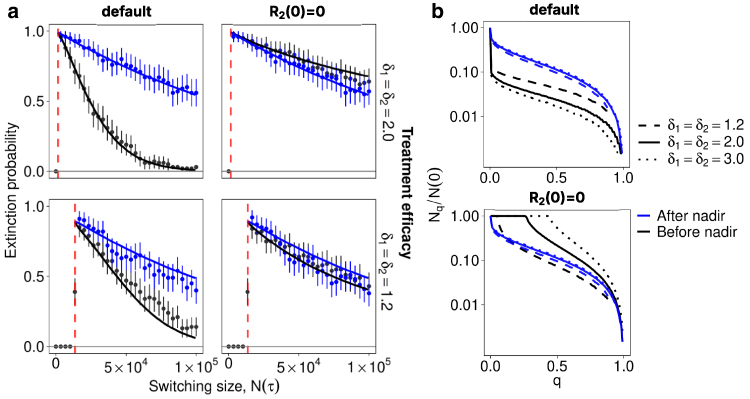
Optimal switching sizes. a) Extinction probabilities $P_{E}(\tau )$ obtained from the stochastic simulations (dots) and the analytical approach (solid line) for different switching sizes N(τ), implemented before reaching the nadir $N_{\min}$ (black) and after crossing $N_{\min}$ (blue). Red dashed lines show the expected $N_{\min}$ (calculated with the analytical model). Results for two treatment efficacies are shown (rows). Columns show results for the default parameter set (Table 1) and for the case when $R_{2}(0)=0$. Extinction probabilities from the simulations are estimated for each switching size as the proportion of extinction outcomes in 100 independent runs. In almost all simulations, switching sizes smaller than $N_{\min}$ prove unattainable, so the switch never occurs and the extinction probability is close to zero (black points to the left of red dashed lines). Error bars show 95% binomial proportion confidence intervals. b) Normalized $N_{q}$ vs *q* plots (described in Methods) for the default parameter set and for $R_{2}(0)=0$ with different treatment efficacies ($\delta_{1}$=$\delta_{2}$). Black curves show before-nadir switching sizes, and blue curves show after-nadir switching sizes.

Our first aim is to find the optimal population size, N(τ), at which to switch from the first to the second treatment. Our analytical and stochastic models both show that the optimal N(τ), in terms of maximizing extinction probability, is close to the population nadir $N_{\min}$ (Fig. 2). According to the analytical model, the optimal switching size may, depending on parameter values, lie slightly before or after $N_{\min}$ (Appendix A: Analytic model without competition). Yet the difference between the optimal N(τ) and the $N_{\min}$ is generally so small that it is not captured by our simulation results. The difference is significant only when $R_{2}$ cells are initially abundant and the cost of resistance is low (Supplementary Fig. A.2, second column).

To explain why the optimal N(τ) is close to $N_{\min}$, we refer to Eq. 1 and see that the maximum $P_{E}$(τ) will be achieved by minimizing the sum of all the rates of generating rescue mutants. Note that even though the sum of *S* and $R_{1}$ is minimized at $N_{\min}$, the *de-novo* rescue terms (the last two integral terms in Eq. 1) are minimized slightly after $N_{\min}$. This is because the increase in the number of cells after $N_{\min}$ is balanced by the faster decay of $R_{1}$ cells due to their cost of resistance. It follows that the *de-novo* rescue probability will be minimized when we have more $R_{1}$ cells and fewer *S* cells, even if the total number of cells is higher.

To minimize the rescue probability due to preexisting mutants at the start of the second strike (the first three terms in Eq. 1), we focus on the decay of the $R_{2}$ population and the emergence of $R_{1,2}$ mutants. Their relative rates determine where the switching size minimizing the number of preexisting rescue mutants falls in relation to $N_{\min}$. For further analysis, we focus on regions of high extinction probability ($P_{E}\geq 0.8$) instead of the exact optima. Consistent with our analytical predictions, we observe that these high-$P_{E}$ regions lie around $N_{\min}$ (Fig. 2 and Supplementary Fig. A.2).

### It is better to implement the second strike after the nadir than before

Given the practical impossibility of treating at the exact optimal time, we next compare outcomes for treating earlier or later. While the optimal switching size may lie slightly before or after the nadir, we observe that switching sizes implemented after $N_{\min}$ usually have higher extinction probabilities than those before $N_{\min}$ (Fig. 2a, left column and b, top panel).

We hypothesize that this result is due to the preexisting (or initially accumulated) $R_{2}$ population. If we delay switching, then this resistant subpopulation has longer to decay, which results in a smaller rescue population during the second treatment. On the other hand, there is more time for doubly-resistant $R_{1,2}$ mutants to accumulate. In most cases of interest, the generation of $R_{1,2}$ mutants is slower than the decay of the $R_{2}$ population, and so the window of opportunity for effective treatment extends further to the right of the nadir than to the left. In Appendix A: Analytic model without competition, we derive this result for the analytical model without competition.

Assuming that $R_{2}$ cells are absent at the start of the first treatment substantially increases extinction probabilities for before-nadir switching (Fig. 2a, right column and b, bottom panel; Supplementary Fig. A.2). The result of switching before the nadir is then similar to—or, in the case of a high-efficacy treatment, even slightly better than—the result of switching at the same tumor size after the nadir.

### Higher efficacy of the first treatment is not necessarily beneficial for treatment

Since treatment toxicity is a major concern in cancer therapy, we now relax our assumption that the two treatments have equal efficacy, enabling us to investigate the potential for using lower, more tolerable doses.

For alternative combinations of $\delta_{1}$ (efficacy of the first treatment) and $\delta_{2}$ (second treatment), we consider both the optimal switching size and the range of N(τ) values that lead to a high extinction probability ($P_{E}$ ≥0.8). We will refer to treatment efficacies of 2 and 1.2 (relative to the default intrinsic birth rate of sensitive cells) as high and low, respectively.

**Fig. 3.**
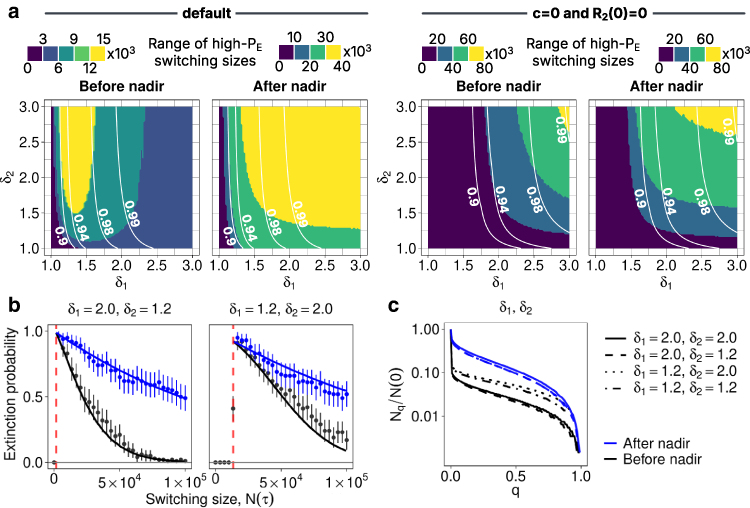
Optimal treatment combinations a) Heatmaps of the range of high-$P_{E}$ switching sizes ($P_{E}\geq 0.8$, obtained from the analytical model) for different combinations of treatment efficacies $\delta_{1}$ and $\delta_{2}$. The default case is shown on the left, and the case with no cost of resistance and no initial $R_{2}$ population is on the right. For each case, both before-nadir and after-nadir switching sizes are considered. White lines indicate optimal extinction probability contours (highest extinction probability across all switching sizes). Note that the color scales cover different ranges. b) Extinction probabilities for two combinations of treatment efficacies where $\delta_{1}$ ≠ $\delta_{2}$. Dots show simulation results and solid lines indicate analytical model predictions. Vertical dashed lines show the expected $N_{\min}$ (calculated with the analytical model). Before(after) nadir switching sizes are shown in black(blue). Extinction probabilities from the simulations are estimated for each switching size as the proportion of extinction outcomes in 100 independent runs. Error bars show 95% binomial proportion confidence intervals. c) Normalized $N_{q}$ vs *q* plots (see Methods) for four different treatment combinations. Black(blue) lines show before(after)-nadir switching sizes.

We first consider the before-nadir regime. The intuitive prediction is that a higher treatment efficacy should lead to a larger range of high-$P_{E}$ switching sizes. This is what we observe in the case of no resistance cost and $R_{2}$(0)=0 (Fig. 3a, right). However, for our default parameter values, higher values of $\delta_{1}$ give smaller ranges of high-$P_{E}$ switching sizes (Fig. 3a, left). Similarly, in the before-nadir regime, lower treatment efficacies result in higher extinction probabilities for all switching sizes that are not very close to $N_{\min}$ (Figs. 3b,c and 2b, top). A normalized $N_{q}$ versus *q* plot for four $\delta_{1}$-$\delta_{2}$ combinations confirms that low $\delta_{1}$ plus high $\delta_{2}$ produces the best treatment outcome in terms of the range of high-$P_{E}$ switching sizes, because it gives a higher extinction probability at the same N(τ) (Fig. 3c). When the two treatments are equally effective, we observe a similar trend (see Fig. 2b, top panel and Supplementary Fig. A.8c). Thus, for our default parameter values, a low-efficacy first treatment paired with a high-efficacy second treatment gives the largest window of opportunity when switching before the nadir.

The somewhat counter-intuitive result is explained by the interaction of the treatment efficacy, the cost of resistance, and the $R_{2}$ population. For lower $\delta_{1}$, the *S* cells decay more slowly, so the switching size N(τ) is reached later. This provides more time for the $R_{2}$ population to decay, but also more time for $R_{1,2}$ mutants to arise. When the cost of resistance and the initial $R_{2}$ population (before the first treatment begins) are both large, the benefit of a lower $\delta_{1}$ outweighs the disadvantages (see Appendix A: Analytic model without competition for a formal explanation). Therefore, we do not observe this effect when the cost of resistance or the initial $R_{2}$ population size is set to zero (Supplementary Fig. A.6, Fig. 3a, right). Note that if the first treatment’s efficacy is too low then the population size will never become small enough to have a nonnegligible probability of stochastic extinction.

Although a low $\delta_{1}$ gives a larger range of high-$P_{E}$ switching sizes, the highest extinction probability in all cases is obtained when both treatment efficacies are high (white contours in Fig. 3a). Therefore, it is important to define what we need for a good treatment outcome. The best treatment combination should not only lead to a high extinction probability at the optimal switching size, but it should also offer a large window of opportunity, that is, it should allow a large range of high-$P_{E}$ switching sizes. A low $\delta_{1}$ allows a larger window of opportunity than a high $\delta_{1}$ at the cost of compromising on the optimal extinction probability (0.94 compared to 0.99). Moreover, with a low $\delta_{1}$, the absolute values high-$P_{E}$ switching sizes are also high, because the $N_{\min}$ and the range of high-$P_{E}$ switching sizes are relatively large. We observe this in the right panel of Fig. 3b.

Note that this result depends on the fact that we compare ranges of high-$P_{E}$ sizes. The conclusion would be different if we were thinking in terms of switching times. A larger value of $\delta_{1}$ is expected to lead to a larger high-$P_{E}$ time interval. Thus, the best treatment efficacy in the before-nadir regime depends on how the therapy is implemented.

Outcomes for the after-nadir regime are best when both treatment efficacies are high, at least when thinking in terms of switching sizes (Fig. 3a, second panel; in terms of switching times, see Supplementary Fig. A.5). For our default parameter values, as expected, the range of high-$P_{E}$ switching sizes is also much larger for the after-nadir regime than for the before-nadir regime. When we eliminate the cost of resistance and the initial $R_{2}$ population, the optimal treatment combinations in the before-nadir and after-nadir regimes are similar (Fig. 3a, fourth panel).

### The cost of resistance is not necessarily beneficial for treatment

**Fig. 4.**
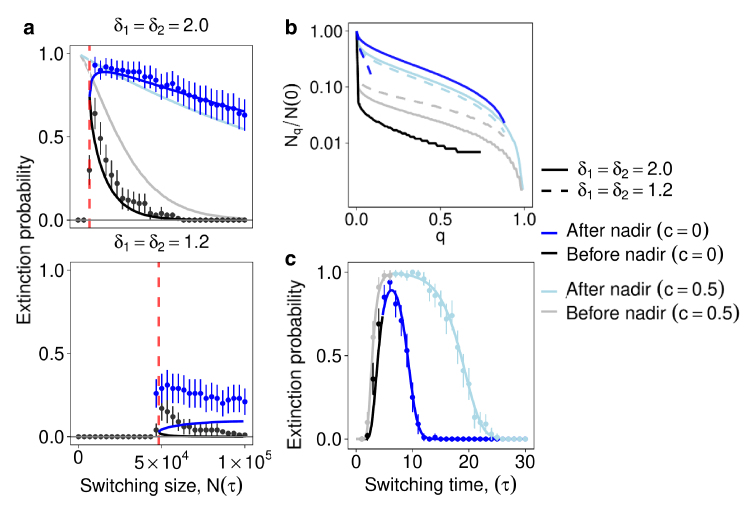
The effect of the cost of resistance. a) Extinction probabilities without a cost of resistance, for high (top) and low (bottom) treatment efficacies. Dots show simulation results and solid lines indicate analytical model predictions. Before(after) nadir switching sizes are shown in black(blue). Faded lines show the analytical estimates of extinction probabilities in the default case (c=0.5) for reference. Vertical dashed lines show the expected $N_{\min}$ (calculated with the analytical model). Extinction probabilities from the simulations are estimated for each switching size as the proportion of extinction outcomes in 100 independent runs. Error bars show 95% binomial proportion confidence intervals. b) Normalized $N_{q}$ vs *q* plots for two treatment efficacies and zero cost of resistance. Faded lines show the default case (c=0.5) for reference. The before-nadir curve for c=0 and δ=1.2 overlaps with the δ=2.0 curve, and ends at q=0.05. c) Time windows of high extinction probability. Solid lines are obtained from Eq. 1 under deterministic population dynamics with switching to the second drug at time *τ*. The dots with 95% confidence intervals are from 100-replicate Gillespie runs.

A cost of resistance is expected to hasten the decay of $R_{2}$ mutations during the first treatment phase and so make the second treatment more effective. Accordingly, in most cases, removing the cost of resistance reduces extinction probabilities (see Supplementary Figs. A.2 and A.7f). However, in the case of high-efficacy treatment (δ=2), extinction probabilities for switching sizes implemented well after the nadir can be slightly higher in the absence than in the presence of a resistance cost (but the optimal extinction probability is high even for severe resistance costs). This can be observed in the first panel of Fig. 4a and b. The reason for this counter-intuitive result is that in the absence of a cost of resistance and when switching after the nadir, fewer $R_{1,2}$ mutants are generated in the first treatment phase (see the section on the cost of resistance in Appendix A: Analytic model without competition for a more detailed explanation). If we fix the switching time instead of the switching size, we see that a higher resistance cost is always beneficial for treatment (see Fig. 4c).

For low-efficacy treatment (δ=1.2), the main effect of removing the cost of resistance is to increase $N_{\min}$, which makes it impossible to achieve high rates of extinction (Fig. 4a, second panel). We note here that although our analytical predictions are generally very close to our simulation results, they underestimate the probability of extinction when treatment efficacy is low and there is no cost of resistance. In this case, our modeling assumption of a Poisson-distributed $R_{2}$ population breaks down. This breakdown occurs when the variance of the $R_{2}$ population is higher than that of the corresponding Poisson distribution, which leads to a higher probability that there are no preexisting rescue mutants when the second treatment commences. Since turnover is a measure of demographic stochasticity, larger values of intrinsic turnover (b+d) at a constant intrinsic growth rate (b−d) lead to larger variance in the $R_{2}$ population and the breakdown of the Poisson assumption (see Appendix D: Correspondence between the analytic evolutionary rescue model and the linear birth-death-mutation model and Supplementary Fig. A.11 for more details).

### Two-strike therapy is feasible only in small tumors

**Fig. 5.**
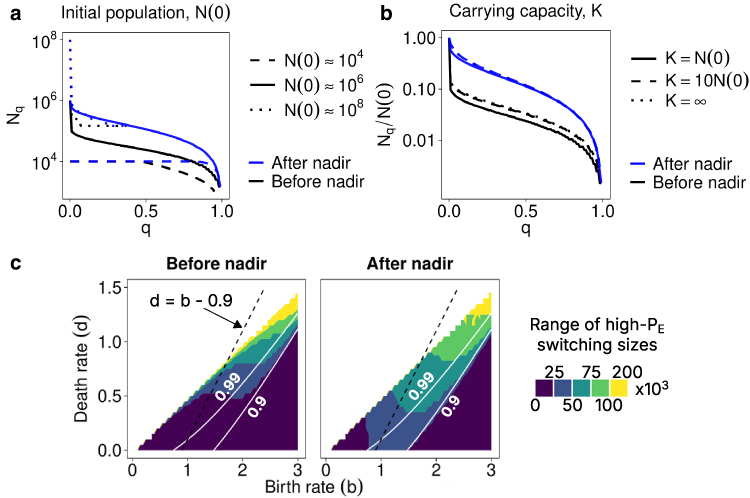
Effects of varying initial population, carrying capacity, birth and death rates. a) $N_{q}$ versus *q* plots for several initial population sizes with the same proportion of initial resistant cells. The *x*-axes show extinction probability threshold *q*, and *y*-axes are the $N_{q}^{\mathrm{before}}$ (black) and $N_{q}^{\mathrm{after}}$ (blue) values. Solid curves correspond to the default parameter values (Table 1). b) Normalized $N_{q}$ versus *q* plots for several carrying capacities. The curve for K=10N(0) overlaps with the curve for K=∞ (no competition). c) Heatmap of the range of high-$P_{E}$ switching sizes for different parameter values in *b*-*d* space. Only nonnegative growth rates (excluding the effects of treatment) are considered (d≤b−c). The dashed black line indicates the set of birth and death rates corresponding to our default growth rate ($b-d=g_{S}=0.9$). Solid white lines show optimal extinction probability contours (highest extinction probability across all switching sizes).

Using the analytical model, we compare different values of $N_{q}$ (not normalized) for different initial population sizes N(0), bearing in mind that the resistant population size scales with N(0). We observe that the absolute values of $N_{q}$ for *q* close to 1 do not vary by more than an order of magnitude when N(0) ranges over three orders of magnitude, from $10^{4}$ to $10^{7}$ cells (Fig. 5a). This implies that, within this range of initial tumor sizes, a high extinction probability can be achieved by applying the second strike at a sufficiently small population size (determined by the treatment efficacy, growth rates, and other parameters). Nevertheless, if N(0) is larger than $10^{8}$ cells then the extinction probability never exceeds 0.4 (Fig. 5a, dotted lines).

To achieve a 95% extinction probability after switching at the nadir using our default parameters and without competition (in the large *K* limit), the initial tumor must be no larger than around 7 million cells. To achieve the same extinction probability using single treatment therapy, the initial tumor must be three to four orders of magnitude smaller (see the last section in Appendix A: Analytic model without competition for an analytic derivation). There is, therefore, a limit on the size of tumors for which two-strike therapy is likely to succeed, but it is orders of magnitudes larger than for single-strike therapy.

### Extinction probability is insensitive to carrying capacity

As the carrying capacity is increased from N(0) (default value), we see a slight increase in extinction probability in the before-nadir regime, but this effect saturates around K=10N(0). This is demonstrated in Fig. 5b using the analytical model and in Supplementary Fig. A.8d with stochastic simulations. Systems with a lower *K* have an extra constraint on population growth since the initial population is closer to the carrying capacity. In our model, this results in a higher decay rate for *S* cells at the beginning of the first strike. Therefore, the switching size N(τ) (before nadir) is reached earlier and the expected $R_{2}$ population size is relatively larger when the switch occurs. This effect is not observed if the $R_{2}$ population is initially zero (Supplementary Fig. A.7c).

### Extinction probability increases with death rate and turnover

To compare treatment outcomes across different plausible combinations of birth and death rates, we plot heatmaps of the range of high-$P_{E}$ switching sizes in *b*-*d* space (Fig. 5c). In the lower right region (characterized by high birth rates and low death rates), extinction probabilities are very low. This leaves us with a diagonal band in the *b*-*d* space where high extinction probabilities become attainable. We define the “good” region as the area to the left of the the contour line corresponding to an optimal extinction probability of 0.9 (solid white line in Fig. 5c).

Within this “good” region, we make three major observations. First, a higher death rate results in a higher extinction probability (Supplementary Fig. A.7e). Second, as the birth rate increases, optimal extinction probability decreases and the optimal N(τ) increases (Fig. 5c, first panel and Supplementary Fig. A.7d). Faster growth rate of $R_{1}$ cells and slower decay of *S* cells due to increase in *b* leads to a larger $N_{\min}$ and therefore a larger optimal N(τ) (assuming that the optimal switching size is close to the nadir). The range of high-$P_{E}$ switching sizes within the “good” region is insensitive to changing the birth rate.

Third, we observe that the range of high-$P_{E}$ switching sizes becomes larger as we increase the turnover (defined as the sum b+d) while keeping the intrinsic growth rate $g_{S}$ constant (dashed line in Fig. 5b). Note that the cost of resistance is always a fixed fraction (0.5 by default) of the birth rate of *S* cells. It follows that when increasing turnover while keeping the growth rate $g_{S}$ constant, the growth rate $g_{R}$ of resistant cells decreases. This leads to a smaller resistant population, contributing to the increase in extinction probability. Another effect of turnover relates to the establishment probability of potential rescue lineages. As noted in Appendix E: Derivation of the establishment probability, turnover appears in the expression for estimating the establishment probability $\pi_{e}$. Higher turnover at a constant net growth rate $g_{S}$ leads to a lower $\pi_{e}$. Turnover also quantifies the influence of genetic drift, and it has been shown that increasing genetic drift reduces the probability of fixation of mutant cell types (Uecker and Hermisson 2011). If it is harder for potential rescue lineages to establish, then there will be fewer rescue lineages, leading to better treatment outcomes.

## Discussion

Here we have developed the first analytical model of a two-strike cancer therapy derived from the principles of evolutionary rescue. Our approach yields clearer explanations and more general results than previous computational modeling (Gatenby et al. 2020), in that we perform a more systematic analysis of the parameter space and we obtain extensive analytical results. We have also developed a complementary stochastic simulation model, which generally confirms the accuracy of our analytical predictions. To establish general conditions for successful therapy, we have sought to keep our models simple and conservative, for example by excluding Allee effects and assuming a relatively large initial population of resistant cells.

In terms of methodology, our study differs from the bulk of prior evolutionary rescue models that consider either one abrupt change in the environment or a continuous, gradual change (Bell 2017). While previous studies have examined evolutionary rescue when the environment fluctuates between harsh and favorable states (Greenspoon and Mideo 2017; Marrec and Bitbol 2020; Marrec and Bank 2023), we consider a sequence of distinct harsh states, such that the timing of environmental change is a controllable parameter. Most existing models of evolutionary rescue consider a single resistant variant or, in a few cases (Iwasa et al. 2004; Martin et al. 2013; Uecker and Hermisson 2016; Osmond et al. 2020), multiple variants with respect to one selective pressure. We have instead developed the case with three types of resistance to two types of environmental change, in the spirit of the classic chemotherapy model of Coldman and Goldie (1983).

We have used these new mathematical and computational models to investigate the optimal timing of the second strike and how the treatment outcome depends on crucial system parameters, including treatment efficacies and cost of resistance. The combination of analytical and computational analyses arms us with powerful tools to explore two-strike therapy in a wide range of scenarios, with a solid basis in eco-evolutionary theory. Several of our main conclusions are further supported by numerical results obtained using a different computational model, as reported in a preprint that was posted a year after our own study (Dabi et al. 2025).

***When do we get the highest extinction probabilities?*** The ability to analytically predict the optimal switching size for a large range of parameter values promises to aid the design of effective treatment schedules. In contrast to Gatenby et al. (2020), who suggested that striking before—even long before—$N_{\min}$ is better, we have shown that the optimal switching size is either slightly before or slightly after the nadir. Given that it is unreasonable to expect switching to the second strike exactly at the optimal size, we have shown that it is generally better to wait slightly longer and risk missing the optimal N(τ) than to apply the second strike too early. However, one should certainly not wait until the tumor becomes detectable again (as is the current practice) because that increases the probability that rescue mutants will emerge.

***Why do our results differ from those of Gatenby et al. (2020)?***

The model of multi-strike therapy investigated by Gatenby et al. (2020) differs from ours in important respects that account for our contrasting conclusions regarding the optimal timing of the second strike. In their model, the cell population is initially sensitive to the first treatment and resistance to this first treatment is a continuous trait, such that the tumor becomes increasingly resistant while the first treatment is applied. All tumors are assumed to be equally sensitive to the second treatment, which is modeled simply as an instantaneous 20% reduction in tumor size. Moreover, the first treatment is continued after the second strike.

In the absence of density-dependent effects, the second treatment in the model of Gatenby et al. (2020) effectively shifts the subsequent population size curve downwards by 20%. If this second strike is applied before $N_{\min}$ then the tumor size nadir will be 20% less than $N_{\min}$, rendering the tumor more susceptible to stochastic extinction. Applying the second strike when the tumor size is close to $N_{\min}$ is somewhat less effective than applying it earlier because in the latter case the tumor size spends more time below $N_{\min}$. Striking after $N_{\min}$ is worse still because by then the tumor is both larger and more resistant to the first treatment. Yet even if it is applied at the optimal time, the second strike’s impact on extinction probability will be negligible unless $N_{\min}$ happens to be very close to the stochastic extinction threshold.

Gatenby et al. (2020) instead found the second strike to be highly effective because they assumed that tumor growth is strongly density dependent. First, they assumed that, due to competition for resources, growth is inhibited at sizes close to the initial population size. Applying the second strike very early is therefore suboptimal as it reduces the benefit of this negative density dependence. Second, and much more consequentially, they assumed that an Allee effect inhibits growth at small population sizes, to the extent that the growth rate becomes negative at sizes moderately below $N_{\min}$, even for maximally resistant cells. Combined with the Allee effect, their second strike sufficed to shrink the tumor beyond the threshold at which extinction is not only probable but inescapable.

In summary, whereas the results of Gatenby et al. (2020) pertain to a specific scenario in which the first treatment drives the tumor to the brink of inevitable extinction and a small nudge can tip the balance, we have examined a more general case with more conservative assumptions. In our model, which has no Allee effect, the aim is not to push the tumor size below a given threshold but instead to minimize the probability that a rescue lineage will become established.

***What are the best treatment combinations?*** The treatment efficacies during the two strikes ($\delta_{1}$ and $\delta_{2}$) are the easiest model parameters to control in practice. The higher the two treatment efficacies, the higher the extinction probability at the optimal switching size (Fig. 3). However, switching at the optimal size, which becomes smaller as the treatment efficacy is increased, may be infeasible. In this case, the treatment combination that gives a wide range of switching sizes with a high probability of extinction may be better. Surprisingly, at least with a high cost of resistance, we found that the largest high-$P_{E}$ (≥0.8) region in the before-nadir regime is obtained with an low-efficacy first treatment paired with a high-efficacy second treatment. This result emphasizes the importance of timing in two-strike therapy—a stronger treatment with a poorly chosen switching time can be worse than a weaker treatment given at the right time. An interesting implication of this result is that the two treatments need not both be very effective. A low treatment-induced cell death rate can give good treatment outcomes if the cost of resistance is high. Moreover, the optimal switching size is also relatively high for a less effective first treatment, which may be beneficial for treatment in practice.

***What other tumor parameters determine the success of two-strike therapy?*** Our systematic exploration of the model parameter space reveals several noteworthy effects on treatment outcomes. First, although a high cost of resistance is predictably beneficial for treatment, we found that two-strike therapy can outperform conventional sequential treatment even when this cost is small or nonexistent (Fig. 4). Therefore, in common with adaptive therapy (Viossat and Noble 2021), two-strike therapy is not contingent on a cost of resistance. Moreover, we saw a variable response to a change in the cost of resistance depending on treatment efficacy. For high treatment efficacies, we observed comparable extinction probabilities in the presence and absence of the cost of resistance, but for low efficacies, a small cost of resistance gives much worse treatment outcomes than a high cost. Second, we find that higher intrinsic death rate and turnover are beneficial for treatment, consistent with findings that higher turnover increases extinction probability (Raatz and Traulsen 2023). This has also previously been shown for adaptive therapy (Strobl et al. 2020).

Third, we found that for a given initial tumor size, changes in carrying capacities have little effect on treatment outcome (Fig. 5b) even though a higher carrying capacity allows more tumor growth. This indicates that two-strike therapy could potentially be applied to both primary and metastatic cancers.

Although we have used simple models with minimal assumptions to ensure that our main findings are qualitatively robust, we have not explored all plausible functional forms. For example, in a model in which mutations occur only at the time of cell division, the number of potential rescue mutants (and therefore extinction probability) would depend on the number of divisions, while in our model it depends on the population size. The dependence of mutations on the birth rate would change some of our results, especially the results on death rate and turnover.

***When should two-strike therapy be used?*** Two-strike therapy holds most promise as an alternative to conventional sequential therapy—in which the second treatment is given only after the first has been seen to fail—especially in cases where a very good initial response to treatment is typically followed by relapse. We have shown it may be a wise choice when one of two available treatments is less effective than the other. Although our results suggest that two-strike therapy is likely to succeed only in relatively small tumors (Fig. 5a), we expect that subsequent treatment strikes, following the same principle, would lead to higher extinction probabilities for larger tumors. Our predicted extinction probabilities for two-strike therapy are moreover conservative as we have assumed a much larger initial resistant population than expected for our default tumor size, according to a standard model of mutation-selection balance (see Appendix A: Analytic model without competition). Allee effects might further increase extinction probabilities and make two-strike therapy viable in a wider range of scenarios (Dennis et al. 2016; Gatenby et al. 2020). Nevertheless, if resistant cells are abundant and have relatively high fitness then extinction may be practically unachievable and a long-term tumor control strategy such as adaptive therapy could be a better option (Gatenby et al. 2009; Monro and Gaffney 2009; Hansen and Read 2020; Viossat and Noble 2021).

When tolerable, combination therapy (that is, applying both treatments simultaneously) should also be considered. Whether it is better to apply drugs together or in sequence depends on several factors including the doses that can be administered in each case, the shape of the dose-response curves, the extent of synergism, and the degree of cross-resistance (Bozic et al. 2013; Saputra et al. 2018; Nyhoegen et al. 2024; Dabi et al. 2025). A comprehensive comparison of these alternative strategies using our mathematical methods is beyond the scope of the current study and remains an important topic for future research (but see Appendix F: Comparison with combination therapy).

Even when it may be theoretically optimal, two-strike therapy crucially depends on the availability of effective treatments with low cross-resistance and methods for monitoring tumor burden over time (Reed et al. 2020). Moreover, even when it is predicted to be the best strategy, some patients and physicians might be reluctant to initiate a second treatment before the first has been seen to fail.

Demonstrating the breadth of potential applications of two-strike therapy, the three clinical trials that are already underway in metastatic rhabdomyosarcoma (NCT04388839 2020), metastatic prostate cancer (NCT05189457 2021), and metastatic breast cancer (NCT06409390 2024) involve not only diverse cancer types but also very different classes of treatment, including chemotherapy, targeted therapies, and hormonal agents. Other proposed targets include locally advanced rectal adenocarcinoma (Felder et al. 2021) and pediatric sarcomas (Reed et al. 2020).

***Designing practical two-strike strategies.*** Given that the optimal switching size is reached some time after the tumor becomes undetectable, the design of practical switching strategies remains a challenging problem. One approach is to use a proxy for tumor size, such as prostate-specific antigen in the case of prostate cancer (NCT05189457 2021). But when personalized strategies are impractical it will be necessary to determine which standardized protocol performs best for a given cohort. Mathematical models and *in silico* trials have potential to facilitate this process by accounting for biological variation and clinical constraints as well as stochasticity (Dabi et al. 2025; Gallagher et al. 2025).

One idea is to extract tumor demographic rates by monitoring the decay in tumor volume during treatment (Grassberger et al. 2019), and to use sequencing data or experiments with patient-derived cells to estimate the initial size and growth rate of the resistant subpopulation. Using these parameter values, an estimator for when to switch could be constructed.

***Conclusion and future directions.*** We have shown that two-strike therapy is a theoretically sound concept that, in certain scenarios, could plausibly increase cancer cure rates compared to conventional single-strike or sequential treatment strategies. Our work provides a necessary foundation for further mathematical investigation and justification for experimental and clinical testing of this innovative strategy.

An important topic for further mathematical analysis is the prevention of evolutionary rescue with more than two strikes. Previous work on the optimal scheduling of multiple treatments (Goldie et al. 1982; Coldman and Goldie 1983; Chen et al. 2013) suggests that alternating two treatments is a theoretically sound approach. An alternative strategy, more in line with the original conception of multi-strike therapy, is to switch to a third treatment whenever possible. Other, related directions for mathematical investigation include accounting for cross-resistance and considering alternative biological assumptions, such as modeling resistance as a continuous, plastic trait.

## Supplementary Material

iyaf255_Supplementary_Data

iyaf255_Peer_Review_History

## Data Availability

The authors affirm that all data necessary for confirming the conclusions of the article are present within the article, figures, and tables. All relevant code is available in a public repository at https://doi.org/10.5281/zenodo.13332990. Supplementary material available at GENETICS online.

## Appendix A: Analytic model without competition

We study here the model without competition, obtained by letting the carrying capacity *K* go to infinity. This is a reasonable approximation of the true model (Fig. 5b and Supplementary Fig. A.8d), and it may be solved explicitly. This allows us to better understand the impact of various parameters.

We denote by $g_{1}$, $g_{2}$, and $g_{1,2}$ the baseline per-cell growth-rates of the resistant strains $R_{1}$, $R_{2}$ and $R_{1,2}$, respectively. In the main text, for simplicity, these growth rates are assumed to be equal and denoted by $g_{R}$, but this assumption is not needed here. Let $\gamma_{S}=g_{S}-\mu_{1}-\mu_{2}$, $\gamma_{1}=g_{1}-\mu_{2}$ and $\gamma_{2}=g_{2}-\mu_{1}$ denote the per-cell growth-rates of subpopulations *S*, $R_{1}$, and $R_{2}$, net of outgoing per-cell mutations rates. Let $c_{i}=\gamma_{S}-\gamma_{i}$ (i=1,2) denote the cost of resistance net of the mutation rates. Since the mutation rates are assumed to be much lower than the growth rates, $\gamma_{S}\approx g_{S}$, $\gamma_{i}\approx g_{i}$, and $c_{i}\approx g_{S}-g_{i}$, i=1,2.

**Table A.1.** Main symbols used in Appendix A.

Symbol	Description	Formula
$g_{S},g_{1},g_{2},g_{1,2}$	Per capita growth rates of *S*,$R_{1}$, $R_{2}$, and $R_{1,2}$ cells	
$\mu_{1},\mu_{2}$	Mutation rate for acquiring resistance to treatment 1, 2	
$\gamma_{S},\gamma_{1},\gamma_{2}$	Per capita growth rates net of outgoing mutation rates	$g_{S}-\mu_{1}-\mu_{2}$,$g_{1}-\mu_{2}$, $g_{2}-\mu_{1}$
$c_{1},c_{2},c_{1,2}$	Costs of resistance ($R_{1}$, $R_{2}$ or $R_{1,2}$ cells)	$\gamma_{S}-\gamma_{i}$
$\pi_{2},\pi_{1,2}$	Probability of establishment ($R_{2}$ or $R_{1,2}$ cells)	
${\tilde{R}}_{1}(0)$	Initial $R_{1}$ population needed to generate the same long-run $R_{1}$ population absent mutations from *S* to $R_{1}$	$R_{1}(0)+\frac{\mu_{1}S_{0}}{\delta_{1}-c_{1}}$
${\tilde{R}}_{2}(0)$	Additional number of $R_{2}$ cells at time 0 compared to reference case	$R_{2}(0)-\frac{\mu_{2}S_{0}}{c_{2}}$
$R_{1,2}^{*}(t)$	Expected number of $R_{1,2}$ lineages appearing before time *t*	
$t_{\min}$	Time at which the nadir is reached	
$N_{\min}$	Tumor size (number of cells) at the nadir	
$t_{\mathrm{DN}}$, $t_{\mathrm{SGV}}$	Switching time minimizing the number of *de-novo* or SGV rescue mutants	
$\lambda_{\mathrm{DN}}(\tau )$, $\lambda_{\mathrm{SGV}}(\tau )$	Expected number of *de-novo* or SGV rescue mutants if switching to treatment 2 at time *τ*	
$\alpha_{S}$	Coefficient of *S* in formula for number of rescue mutants	$\pi_{2}\left(\frac{1}{\delta_{2}-\gamma_{S}}+\frac{1}{c_{2}}\right)$
$\alpha_{1}$	Coefficient of $R_{1}$ in formula for number of rescue mutants	$\pi_{1,2}\left(\frac{1}{\delta_{2}-\gamma_{1}}+\frac{1}{\gamma_{1}}\right)$
$a_{S}$, $a_{1}$	Coefficient of *S* or $R_{1}$ in formula for number of *de-novo* rescue mutants	$\pi_{2}/(\delta_{2}-\gamma_{S})$,$\pi_{1,2}/(\delta_{2}-\gamma_{1})$

During the administration of treatment 1, the model reads

(A1 ) $$\dot{S}=(\gamma_{S}-\delta_{1})S$$

(A2 ) $${\dot{R}}_{1}=\gamma_{1}R_{1}+\mu_{1}S$$

(A3 ) $${\dot{R}}_{2}=(\gamma_{2}-\delta_{1})R_{2}+\mu_{2}S$$

(A4 ) $${\dot{R}}_{1,2}=g_{1,2}R_{1,2}+\mu_{2}R_{1}+\mu_{1}R_{2}.$$

A similar set of equations holds for population dynamics during treatment 2, with γ_S_ − δ_1_ replaced with γ_S_ − δ_2_, γ_2_ − δ_1_ replaced with γ_2_, and γ_1_ replaced with γ_1_ − δ_2_. The initial condition for the model during treatment 2 is simply $\left(S(\tau ),R_{1}(\tau ),R_{2}(\tau ),R_{1,2}(\tau )\right)$ where *τ* denotes the switching time.

In common with the analytical model of the main text, this is a deterministic model used to represent a stochastic phenomenon. The interpretation is that S(t), $R_{1}(t)$, $R_{2}(t)$ and $R_{1,2}(t)$ are the expected number of *S*, $R_{1}$, $R_{2}$ and $R_{1,2}$ cells at time *t*, respectively (see Appendix D: Correspondence between the analytic evolutionary rescue model and the linear birth-death-mutation model).

Recall that the potential rescue mutants (potential because they must become established to become true rescue mutants) are the $R_{2}$ cells present when switching to treatment 2, the $R_{2}$ cells arising by mutation from *S* to $R_{2}$ during treatment 2, as well as all $R_{1,2}$ cells arising directly by mutation of $R_{1}$ or $R_{2}$ cells (as opposed to reproduction of former $R_{1,2}$ cells), at any time: before treatment 1, during treatment 1, or during treatment 2. The expected number of potential $R_{1,2}$ rescue mutants appearing before time *t*, which we denote by $R_{1,2}^{*}(t)$, follows the dynamics

(A5 ) $${\dot{R}}_{1,2}^{*}=\mu_{2}R_{1}+\mu_{1}R_{2}.$$

The difference with the dynamics of $R_{1,2}$ cells is that the reproduction term is absent. We consider only newly generated $R_{1,2}$ mutants because the number of $R_{1,2}$ rescue mutants is determined by multiplying the probability of establishment by the number of $R_{1,2}$ cells that arise via mutation.

Letting $\pi_{2}$ and $\pi_{1,2}$ denote the probability of establishment of $R_{2}$ and $R_{1,2}$ mutants, respectively, the total expected number of rescue mutants if switching to treatment 2 at time *τ* is given by

(A6 ) $$\begin{array}{rl} RM(\tau ) & =\pi_{2}R_{2}(\tau )+\pi_{1,2}R_{1,2}^{*}(\tau )+\int_{\tau }^{+\infty }\mu_{2}(\pi_{2}S(t)+\pi_{1,2}R_{1}(t))\;dt\\ & \approx \pi_{2}R_{2}(\tau )+\pi_{1,2}R_{1,2}^{*}(\tau )+\pi_{2}\frac{\mu_{2}S(\tau )}{\delta_{2}-\gamma_{S}}+\pi_{1,2}\frac{\mu_{2}R_{1}(\tau )}{\delta_{2}-\gamma_{1}}, \end{array}$$

where the approximation neglects the impact of mutations from *S* to $R_{1}$ on the dynamics of $R_{1}$ cells during the administration of treatment 2. Note that due to the absence of competition in the above model, the probability of establishment of an $R_{1,2}$ mutant is the same in all environments, including before treatment.

(Not considering competition is most debatable before treatment begins, as this is when the tumor is expected to be most dense. Competition would reduce the probability of establishment of potential $R_{1,2}$ rescue mutants before treatment, but not taking this fact into account is harmless for two reasons. First, even without taking competition into account, the expected number of potential $R_{1,2}$ mutants arising before treatment 1, $R_{1,2}^{*}(0)$, is negligible. Lowering this value would only very slightly increase the probability of extinction for a given treatment. Second, if an $R_{1,2}$ rescue mutant gets established before treatment begins then the probability of tumor extinction is zero whatever the treatment. Thus, to determine which treatment maximizes the probability of extinction, we can condition on the event $R_{1,2}^{*}(0)=0$. The probability of establishment of $R_{1,2}$ cells before treatment begins is then irrelevant.)

If all populations of rescue mutants are approximately Poisson distributed, then the probability of extinction is approximately given by

$$P_{E}(\tau )=\exp\;(-RM(\tau )).$$

This is the analog of the main text Eq. (1), and explains why we want to minimize RM(τ).

Note that assuming that all populations of rescue mutants are approximately Poisson distributed is debatable: this is the case for all $R_{1,2}$ rescue mutants and for $R_{2}$ rescue mutants arising during treatment 2, but not necessarily for $R_{2}$ cells remaining when switching to treatment 2, see Distribution of preexisting established $R_{2}$ cells in Appendix D.

The system of differential equations (A1)–(A4) is linear and therefore has an explicit solution. Assuming $\gamma_{2}\neq \gamma_{S}$ and letting $S_{0}=S(0)$, we get

(A7 ) $$S(t)=S_{0}e^{-(\delta_{1}-\gamma_{S})t}$$

(A8 ) $$\begin{array}{rl} R_{1}(t) & =\left[R_{1}(0)+\frac{\mu_{1}S_{0}}{\delta_{1}-c_{1}}\left(1-e^{-(\delta_{1}-c_{1})t}\right)\right]e^{\gamma_{1}t}\\ & \approx \left[R_{1}(0)+\frac{\mu_{1}S_{0}}{\delta_{1}-c_{1}}\right]e^{\gamma_{1}t} \end{array}$$

(A9 ) $$\begin{array}{rl} R_{2}(t) & =\left[R_{2}(0)+\frac{\mu_{2}S_{0}}{c_{2}}\left(e^{c_{2}t}-1\right)\right]e^{-(\delta_{1}-\gamma_{2})t}\\ & =\left[R_{2}(0)-\frac{\mu_{2}S_{0}}{c_{2}}\right]e^{-(\delta_{1}-\gamma_{2})t}+\frac{\mu_{2}}{c_{2}}S(t), \end{array}$$

where the approximation for $R_{1}$ holds when $e^{-(\delta_{1}-c_{1})t}=(S(t)/S_{0}{)}^{1+\gamma_{1}/(\delta_{1}-\gamma_{S})}\ll 1$ (with our default parameters values, $e^{-(\delta_{1}-c_{1})t}=0.01$ when $S(t)/S_{0}\approx 0.034$).

The quantity $R_{1,2}(t)$ can also be expressed explicitly, but is not relevant. Indeed, it represents the expected number of $R_{1,2}$ cells, while we are interested in the expected number $R_{1,2}^{*}$ of $R_{1,2}$ cells directly arising from mutations from $R_{1}$ or $R_{2}$ cells. The latter is given by

(A10 ) $$R_{1,2}^{*}(\tau )=R_{1,2}^{*}(0)+\int_{0}^{\tau }\mu_{2}R_{1}(t)+\mu_{1}R_{2}(t)\;dt.$$

Here, $R_{1,2}^{*}(0)=\int_{-T}^{0}\mu_{2}R_{1}(t)+\mu_{1}R_{2}(t)\;dt$ where −T is the time of tumor initiation.

This leads to

(A11 ) $$\begin{array}{rl} R_{1,2}^{*}(t) & =R_{1,2}^{*}(0)+\mu_{2}\left[R_{1}(0)+\frac{\mu_{1}S_{0}}{\delta_{1}-c_{1}}\right]\left(\frac{e^{\gamma_{1}t}-1}{\gamma_{1}}\right)\\ & \;+\mu_{1}\left[R_{2}(0)-\frac{\mu_{2}S_{0}}{c_{2}}\right]\left(\frac{1-e^{-(\delta_{1}-\gamma_{2})t}}{\delta_{1}-\gamma_{2}}\right)\\ & \;+\mu_{1}\mu_{2}S_{0}\left(\frac{\delta_{1}-c_{1}-c_{2}}{c_{2}(\delta_{1}-c_{1})}\right)\left[\frac{1-e^{-(\delta_{1}-\gamma_{S})t}}{\delta_{1}-\gamma_{S}}\right], \end{array}$$

where the most important terms are those of the first line: the number of rescue mutants arising from $R_{1}$ and $R_{2}$ before the beginning of treatment and the number of rescue mutants arising from $R_{1}$ during treatment 1.

Letting ${\tilde{R}}_{1}(0)=R_{1}(0)+\mu_{1}S_{0}/\left(\delta_{1}-c_{1}\right)$, ${\tilde{R}}_{2}(0)=R_{2}(0)-\mu_{2}S_{0}/c_{2}$, and using (A9) and (A11), we obtain the following approximation of the expected number of rescue mutants when $S(t)/S_{0}=e^{-(\delta_{1}-\gamma_{S})\tau }\ll 1$, that is, when the sensitive population has been considerably reduced:

(A12 ) $$RM(\tau )\approx \mu_{2}\left[\alpha_{S}S(\tau )+\alpha_{1}R_{1}(\tau )\right]+\pi_{2}{\tilde{R}}_{2}(0)e^{-(\delta_{1}-\gamma_{2})\tau }+C,$$

where

(A13 ) $$\alpha_{S}=\pi_{2}\left(\frac{1}{\delta_{2}-\gamma_{S}}+\frac{1}{c_{2}}\right),\;\alpha_{1}=\pi_{1,2}\left(\frac{1}{\delta_{2}-\gamma_{1}}+\frac{1}{\gamma_{1}}\right)$$

and

(A14 ) $$\begin{array}{rl} C & =\pi_{1,2}(R_{1,2}^{*}(0)-\mu_{2}\frac{{\tilde{R}}_{1}(0)}{\gamma_{1}}+\mu_{1}\frac{{\tilde{R}}_{2}(0)}{\delta_{1}-\gamma_{2}}\\ & \;+\mu_{1}\mu_{2}S_{0}\left(\frac{\delta_{1}-c_{1}-c_{2}}{c_{2}(\delta_{1}-c_{1})}\right)\left[\frac{1}{\delta_{1}-\gamma_{S}}\right]) \end{array}$$

is a constant that impacts the probability of extinction but not the comparison between switching times. The quantity ${\tilde{R}}_{1}(0)$ represents the initial $R_{1}$ population that would generate the same long-run $R_{1}$ population in the absence of mutations from *S* cells to $R_{1}$. It is positive. The quantity ${\tilde{R}}_{2}(0)$ represents the additional initial $R_{2}$ population compared to a reference case described below. It may be positive or negative.

### A reference case for initial resistant populations

The initial resistant populations before the first treatment begins depend on the tumor and treatment history before the phase that we are interested in. For instance, if other treatments were given before and that resistance to these treatments is correlated to resistance to treatments 1 and 2, the initial resistant populations $R_{1}(0)$ and $R_{2}(0)$ could be large. Nevertheless, as a reference point, assume that the tumor starts at time −T with a single sensitive cell and no resistant cells, and that, until the administration of treatment 1, the tumor follows the dynamics we assumed, without the effect of treatment: $\dot{S}=\gamma_{S}S$ and ${\dot{R}}_{i}=\gamma_{i}R_{i}+\mu_{i}S$, i=1,2. For −T≤t≤0, this leads to

(A15 ) $$R_{i}(t)=\frac{\mu_{i}}{c_{i}}\left[e^{\gamma_{S}(T-|t|)}-e^{\gamma_{i}(T-|t|)}\right].$$

Letting t=0 and using that $e^{\gamma_{i}t}=[e^{\gamma_{S}t}{]}^{\gamma_{i}/\gamma_{S}}$, we obtain

(A16 ) $$R_{i}(0)=\frac{\mu_{i}}{c_{i}}\left[e^{\gamma_{S}T}-e^{\gamma_{i}T}\right]=\frac{\mu_{i}}{c_{i}}S_{0}\left[1-S_{0}^{-c_{i}/\gamma_{S}}\right]\approx \frac{\mu_{i}}{c_{i}}S_{0},$$

which corresponds to the standard mutation-selection balance in an exponentially growing population. This leads to a value of $R_{i}(0)$ equal to 5 with our default parameter values, much lower than the 100 initial $R_{i}$ cells we assume in our simulations.

In the absence of a cost of resistance: $c_{i}=0$, the previous formula becomes $R_{i}=\frac{\mu_{i}}{\gamma_{S}}S\ln\;S$, and with our other default parameter values, we obtain $R_{i}(0)\approx 38$, still lower than 100. If the number of mutations from *S* to $R_{i}$ is not only proportional to *S* but also to the birth rate of *S* cells, that is, ${\dot{R}}_{i}=\gamma_{i}R_{i}+\mu_{i}b_{S}S$, then $\mu_{i}$ should be replaced by $\mu_{i}b_{S}$ in the formula and we would get $R_{i}=\frac{\mu_{i}b_{S}}{\gamma_{S}}S\ln\;S$, or equivalently $R_{i}=\mu_{i}b_{S}St$. Note also that with our default parameter values, $S_{0}^{-c_{i}/\gamma_{S}}\approx 5\times 10^{-4}$, and the approximation in (A16) is excellent. It is less good for small costs of resistance, e.g. if $S_{0}=10^{6}$ and $c_{i}/\gamma_{S}=1/6$, then $S_{0}^{-c_{i}/\gamma_{S}}=0.1$ and we make a 10% error.

Plugging (A16) into (A5) and integrating between −T and 0 leads to

$$\begin{array}{rl} R_{1,2}^{*}(0) & =\sum_{i=1,2}\frac{\mu_{1}\mu_{2}}{c_{i}}\left[\frac{e^{\gamma_{S}T}-1}{\gamma_{S}}-\frac{e^{\gamma_{i}T}-1}{\gamma_{i}}\right]\\ & =\sum_{i=1,2}\frac{\mu_{1}\mu_{2}}{c_{i}}\left[\frac{S_{0}-1}{\gamma_{S}}-\frac{S_{0}^{\gamma_{i}/\gamma_{S}}-1}{\gamma_{i}}\right]\\ & \approx \mu_{1}\mu_{2}\frac{S_{0}}{\gamma_{S}}\left(\frac{c_{1}+c_{2}}{c_{1}c_{2}}\right). \end{array}$$

Plugging the initial resistant populations obtained in (A16) in the expressions for $R_{i}(t)$ and $R_{1,2}^{*}(t)$ of Eq. (A8), (A9) and (A11) leads to (during treatment 1)

$$\begin{array}{rl} R_{1}(t) & \approx \mu_{1}S_{0}\left[\frac{\delta_{1}}{c_{1}(\delta_{1}-c_{1})}\right]e^{\gamma_{1}t},\\ R_{2}(t) & \approx \frac{\mu_{2}}{c_{2}}S_{0}e^{-(\delta_{1}-\gamma_{S})t}=\frac{\mu_{2}}{c_{2}}S(t),\\ R_{1,2}^{*}(t) & \approx R_{1,2}^{*}(0)+\frac{\mu_{2}}{\gamma_{1}}R_{1}(t). \end{array}$$

This leads to

(A17 ) $$RM(\tau )\approx \mu_{2}\left[\alpha_{S}S(\tau )+\alpha_{1}R_{1}(\tau )\right]+\pi_{1,2}R_{1,2}^{*}(0),$$

where $\alpha_{S}$ and $\alpha_{1}$ were defined in (A13). The key difference with (A12) is that, since ${\tilde{R}}_{2}(0)\approx 0$, the term in ${\tilde{R}}_{2}(0)$ disappeared. In our simulations, we assume a larger initial resistant population than in this reference case (A16). This allows us to study the worst-case scenario in which the treatment history leads to a large resistant population, giving us conservative bounds on the extinction probability.

### Intuition about our findings, robustness

We now use previous formulas, with or without assuming the initial resistant populations of the reference case (A16), to compute some interesting quantities and get some intuition about our findings in the main text.

We first need a lemma.

Lemma A1 Let *A*, *B*, *α*, *β* be positive real numbers. The quantity $x(t)=Ae^{-\alpha t}+Be^{\beta t}$ is minimal at $t_{\min}=\frac{1}{\alpha +\beta }\ln\;(\frac{\alpha A}{\beta B})$ and its value is then

$$x_{\min}=\left(\frac{1}{\alpha }+\frac{1}{\beta }\right)(\alpha A{)}^{\beta /\left(\alpha +\beta \right)}(\beta B{)}^{\alpha /\left(\alpha +\beta \right)}.$$

Moreover, if C>0, the function $f(t)=Be^{\beta t}/Ae^{-\alpha t}$ satisfies $f(t)=Cf(t_{\min})$ when $e^{\left(\alpha +\beta )(t-t_{\min}\right)}=C$, and we then have

$$\frac{x(t)}{x_{\min}}=\frac{\beta }{\alpha +\beta }C^{-\alpha /\left(\alpha +\beta \right)}+\frac{\alpha }{\alpha +\beta }C^{\beta /\left(\alpha +\beta \right)}.$$

Proof. Let $y(t)=Ae^{-\alpha t}$, $z(t)=Be^{\beta t}$ so that x(t)=y(t)+z(t). The function *x* is convex and is thus minimal when its derivative cancels. Since $x^{\prime }(t)=-\alpha y(t)+\beta z(t)$, this occurs when z/y=α/β. This leads to $e^{(\alpha +\beta )t_{\min}}=\frac{\alpha A}{\beta B}$, hence the formula for $t_{\min}$. Moreover, z/y=α/β implies y/x=β/(α+β) and z/x=α/(α+β). Note that $f(t)=\frac{B}{A}e^{(\alpha +\beta )t}=f(t_{\min})e^{(\alpha +\beta )\Delta t}$ where $\Delta t=t-t_{\min}$. Thus, $f(t)=Cf(t_{\min})$ when $C=e^{(\alpha +\beta )\Delta t}$. We then have $y(t)=y(t_{\min})e^{-\alpha \Delta t}=y(t_{\min})C^{-\frac{\alpha }{\alpha +\beta }}$ and similarly, $z(t)=z(t_{\min})C^{\frac{\beta }{\alpha +\beta }}$. Therefore,

$$\begin{array}{rl} \frac{x(t)}{x_{\min}} & =\frac{y(t_{\min})C^{-\alpha /\left(\alpha +\beta \right)}+z(t_{\min})C^{\beta /\left(\alpha +\beta \right)}}{x_{\min}}\\ & =\frac{\beta }{\alpha +\beta }C^{-\alpha /\left(\alpha +\beta \right)}+\frac{\alpha }{\alpha +\beta }C^{\beta /\left(\alpha +\beta \right)}. \end{array}$$

#### Computation of the nadir

Unless we assume a negative resistance cost, we expect that at time 0 and throughout the administration of treatment 1, $R_{2}(t)\ll S(t)$ and $R_{1,2}(t)\ll R_{1}(t)$, so that most tumors cells are either *S* cells or $R_{1}$ cells. Thus,

(A18 ) $$N(t)\approx S(t)+R_{1}(t)\approx S_{0}e^{-(\delta_{1}-\gamma_{S})t}+{\tilde{R}}_{1}(0)e^{\gamma_{1}t}.$$

(Equation (A18) is similar to, e.g. Eqn 6 of Orr and Unckless (2014), with the difference that the latter does not consider the impact of mutations from *S* to $R_{1}$ cells arising during treatment 1.)

The right-hand side of Eq. (A18) may be minimized using Lemma A1. Letting ${\tilde{x}}_{1}(0)={\tilde{R}}_{1}(0)/S_{0}$, this leads to

$$N_{\min}\approx S_{0}\left[\frac{1}{\delta_{1}-\gamma_{S}}+\frac{1}{\gamma_{1}}\right](\delta_{1}-\gamma_{S}{)}^{\gamma_{1}/(\delta_{1}-c_{1})}{\left(\gamma_{1}{\tilde{x}}_{1}(0)\right)}^{\left(\delta_{1}-\gamma_{S})/(\delta_{1}-c_{1}\right)},$$

where we used that $\delta_{1}-\gamma_{S}+\gamma_{1}=\delta_{1}-c_{1}$. With the initial resistant populations of the main text and our default parameter values, we obtain $N_{\min}\approx 2018$. With the initial $R_{1}$ population of the reference case (A16) and our default parameter values otherwise, we would get $N_{\min}\approx 286$.

#### The expected number of *de-novo* rescue mutants is minimized by switching slightly after the nadir

This part of the analysis is also valid for the model with competition, as long as $N_{\min}\ll K$. The intuition is simple. Recall that *de-novo* rescue mutants are those generated during treatment 2, and let $a_{1}$ and $a_{S}$ denote the expected number of *de-novo* rescue mutants generated by each $R_{1}$ or *S* cell present at the switching time *τ*, respectively. The expected number of *de-novo* rescue mutants is minimized by switching when $a_{1}R_{1}+a_{S}S$ is minimal. In the absence of a resistance cost, $a_{1}=a_{S}$, and the expected number of *de-novo* rescue mutants is minimized by switching when $R_{1}+S$ is minimal, that is, essentially at the nadir. With a resistance cost, $R_{1}$ cells decay faster than *S* cells during treatment 2, therefore generating fewer *de-novo* rescue mutants. Thus $a_{S}>a_{1}$. For this reason, the expected number of *de-novo* rescue mutants is not minimized by switching at the nadir but slightly later (how much later depends on the resistance cost).

Let us be more precise. Let $\alpha =\delta_{1}-\gamma_{S}$ and $\beta =\gamma_{1}$. During treatment 1, $N\approx S+R_{1}$, $\dot{S}=-(\delta_{1}-\gamma_{S})S=-\alpha S$. Moreover, once $S(t)/S_{0}\ll 1$, ${\dot{R}}_{1}\approx \gamma_{1}R_{1}=\beta R_{1}$ (see Eq. (A8)), so $\dot{N}\approx -\alpha S+\beta R_{1}$. The nadir is thus reached, approximately, at the time $t_{\min}$ such that $R_{1}/S=\alpha /\beta$.

Let $a_{1}=\pi_{1,2}/(\delta_{2}-\gamma_{1})$ and $a_{s}=\pi_{2}/(\delta_{2}-\gamma_{S})$. The expected number $\lambda_{\mathrm{DN}}$ of *de-novo* rescue mutants satisfies

(A19 ) $$\begin{array}{rl} \frac{\lambda_{\mathrm{DN}}(\tau )}{\mu_{2}} & =\int_{\tau }^{\infty }\pi_{2}S(t)+\pi_{1,2}R_{1}(t)\;dt\\ & \approx a_{S}S(\tau )+a_{1}R_{1}(\tau )\approx a_{1}N(\tau )+(a_{S}-a_{1})S(\tau ), \end{array}$$

where we used that during treatment 1, $N\approx S+R_{1}$. In the absence of costs of resistance, that is, if $\gamma_{1}=\gamma_{2}=\gamma_{1,2}=\gamma_{S}$ (which also implies $\pi_{2}\approx \pi_{1,2}$ and therefore $a_{S}=a_{1}$), the quantity $\lambda_{\mathrm{DN}}(\tau )$ is minimized at the nadir. If, however, we assume a cost of resistance for $R_{1}$ cells and similar probabilities of establishments for $R_{2}$ and $R_{1,2}$ cells ($\pi_{2}\approx \pi_{1,2}$), then $a_{S}>a_{1}$. Since at the nadir, $\dot{N}=0$ and $\dot{S}<0$, it follows that $\lambda_{\mathrm{DN}}$ is still decreasing. To minimize it, it is thus best to switch after the nadir.

To be more precise, note that $\lambda_{\mathrm{DN}}$ is minimal at the time $t_{\mathrm{DN}}$ such that $a_{S}\dot{S}+a_{1}{\dot{R}}_{1}=0$, that is, $-a_{S}\;\alpha \;S+a_{1}\;\beta \;R_{1}=0$. It follows that

$$\frac{R_{1}(t_{\mathrm{DN}})}{S(t_{\mathrm{DN}})}=\frac{a_{S}}{a_{1}}\frac{\alpha }{\beta }=\frac{a_{S}}{a_{1}}\frac{R_{1}(t_{\min})}{S(t_{\min})}.$$

Using Lemma A1 with $C=\frac{a_{S}}{a_{1}}$ thus implies that

(A20 ) $$\frac{N(t_{\mathrm{DN}})}{N_{\min}}\approx \frac{\beta }{\alpha +\beta }(a_{S}/a_{1}{)}^{-\alpha /(\alpha +\beta )}+\frac{\alpha }{\alpha +\beta }(a_{S}/a_{1}{)}^{\beta /(\alpha +\beta )}.$$

With our standard parameter values, we get $N(t_{\mathrm{DN}})\approx 1.013N_{\min}$. The expected number of *de-novo* rescue mutants is minimized by switching just slightly after reaching the nadir. Testing other parameter values suggests that this conclusion is robust, in the sense that the ratio (A20) varies relatively little from 1 when changing parameters (Supplementary Fig. A.12).

Note also that with our default parameter values, $\delta_{1}-\gamma_{S}>\gamma_{1}$. That is, during treatment 1, the decay rate of the sensitive population is larger than the growth-rate of the $R_{1}$ population. Therefore, the expected number $a_{S}S+a_{1}R_{1}$ of *de-novo* rescue mutants tends to decrease rapidly before the nadir, and then to rebound more slowly. Therefore, in terms of switching times, the window of opportunity to minimize the number of *de-novo* rescue mutants extends further to the right than to the left of the nadir.

#### The expected number of preexisting rescue mutants may be minimized before or after the nadir

Recall that by preexisting or SGV potential rescue mutants, we mean $R_{2}$ cells present when switching to treatment 2, and $R_{12}$ cells arising directly from mutations of $R_{1}$ or $R_{2}$ cells before treatment 2. During treatment 1, the $R_{2}$ population decreases, but the expected number of $R_{1,2}$ rescue mutants increases. The expected number $\lambda_{\mathrm{SGV}}(\tau )=\pi_{2}R_{2}(\tau )+\pi_{1,2}R_{1,2}^{*}(\tau )$ of preexisting rescue mutants is minimal when these two forces exactly balance each other, i.e. when

$$\frac{d}{d\tau }\lambda_{\mathrm{SGV}}=0.$$

That is,

$$\pi_{2}\left[(\gamma_{2}-\delta_{1})R_{2}+\mu_{2}S\right]+\pi_{1,2}\left[\mu_{1}R_{2}+\mu_{2}R_{1}\right]=0.$$

Assuming $\mu_{1}\ll \delta_{1}-\gamma_{2}$, this happens when

$$R_{2}=\frac{\mu_{2}}{\delta_{1}-\gamma_{2}}\left[N+\frac{\left(\pi_{1,2}-\pi_{2}\right)}{\pi_{2}}R_{1}\right].$$

If, as in the main text, $\gamma_{1,2}=\gamma_{2}$, hence $\pi_{2}=\pi_{1,2}$, this boils down to

$$\frac{R_{2}}{N}=\frac{\mu_{2}}{\delta_{1}-\gamma_{2}}.$$

This may occur before or after the nadir, depending on the value of parameters, and in particular of the initial $R_{2}$ population: the larger $R_{2}(0)$, the more important it is to wait that the $R_{2}$ population decreases.

In the reference case (A16), that is, $R_{i}(0)=\mu_{i}S_{0}/c_{i}$, the expected number of preexisting rescue mutants is approximately

(A21 ) $$\lambda_{\mathrm{SGV}}=\mu_{2}\left[\frac{\pi_{2}}{c_{2}}S+\frac{\pi_{1,2}}{\gamma_{1}}R_{1}\right]+R_{1,2}^{*}(0).$$

Assuming ${\dot{R}}_{1}\approx \gamma_{1}R_{1}$, this is minimal when

$$\frac{R_{1}}{S}=\frac{\pi_{2}}{\pi_{1,2}}\frac{\delta_{1}-\gamma_{S}}{c_{2}}.$$

Recalling that at the nadir, $R_{1}/S=(\delta_{1}-\gamma_{S})/\gamma_{1}$, and that the ratio $R_{1}/S$ increases with time, it follows that the number of preexisting rescue mutants reaches its minimum before the nadir if

$$c_{2}>\frac{\pi_{2}}{\pi_{1,2}}\gamma_{1}$$

and after the nadir otherwise. With our default parameters, the cost of resistance is very strong, and this inequality is satisfied (0.5>0.4). Thus, the number of preexisting rescue mutants reaches its minimum before the nadir. More precisely, by Lemma A1 and using $C=\gamma_{1}\pi_{2}/(c_{2}\pi_{1,2})$, the expected number of preexisting rescue mutants is minimized at the time $t_{\mathrm{SGV}}$ such that $t_{\mathrm{SGV}}\approx 0.983t_{\min}$, and the tumor size is then $N(t_{\mathrm{SGV}})\approx 1.005N_{\min}$.

However, with a smaller cost of resistance $c_{2}$, the number of preexisting rescue mutants would reach its minimum after the nadir. The optimal switching time would then unambiguously be after the nadir, even though we assumed in this computation a much smaller initial $R_{2}$ population than in our simulations. Thus, the fact that the optimal switching time is after the nadir does not require a large initial $R_{2}$ population.

#### When to switch in total: before or after the nadir?

To see whether the optimal switching time is before or after the nadir, consider first the reference case (A16), where $R_{2}(0)=\frac{\mu_{2}}{c_{2}}S_{0}$. From Equations (A19) and (A21), we obtain that the expected number of potential rescue mutants is given by:

$$\begin{array}{rl} RM(\tau ) & =\lambda_{\mathrm{DN}}+\lambda_{\mathrm{SGV}}\\ & =\mu_{2}\left[\pi_{2}\left(\frac{1}{\delta_{2}-\gamma_{S}}+\frac{1}{c_{2}}\right)S+\pi_{1,2}\left(\frac{1}{\delta_{2}-\gamma_{1}}+\frac{1}{\gamma_{1}}\right)R_{1}\right]+\pi_{1,2}R_{1,2}^{*}(0)\\ & =\mu_{2}\left[\alpha_{S}S+\alpha_{1}R_{1}\right]+\pi_{1,2}R_{1,2}^{*}(0), \end{array}$$

where S=S(τ), $R_{1}=R_{1}(\tau )$, and $\alpha_{S}$ and $\alpha_{1}$ were defined in (A13). This is just another derivation of Eq. (A17), but making the contributions of *de-novo* and SGV rescue mutants more transparent. By the same logic as in the previous sections, it is best to switch before the nadir if the coefficient of *S* is smaller than the coefficient of $R_{1}$, that is, if $\alpha_{S}<\alpha_{1}$, and after otherwise.

With our default parameter values except for the initial resistant populations, $\alpha_{S}/\alpha_{1}\approx 0.93<1$, so it is best to switch slightly before the nadir. More precisely, from (A17), we get that the expected number of rescue mutants is minimized when $\alpha_{S}\dot{S}+\alpha_{1}{\dot{R}}_{1}=0$. Recalling that $\dot{S}=-(\delta_{1}-\gamma_{S})S$, that $\dot{R_{1}}\approx \gamma_{1}R_{1}$ once $S/S_{0}\ll 1$, and that at the nadir $R_{1}(t_{\min})/S(t_{\min})\approx (\delta_{1}-\gamma_{S})/\gamma_{1}$, this leads to

$$\frac{R_{1}}{S}\approx \frac{\alpha_{S}}{\alpha_{1}}\frac{R_{1}(t_{\min})}{S(t_{\min})}.$$

Denoting by $t_{\mathrm{opt}}$ the time at which the expected number of rescue mutants is minimized, we then get from Lemma A1 with $C=\frac{\alpha_{S}}{\alpha_{1}}$ that $t_{\mathrm{opt}}\approx 0.99994t_{\min}$ and that $N(t_{\mathrm{opt}})\approx 1.0005N_{\min}$. Thus, the optimal switching time is very slightly before the nadir, but so slightly that this could be due to approximation errors in our formulas, and could be easily reversed by slight changes in parameters. For instance, slightly lowering the resistance costs $c_{1}$ or $c_{2}$ would increase the ratio $\alpha_{S}/\alpha_{1}$, and for $c_{1}=c_{2}=0.47$, we already obtain that $t_{\mathrm{opt}}>t_{\min}$, i.e. we should now switch after rather than before the nadir.

We conclude that whether the optimal switching time is before or after the nadir is not a robust finding, and depends on parameters. What seems robust is that it is best to switch close to the nadir. Moreover, with our default parameter values, the decay-rate $\delta_{1}-\gamma_{S}$ of the sensitive population during treatment 1 is larger than the growth-rate $\gamma_{1}$ of the resistant population $R_{1}$. For this reason, tumor size and the number of expected rescue mutants tend to decrease relatively quickly before the nadir, and then to rebound more slowly (see mathematical details below). This makes switching after the nadir safer when the time at which the nadir is reached cannot be precisely determined (see Supplementary Fig. A.3). This conclusion would be reversed for parameter values such that $\delta_{1}-\gamma_{S}<\gamma_{1}$.

For arbitrary initial resistant populations, replacing (A17) by (A12) leads to:

$$\begin{array}{rl} RM(\tau ) & \approx \mu_{2}\left[\alpha_{S}N(\tau )+(\alpha_{1}-\alpha_{S})R_{1}(\tau )\right]+\pi_{2}{\tilde{R}}_{2}(0)e^{-(\delta_{1}-\gamma_{2})\tau }+C. \end{array}$$

The previous argument shows that, with our default parameter values, the bracket is minimized very slightly before the nadir. However, in our simulations, we chose a relatively large initial $R_{2}$ population, hence ${\tilde{R}}_{2}(0)$ is quite positive. Since the term ${\tilde{R}}_{2}(0)e^{-(\delta_{1}-\gamma_{2})t}$ decreases with time, switching after the nadir should give better results than switching before. This is what we observed (Fig. 2a, first row).

Here are details on the fact that the number of expected rescue mutants decreases quicker before the nadir than it rebounds after: we claim that in the reference case (A16), for any Δ>0, $RM(t_{\mathrm{opt}}+\Delta )\leq RM(t_{\mathrm{opt}}-\Delta )$. Indeed, let $S(t_{opt})$ and $R_{1}(t_{opt})$ denote the size of the *S* and $R_{1}$ populations at the optimal switching time $t_{\mathrm{opt}}$ if switching at (or after) this time. With the approximations ${\dot{R}}_{1}=\gamma_{1}R_{1}$ close to $t_{\mathrm{opt}}$ (before switching), and letting $\alpha =\delta_{1}-\gamma_{S}$, $\beta =\gamma_{1}$, we get that $\alpha \alpha_{S}S(t_{\mathrm{opt}})=\beta \alpha_{1}R_{1}(t_{\mathrm{opt}})$ and (recall A17):

$$\begin{array}{rl} \frac{1}{\mu_{2}}\left[RM(t_{\mathrm{opt}}+\Delta )-\pi_{12}R_{12}^{*}(0)\right] & =\alpha_{S}S(t_{\mathrm{opt}})e^{-\alpha \Delta }+\alpha_{1}R_{1}(t_{\mathrm{opt}})e^{\beta \Delta }\\ & =c\left[\frac{e^{-\alpha \Delta }}{\alpha }+\frac{e^{\beta \Delta }}{\beta }\right] \end{array}$$

where $c=\alpha \alpha_{S}S(t_{\mathrm{opt}})=\beta \alpha_{1}R_{1}(t_{\mathrm{opt}})$. It follows that for Δ>0,

$$\begin{array}{rl} & RM(t_{\mathrm{opt}}+\Delta )<RM(t_{\mathrm{opt}}-\Delta )\Leftrightarrow \frac{e^{-\alpha \Delta }}{\alpha }+\frac{e^{\beta \Delta }}{\beta }<\frac{e^{\alpha \Delta }}{\alpha }+\frac{e^{-\beta \Delta }}{\beta }\\ & \;\Leftrightarrow 2\frac{\sinh\;(\beta \Delta )}{\beta }<2\frac{\sinh\;(\alpha \Delta )}{\alpha }\Leftrightarrow \frac{\sinh\;(\beta \Delta )}{\beta \Delta }<\frac{\sinh\;(\alpha \Delta )}{\alpha \Delta }\Leftrightarrow \beta <\alpha \end{array}$$

where $\sinh\;(x)=(e^{x}-e^{-x})/2$ is the hyperbolic sine function, and the last equivalence follows from the standard fact that $x\to \frac{\sinh\;(x)}{x}$ is increasing on (0,+∞). Since $\alpha =\delta_{1}-\gamma_{S}>\gamma_{1}=\beta$, the result follows.

#### A lower value of $\delta_{1}$ may reduce the expected number of rescue mutants when switching before the nadir and at a given size

Treatment toxicity is a major concern in cancer therapy, which may justify the use of relatively mild doses. Here, we discuss whether there might be other motivations to reduce the efficacy of treatment 1.

For a given switching time, a larger value of treatment 1 efficacy $\delta_{1}$ is unambiguously beneficial for treatment: it leads directly to lower $R_{2}$ and sensitive populations, and indirectly to fewer mutations from sensitive to $R_{1}$ and $R_{2}$ cells. This in turn decreases the number of mutations from $R_{1}$ or $R_{2}$ cells to $R_{1,2}$ cells. Thus, a higher efficacy of treatment 1 decreases all resistant populations, leading to a lower probability of evolutionary rescue. (The situation would be more complex in a model with competition, as in the main text, because reducing the sensitive population would hasten the competitive release of the resistant populations. Thus, a larger value of $\delta_{1}$ would also have negative effects, and the net effect should be estimated more carefully. For example, with a lower $\delta_{1}$, the time before $R_{1}$ can grow at its competition-free rate increases, meaning that at a given time, there would be fewer $R_{1}$ cells, hence fewer $R_{12}$ mutants arising from $R_{1}$ cells, whether before or after switching to treatment 2. So, if switching at a relatively late time, when *S* cells and SGV $R_{2}$ mutants are insignificant, a lower value of $\delta_{1}$ could increase the probability of extinction. This can be seen by comparing the probability of extinction between $\delta_{1}=1.2$ and $\delta_{1}=2.0$ at otherwise default parameter values when switching at τ=20 (see Fig. A.5). Similar issues are studied by Day and Read (2016) and Uecker et al. (2014), e.g. pp. E31–E32, and in the literature on tumor containment (Viossat and Noble 2021).)

For a given switching size, a larger value of treatment 1 efficacy $\delta_{1}$ need not be beneficial for treatment, at least when we switch before the nadir, and the initial $R_{2}$ population is relatively large. Indeed, in that case, as mentioned in the main text, lower values of $\delta_{1}$ provide an advantage by letting the $R_{2}$ population decay more between the beginning of treatment 1 and the time when the switching tumor size is reached. We now explain this formally.

Assume that the protocol is to switch treatment the first time that tumor reaches size $\bar{N}$, and consider various values of $\delta_{1}$ such that this happens. As explained above, a lower value of $\delta_{1}$ leads to a lower rate of decay of the sensitive population, hence also to more mutations from *S* to $R_{i}$ and a higher growth rate of the resistant populations. It thus leads to a larger resistant population size and a larger tumor size for a given sensitive population size. It follows that to reduce tumor size to size $\bar{N}$, the sensitive population must be reduced by a larger factor when $\delta_{1}$ is low. Thus, letting $\tau (\delta_{1})$ denote the time at which the switching size $\bar{N}$ is reached, the ratio $S(\tau (\delta_{1}))/S_{0}$ is increasing with $\delta_{1}$ (lower for a low value of $\delta_{1}$).

But $S(\tau (\delta_{1}))/S_{0}=e^{-(\delta_{1}-\gamma_{S})\tau (\delta_{1})}$, so

$$\begin{array}{r} e^{-(\delta_{1}-\gamma_{2})\tau (\delta_{1})}={\left[S(\tau (\delta_{1}))/S_{0}\right]}^{\left(\delta_{1}-\gamma_{2})/(\delta_{1}-\gamma_{S}\right)}\;=\;{\left[S(\tau (\delta_{1}))/S_{0}\right]}^{1+c_{2}/(\delta_{1}-\gamma_{S})}. \end{array}$$

The lower $\delta_{1}$, the lower the quantity in the bracket, and the larger the exponent (assuming a cost of resistance). Thus a low value of $\delta_{1}$ leads to a low value of $e^{-(\delta_{1}-\gamma_{2})\tau (\delta_{1})}$, hence to a low value of the term ${\tilde{R}}_{2}(0)e^{-(\delta_{1}-\gamma_{2})\tau }$ when ${\tilde{R}}_{2}(0)>0$.

Since a low value of $\delta_{1}$ increases the proportion of $R_{1}$ cells when reaching size $\bar{N}$, the value of $\delta_{1}$ also affects the term $\alpha_{S}S+\alpha_{1}R_{1}=N[\alpha_{S}+(\alpha_{1}-\alpha_{S})R_{1}/N]$. However, as long as the ratio $\alpha_{S}/\alpha_{1}$ is close to 1, as with our default parameter values, this effect will be small.

Thus, for a relatively large value of the $R_{2}$ population, as in our simulations, we expect that the main effect of a decrease in the efficacy of treatment 1 *when switching at a given size and before the nadir* is to decrease the number of SGV $R_{2}$ rescue mutants. This explains why a lower efficacy of treatment 1 may then be paradoxically beneficial for treatment (Fig. 3a, left, and comparison of Figs. 2a, left, and 3b, right).

For similar reasons, a larger carrying capacity *K*, by reducing the decay rate of *S* cells, may decrease the number of SGV $R_{2}$ rescue mutants and lead to a larger probability of tumor extinction when switching at a given size before the nadir, as discussed in Results.

#### Impact of a cost of resistance

A key impact of costs of resistance, if they are incurred even before treatment begins, is to decrease the initial proportion of resistant cells. This was addressed when deriving the reference case (A16). By contrast, we focus here on the impact of costs of resistance for fixed initial resistant populations, as assumed in our simulations.

For a given switching time, all costs of resistance lead to a larger probability of tumor extinction. Indeed, they do not impact the sensitive population, and reduce resistant populations, hence the expected number of rescue mutants. It follows that any cost of resistance increases the range of switching times leading to a large probability of extinction. In a model with competition between tumor cells (such as in the main text), resistance costs, by reducing the number of resistant cells, would slightly increase the number of sensitive cells, but we still expect resistance costs to lead to larger probabilities of extinction for a given switching time, as observed in our simulations (Supplementary Fig. A.3, compare columns 1 and 2, see also Supplementary Fig. A.4).

For a given switching size, resistance costs for $R_{2}$ and $R_{1,2}$ mutants still lead to a larger probability of tumor extinction: they do not significantly affect the switching time (since during treatment 1, $N\approx R_{1}+S$), nor the proportion of $R_{1}$ and *S* cells at that time, but reduce the expected number of rescue mutants, through a quicker decay of the $R_{2}$ population in the first phase, and the reduction of the probability of establishment of potential rescue mutants in both phases.

The effect of a cost of resistance for $R_{1}$ cells when switching at a given size is more subtle. The coefficient $\alpha_{1}$ in (A12) is equal to:

(A22 ) $$\alpha_{1}=\pi_{1,2}\left(\frac{1}{\gamma_{1}}+\frac{1}{\delta_{2}-\gamma_{1}}\right)=\pi_{1,2}\frac{\delta_{2}}{\gamma_{1}(\delta_{2}-\gamma_{1})}.$$

In our model, the number of mutations from $R_{1}$ to $R_{1,2}$ cells is proportional to the $R_{1}$ population but not to its net growth rate. As a result, for a given resistant population $R_{1}(\tau )$, $R_{1}$ cells will generate more $R_{1,2}$ mutants in phase 1 when incurring a large cost of resistance.

Indeed, let *T* be the time at which the $R_{1}$ population reaches a given size. In our model, the expected number of $R_{1,2}$ cells generated by mutation of $R_{1}$ cells between 0 and *T* is $\int_{0}^{T}\mu_{2}R_{1}(t)\;dt=\mu_{2}T{\bar{R}}_{1}$ where ${\bar{R}}_{1}=\frac{1}{T}\int_{0}^{T}R_{1}(t)\;dt$ is the average size of the $R_{1}$ population until time *T*. A larger cost of resistance leads to a slower growth of the $R_{1}$ population, hence a larger duration *T* to grow up to a given size (i.e. more time to generate mutations). This thus leads to a larger number of mutations from $R_{1}$ to $R_{1,2}$. This effect would disappear if the number of mutations from $R_{1}$ to $R_{1,2}$ was assumed to be proportional not only to $R_{1}$, but also to its net growth-rate. The effect would be partially restored if the number of mutations was assumed proportional to the net birth rate.

The fact that $R_{1}$ cells incurring a large cost of resistance generate more $R_{1,2}$ cells in phase 1 is reflected in the fact that the term $1/\gamma_{1}$ in (A22) is a decreasing function of $\gamma_{1}$, hence an increasing function of $c_{1}$. They will, however, generate fewer $R_{1,2}$ mutants in phase 2, as a higher cost of resistance means a quicker decay of the $R_{1}$ population. This is reflected in the fact that the term $1/(\delta_{2}-\gamma_{1})$ in (A22) increases with $\gamma_{1}$. The net effect depends on whether $\gamma_{1}$ is smaller or larger than $\delta_{2}/2$ (indeed, the denominator $\gamma_{1}(\delta_{2}-\gamma_{1})$ in (A22) is maximal when $\gamma_{1}=\delta_{2}/2$). With our default parameter values, $\gamma_{1}<\delta_{2}/2$. Thus, a larger cost of resistance $c_{1}$ leads to a larger value of $\alpha_{1}$, as the dominant effect is that it leads to more $R_{1,2}$ mutants in phase 1.

When switching long after the nadir, the increase of $\alpha_{1}$ is the main effect of a cost of resistance $c_{1}$. Indeed, preexisting $R_{2}$ mutants should be rare, and the fraction of $R_{1}$ cells when switching is anyway close to 1, hence should vary little with $c_{1}$. A larger cost of resistance is then paradoxically detrimental for treatment.

We conclude that, though the net effect depends on parameter values, a larger cost of resistance $c_{1}$ might counter-intuitively decrease the probability of extinction when switching at a given size after the nadir, as discussed in Results.

Supplementary Figure A.4 shows the impact of varying the cost of resistance for the analytical model. Here, as in the main text, the same cost of resistance is applied to all resistant types: $c_{1}=c_{2}=c_{12}=c$. Supplementary Figure A.4 also considers the case of a negative cost of resistance, that is, when resistant cells are fitter than sensitive cells even in the absence of treatment (we did not run simulations of the stochastic model for the case of negative cost of resistance, so these results of the analytical model should be taken with care.) Supplementary Figure A.4b and A.4d illustrate that for a given switching time, the probability of extinction increases with the cost of resistance *c*. Supplementary Figure A.4a and A.4c illustrate that, for a given switching size, when switching long after the nadir, the probability of extinction may paradoxically decrease when the cost of resistance increases (though the optimal extinction probability increases). With a low or negative cost of resistance, the plots show that whether it is optimal to switch close to the nadir depends on the initial $R_{2}$ population. Indeed, if the cost of resistance $c_{2}$ is low or negative, the $R_{2}$ population decays more slowly during treatment 1. It may then be optimal to switch to treatment 2 substantially after reaching the nadir, in order to decrease the number of SGV $R_{2}$ rescue mutants (Supplementary Fig. A.4a). This effect disappears if we assume a low initial $R_{2}$ population (compare Supplementary Fig. A.4a and A.4c).

### Initial tumor sizes when one- and two-strike therapy are effective

Here, we examine how large the initial tumor can be whilst still maintaining effective treatment using one-strike and two-strike therapy. Consider the model without competition of Eq. (A1)–(A4) We model 1-strike therapy as following the same model but with a switching time τ=∞. Let $P_{0}=(S_{0},R_{1}(0),R_{2}(0),R_{1,2}(0))$ denote the vector of initial conditions of the reference case (A16). Denoting by $N_{0}$ the initial total tumor size and using that $N_{0}\approx S_{0}$, we have:

(A23 ) $$P_{0}\approx \left(N_{0},\frac{\mu_{1}}{c_{1}}N_{0},\frac{\mu_{2}}{c_{2}}N_{0},\mu_{1}\mu_{2}\frac{N_{0}}{\gamma_{S}}\left(\frac{c_{1}+c_{2}}{c_{1}c_{2}}\right)\right).$$

Thus, the initial conditions are (roughly) proportional to the initial tumor size $N_{0}$. Since the model consists of linear differential equations, it follows that *the solutions and the number of rescue mutants generated when striking at a given time are (roughly) proportional to* $N_{0}$. It also follows that in two-strike therapy, the optimal switching time *τ* is independent of $N_{0}$.

Let us compare 1-strike therapy to a 2-strike therapy switching at this optimal switching time *τ*. Let

$$P_{0}^{1}=\frac{P_{0}}{N_{0}}\approx \left(1,\frac{\mu_{1}}{c_{1}},\frac{\mu_{2}}{c_{2}},\mu_{1}\mu_{2}\frac{1}{\gamma_{S}}\left(\frac{c_{1}+c_{2}}{c_{1}c_{2}}\right)\right)$$

denote the vector of initial frequencies of the various types of tumor cells. For i=1,2, let $RM^{i}(P_{0})$ and $RM^{i}(P_{0}^{1})$ denote the number of rescue mutants generated by *i*-strike therapy if the initial condition is $P_{0}$ and if the initial condition is $P_{0}^{1}$, respectively. The formula used for 1-strike therapy is:

$$\begin{array}{rl} {\text{RM}}^{1} & =\pi_{e}R_{1,2}(0)+\pi_{e}R_{1}(0)\\ & \;+\pi_{e}\mu_{1}\int_{0}^{\infty }S(t)\;dt+\pi_{e}\mu_{2}\int_{0}^{\infty }R_{2}(t)\;dt, \end{array}$$

where the term $R_{1,2}(0)$ is negligible.

By the above emphasized observation ${\text{RM}}^{i}(P_{0})\approx N_{0}{\text{RM}}^{i}(P_{0}^{1})$. Fix a desired tumor extinction probability $p_{\mathrm{ext}}$. Let $N_{0}^{i}$ denote the tumor size leading exactly to this extinction probability under *i*-strike therapy. Then

$$p_{\mathrm{ext}}\approx \exp\;\left(-N_{0}^{i}\;{\text{RM}}^{i}(P_{0}^{1})\right).$$

Therefore, $N_{0}^{i}\;{\text{RM}}^{i}(P_{0}^{1})\approx -\ln\;(p_{\mathrm{ext}})$ hence $N_{0}^{1}\;{\text{RM}}^{1}(P_{0}^{1})\approx N_{0}^{2}\;{\text{RM}}^{2}(P_{0}^{1})$. Finally,

$$\frac{N_{0}^{2}}{N_{0}^{1}}\approx \frac{{\text{RM}}^{1}}{{\text{RM}}^{2}}.$$

This turns out to be on the order of $3\cdot 10^{3}$ using the reference case (and of $6\cdot 10^{3}$ using the default population distribution from Table 1), meaning much larger tumors can be eliminated at a desired probability using two-strike compared to one-strike therapy. The analysis would be trickier with the nonlinear model of the main text (with competition), but we expect the same order of magnitude.

## Appendix B: Using results from evolutionary rescue theory

While the conventional goal of evolutionary rescue is to maximize the probability of rescue, the same mathematical formulations can be applied with the aim of extinction. For our analytical model, we use the theory of evolutionary rescue to find the probability of extinction in cancer populations undergoing two-strike therapy. A population is rescued from extinction when one or more mutant lineages with positive fitness, which we call potential rescue lineages, become established in the population. The founders of potential rescue lineages are called potential rescue mutants. Those that establish in the population and lead to evolutionary rescue are called rescue mutants.

We assume that the generation of potential rescue mutants in the population can be approximated by a Poisson process (Alexander et al. 2014) (see Appendix D: Correspondence between the analytic evolutionary rescue model and the linear birth-death-mutation model for further investigation into the validity of this assumption in the context of our model). We use a diffusion approximation to obtain a general, continuous-time estimate of the establishment probability of potential rescue lineages (see Appendix E: Derivation of the establishment probability for the derivation). The establishment probability is multiplied by the rate of generation of potential rescue mutants to obtain the rate of generation of rescue mutants. Therefore, once the rate of the Poisson process is calculated, the probability of extinction is simply the probability that no rescue mutants are generated.

We assume density independence of potential rescue lineages and use branching processes to model the emergence of new resistant (and rescue) mutants (Orr and Unckless 2008). The growth model we use is described in Appendix E: Derivation of the establishment probability. Density independence is a reasonable assumption because rescue lineages predominantly establish when the population is well below the carrying capacity due to the effect of treatment. Additionally, we make the assumption that the probability of establishment of a potential rescue mutant is constant with respect to time, which is reasonable assuming that competition does not much affect establishment (Supplementary Fig. A.13). Furthermore, there is extensive literature on what happens when these assumptions do not hold. Analytical results can be derived in all such situations using stochastic methods and taking continuous time approximations (Otto and Whitlock 1997; Martin et al. 2013; Uecker et al. 2014) or directly working with the linear branching process (Uecker and Hermisson 2011).

An important distinction is that the probability of rescue by preexisting mutants (from before the onset of treatment, called standing genetic variation) is different from that of new mutants (via de-novo mutations) (Orr and Unckless 2008). This is because the preexisting mutant (SGV) lineages typically get more time to grow than the de-novo mutants (DN). There are several expressions by different authors (Orr and Unckless 2008; Martin et al. 2013; Uecker et al. 2014) for the probability of evolutionary rescue following a single change in environment (treatment strike in our case) by both these classes of mutants, but considering the common conceptual basis, they all reduce to the following form:

(B1 ) $$P_{\mathrm{ER}}^{\mathrm{SGV}}=1-\exp\;[-N(0)\pi_{e}f(0)]$$

(B2 ) $$P_{\mathrm{ER}}^{\mathrm{DN}}=1-\exp\;\left[-\mu \pi_{e}\int_{0}^{\infty }N(t)dt\right],$$

where N(0) is the population size at the onset of treatment (t=0), *μ* is the total per capita, per unit time mutation rate during treatment, and f(0) is the frequency of resistant variants at t=0. Thus, expressions B1 and B2, respectively, represent the probability of evolutionary rescue from the established resistant population already present at the onset of treatment at t=0 and from the established resistant population produced by mutation from *N* during treatment. While we use the probability mass function of the Poisson distribution to obtain B1, an equivalent expression is derived in Orr and Unckless (2008) from an approximation of $1-(1-\pi_{e}{)}^{N(0)f(0)}$, which holds for small $\pi_{e}$ and large N(0)f(0).

In our analytical approach, we use these results extensively in the context of using two-strike therapy to prevent evolutionary rescue.

## Appendix C: Derivation of extinction probabilities

Given the populations *S*, $R_{1}$, $R_{2}$ and $R_{1,2}$ at time *τ*, we first compute the probability of no evolutionary rescue due to preexisting rescue mutants (mutants present at the time of the second strike, also called standing genetic variation). From evolutionary rescue theory (Appendix B: Using results from evolutionary rescue theory), we know that the distribution of preexisting rescue variants can be reasonably approximated by a Poisson distribution with a rate proportional to the number of rescue mutants at the beginning of the second strike (see Appendix D: Correspondence between the analytic evolutionary rescue model and the linear birth-death-mutation model and Supplementary Fig. A.11 for exceptions). Thus, we can obtain a good estimate of the rescue probability due to preexisting $R_{2}$ mutants. However, to estimate the rescue probability due to $R_{1,2}$ mutants, we must consider the entire duration of the treatment because these cell types are resistant in both environments ($E_{1}$ and $E_{2}$). Therefore, in our model, the distribution of preexisting (at the time of the second strike) $R_{2}$ and $R_{1,2}$ resistant cells is Poisson with a rate equal to $\lambda^{\mathrm{SGV}}=\pi_{e}(R_{2}(\tau )+\int_{0}^{\tau }\mu_{2}R_{1}(t)dt+\int_{0}^{\tau }\mu_{1}R_{2}(t)dt)$, where $\pi_{e}$ is the probability of establishment of a single potential rescue lineage and $\mu_{1}$ and $\mu_{2}$ are the rates of acquiring resistance to treatments 1 and 2. The probability of establishment depends on birth rate (*b*), death rate (*d*) and cost of resistance (*c*). See Appendix E: Derivation of the establishment probability for the derivation. The establishment probability is assumed to be the same across time and for all resistant cell types. Following the Poisson assumption, the probability that all preexisting mutants go extinct in $E_{2}$ is equal to $P_{E}^{\mathrm{SGV}}(\tau )=\exp\;[-\lambda^{\mathrm{SGV}}]$.

To find the probability that no *de-novo* rescue mutants arise in $E_{2}$, we assume that the generation of new mutants is a Poisson process, and the number of rescue mutants in $E_{2}$ is Poisson distributed with a rate equal to $\lambda^{\mathrm{DN}}=\pi_{e}\mu_{2}\int_{\tau }^{\infty }S(t)+R_{1}(t)dt$. This rate is proportional to the rate of mutation from the sensitive cell fraction (*S* and $R_{1}$) to the resistant cell fraction ($R_{2}$ and $R_{1,2}$) in $E_{2}$.

The total extinction probability as a function of *τ* is given by the product of the probability of extinction of preexisting and *de-novo* mutants,

(C1 ) $$\begin{array}{rl} P_{E}(\tau ) & =P_{E}^{\mathrm{SGV}}(\tau )P_{E}^{\mathrm{DN}}(\tau )=\exp\;\left[-\pi_{e}R_{2}(\tau \right)\\ & \;-\pi_{e}\left(\int_{0}^{\tau }\mu_{2}R_{1}(t)dt+\int_{0}^{\tau }\mu_{1}R_{2}(t)dt\right)\\ & \;\;-\pi_{e}\mu_{2}\left(\int_{\tau }^{\infty }S(t)dt+\int_{\tau }^{\infty }R_{1}(t)dt\right)]. \end{array}$$

The quantity in the exponent represents the total expected number of rescue mutants generated if we switch at time *τ*. The first term represents the number of established $R_{2}$ cells at the switching time. The next two terms $\left(\pi_{e}(\int_{0}^{\tau }\mu_{2}R_{1}(t)\;dt+\int_{0}^{\tau }\mu_{1}R_{2}(t)\;dt)\right)$ represent the total expected number of established $R_{1,2}$ mutants generated by mutation from either $R_{1}$ or $R_{2}$ cells during $E_{1}$. The last two terms $\left(\pi_{e}\mu_{2}(\int_{0}^{\tau }S(t)\;dt+\int_{0}^{\tau }R_{1}(t)\;dt)\right)$ compute the number of established $R_{2}$ and $R_{1,2}$ cells generated by mutation from *S* and $R_{1}$ cells during $E_{2}$. There is no explicit $R_{1,2}$ term because we assume $R_{1,2}(0)=0$, and only the number of $R_{1,2}$ mutants generated throughout the entire treatment is required to calculate the extinction probability. To make the computation easier, we calculate the approximate values of the last two integral terms by integrating them until the corresponding population size is equal to one. For example, we numerically integrate the term $\int_{\tau }^{\infty }S(t)dt$ until S(t)=1. This is a good estimate of integrating till infinity.

To compute the expression in (C1) numerically, we solve the deterministic logistic growth equations for dynamics in $E_{1}$ (from t=0 to *τ*, given in Fig. 1a) and in $E_{2}$ (from t=τ till extinction, given below). In (C2) and (C3), we ignore the mutations to $R_{2}$ and $R_{1,2}$ and the changes in these resistant populations because we use only the deterministic decay of the sensitive population to calculate extinction probabilities.

(C2 ) $$\frac{dS(t)}{dt}=S(t)\left[g_{S}\left(1-\frac{N(t)}{K}\right)-\delta_{2}\right]-S(t)\mu_{1},$$

(C3 ) $$\frac{dR_{1}(t)}{dt}=R_{1}(t)\left[g_{R}\left(1-\frac{N(t)}{K}\right)-\delta_{2}\right]+S(t)\mu_{1}.$$

## Appendix D: Correspondence between the analytic evolutionary rescue model and the linear birth-death-mutation model

Here, we connect the ODE system dynamics to the dynamics of the mean of the stochastic model and show that RM(τ) corresponds to the mean number of rescue mutants generated in the stochastic model. Finally, we justify when formulas for the probability of extinction hold.

Throughout, $N_{S},N_{R_{1}},N_{R_{2}},N_{R_{1,2}}$ refer to the random variable counting the number of $S,R_{1},R_{2},R_{1,2}$ cells, respectively.

### ODE system dynamics approximately follows the mean dynamics of the stochastic model

#### Without competition

Without competition, it is exactly true that $E[N_{S}](t)=S(t),$ $E[N_{R_{1}}](t)=R_{1}(t),E[N_{R_{2}}](t)=R_{2}(t),E[N_{R_{1,2}}](t)=R_{1,2}(t)$

To show this, consider the joint PMF of the system $P(t;x_{0},x_{1},x_{2},x_{3})=P(N_{S}(t)=x_{0},N_{R_{1}}(t)=x_{1},N_{R_{2}}(t)=x_{2},N_{R_{1,2}}(t)=x_{3})$:

(D1 ) $$\begin{array}{rl} \frac{dP}{dt}(t;x_{0},x_{1},x_{2},x_{1,2}) & =\text{birth events}+\text{death events}\\ & \;+\text{mutation events}. \end{array}$$

Since there is no competition, the per capita event rates are independent of the state variables $x_{i}$. Birth events consist of

$$\begin{array}{rl} & \sum_{i=0}^{3}\delta_{0,x_{i}}^{k}(b-c(1-\delta_{0,i}^{k}))(x_{i}-1)P(t;x_{0},\ldots ,x_{i}-1,\ldots ,x_{3})\\ & \;-b_{i}x_{i}P(t;x_{0},\ldots ,x_{3}), \end{array}$$

where $\delta_{i,j}^{k}=1$ if i=j and 0 otherwise. Death events consist of

$$\sum_{i=0}^{3}d(x_{i}+1)P(t;x_{0},\ldots ,x_{i}+1,\ldots ,x_{3})-d_{i}x_{i}P(t;x_{0},\ldots ,x_{3}).$$

For i≠j, let $\mu_{i\to j}$ denote the mutation rate into state *j* from state *i* (and 0 if there is no such mutation), then mutation events consist of

$$\begin{array}{rl} & \sum_{i=0}^{3}\sum_{\;j=0}^{3}\mu_{i\to j}(x_{i}+1)P(t;x_{0},\ldots ,x_{i}+1,\ldots ,x_{j}-1,\ldots ,x_{3})\\ & \;-\mu_{i\to j}x_{i}P(t;x_{0},\ldots ,x_{3}). \end{array}$$

The formula for the joint PGF is $G(t;z_{0},\ldots ,z_{3})=E[z_{0}^{N_{S}}z_{1}^{N_{R_{1}}}z_{2}^{N_{R_{2}}}z_{3}^{N_{R_{1,2}}}]$. Eq. D1 can be converted into a PDE involving *G* by applying $\sum_{x_{0},\ldots ,x_{3}}(\cdots )z_{0}^{x_{0}}z_{1}^{x_{1}}z_{2}^{x_{2}}z_{3}^{x_{3}}$:

(D2 ) $$\begin{array}{rl} \frac{dG}{dt} & =\sum_{x_{0},\ldots ,x_{3}}\frac{dP}{dt}(t;x_{0},x_{1},x_{2},x_{1,2})z_{0}^{x_{0}}z_{1}^{x_{1}}z_{2}^{x_{2}}z_{3}^{x_{3}}\\ & =\sum_{x_{0},\ldots ,x_{3}}(\text{birth events}+\text{death events} \end{array}$$

(D3 ) $$\begin{array}{rl} & \;+\text{mutation events})z_{0}^{x_{0}}z_{1}^{x_{1}}z_{2}^{x_{2}}z_{3}^{x_{3}}\\ & =\sum_{i=0}^{3}\left[(b-c(1-\delta_{0,i}^{k}))z_{i}^{2}\frac{\partial G}{\partial z_{i}}-(b-c(1-\delta_{0,i}^{k}))z_{i}\frac{\partial G}{\partial z_{i}}\right]\\ & \;+\sum_{i=0}^{3}\left[d\frac{\partial G}{\partial z_{i}}-dz_{i}\frac{\partial G}{\partial z_{i}}\right]+\sum_{i=0}^{3}\sum_{\;j=0}^{3}\left[\mu_{i,j}z_{j}\frac{\partial G}{\partial z_{i}}-\mu_{i,j}z_{i}\frac{\partial G}{\partial z_{i}}\right]. \end{array}$$

Note $\frac{\partial }{\partial z_{i}}G{|}_{(z_{0},\ldots ,z_{3})=1}=E[X_{i}]$. Further applying $\frac{\partial }{\partial z_{i}}\cdot {|}_{(z_{0},\ldots ,z_{3})=1}$ to Eq. D3 for i∈{0,…,3} yields the same system of mean dynamics as our ODE model (without competition).

#### With competition

In the case with competition the mean process isn’t *exactly* the same as the deterministic ODE process. The difference is attributable to the competition term of $-(b-d)\frac{x_{0}+\cdots +x_{3}}{K}$ or $-(b-c-d)\frac{x_{0}+\cdots +x_{3}}{K}$ present in the effective birth rate. The following term is added to the right-hand-side of Eq. D1:

$$\begin{array}{rl} & \sum_{i=0}^{3}-\frac{b-c(1-\delta_{i,0}^{k})-d}{K}\delta_{0,x_{i}}^{k}\left(\left(-1+\sum_{k=0}^{3}x_{k}\right)(x_{i}-1\right)\\ & \;P(t;x_{0},\ldots ,x_{i}-1,\ldots ,x_{3})-(x_{0}+\cdots +x_{3})x_{i}P(t;x_{0},\ldots ,x_{3})). \end{array}$$

Transforming the system of ODEs into a PDE involving the PGF, and applying $\frac{\partial }{\partial z_{i}}\cdot {|}_{(z_{0},\ldots ,z_{3})=1}$ for i∈{0,…,3}, we obtain the following system:

$$\begin{array}{rl} \frac{dE[N_{S}](t)}{dt} & =(b-d-\delta_{1})E[N_{S}](t)\\ & \;-(b-d)\frac{E[N_{S}(N_{S}+N_{R_{1}}+N_{R_{2}}+N_{R_{1,2}})]}{K}\\ & \;-(\mu_{1}+\mu_{2})E[N_{S}](t),\\ \frac{dE[N_{R_{1}}](t)}{dt} & =(b-c-d)E[N_{R_{1}}](t)\\ & \;-(b-c-d)\frac{E[N_{R_{1}}(N_{S}+N_{R_{1}}+N_{R_{2}}+N_{R_{1,2}})]}{K}\\ & \;-\mu_{2}E[N_{R_{1}}](t)+\mu_{1}E[N_{S}](t),\\ \frac{dE[N_{R_{2}}](t)}{dt} & =(b-c-d-\delta_{1})E[N_{R_{2}}](t)\\ & \;-(b-c-d)\frac{E[N_{R_{2}}(N_{S}+N_{R_{1}}+N_{R_{2}}+N_{R_{1,2}})]}{K}\\ & \;-\mu_{1}E[N_{R_{2}}](t)+\mu_{2}E[N_{S}](t),\\ \frac{dE[N_{R_{1,2}}](t)}{dt} & =(b-c-d)E[N_{R_{1,2}}](t)\\ & \;-(b-c-d)\frac{E[N_{R_{1,2}}(N_{S}+N_{R_{1}}+N_{R_{2}}+N_{R_{1,2}})]}{K}\\ & \;+\mu_{1}E[N_{R_{2}}](t)+\mu_{2}E[N_{R_{1}}](t). \end{array}$$

This almost follows the dynamics of the ODE model, except the competition term involves second and cross moments rather than first moments. For example, the competition term for the first differential equation should be $E[N_{S}](E[N_{S}+N_{R_{1}}+N_{R_{2}}+N_{R_{1,2}}])$ for $E[N_{S}]$ and S(t) to correspond. Note $\text{CoV}[N_{S},N_{S}]=V[N_{S}]=E[N_{S}^{2}]-E[N_{S}{]}^{2}$ and $\text{CoV}[N_{S},N_{R_{1}}]=E[N_{S}N_{R_{1}}]-E[N_{S}]E[N_{R_{1}}]$. Hence, for example:

$$\begin{array}{rl} & \frac{dE[N_{S}](t)}{dt}=\left((b-d-\delta_{1}\right)\\ & \;-(b-d)\frac{E[N_{S}+N_{R_{1}}+N_{R_{2}}+N_{R_{1,2}}]}{K}-(\mu_{1}+\mu_{2})\\ & \;-(b-d)\frac{\text{CoV}[N_{S},N_{S}+N_{R_{1}}+N_{R_{2}}+N_{R_{1,2}}]}{E[N_{S}]K})E[N_{S}](t). \end{array}$$

Similar equalities hold for the other moments. If the last term remains negligible compared to the growth parameter, then the mean dynamics and ODE dynamics should match. This seems to be the case for the relevant populations that supply the rescue mutants (the $R_{2}$ cells in $E_{1}$, the $R_{1}$ cells in both environments, and the *S* cells in $E_{2}$) (Supplementary Fig. A.9). Namely, all terms $\text{CoV}[X,N_{S}+N_{R_{1}}+N_{R_{2}}+N_{R_{1,2}}]/(E[X]K)$ for $X\in \{R_{2},R_{1},S\}$ are much smaller than unity (all such terms remain below ∼0.05).

Significant deviations between the rescuing population and its ODE counterpart occur when there is a non-negligible chance of extinction, for example from applying two rounds of therapy (Supplementary Fig. A.9a,c). Hence in general, for *n* drug sequential therapy, if there is a non-negligible chance of extinction during $E_{k}$ (the *k*th stressful environment induced by drug *k*) for k<n, more work needs to be done to obtain a correspondence between stochastic and the ODE dynamics.

### Expected number of rescue mutants relates to RM(τ)

Let $N_{\mathrm{RM}}$ denote the number of rescue mutants generated throughout a stochastic trace. We are interested in its expectation and whether it equals RM (the number of rescue mutants calculated from the ODE mode, see Eq. A6). We consider the $R_{1}$ cell contribution towards $R_{1,2}$ rescue mutants. Denote $N_{R_{1}},N_{L}$ as the stochastic number of $R_{1}$ cells and rescue lineages arising from mutation from $R_{1}$.

We also make a simplification. Namely that the population supplying the mutants, here $R_{1}$, can be approximated ignoring its outgoing mutation rate. This works so long as the outgoing mutation rate is much smaller than the birth and death rate parameters. In this way, we can consider stochastic mutations into the $R_{1,2}$ population from the $R_{1}$ population within a single $N_{R_{1}}$ trajectory.

Also, for a sufficiently large initial population, *τ*, the striking time, is roughly deterministic and is approximately the time when the mean total population reaches the striking size.

As $N_{R_{1}}(\omega ,t)=N_{R_{1}}$ is a stochastic process, it is a function over both the sample space and time. Consider the *σ*-algebra G generated by all trajectories of $N_{R_{1}}$. For any sample point *ω* within a trajectory, $N_{R_{1}}(\omega ,\cdot )$ is a deterministic function of time, and hence the deterministic infinitesimal rate of accumulation of $R_{1,2}$ rescue lineages at this *ω* is $\pi_{e}\mu_{2}N_{R_{1}}(\omega ,t)$.

Thus, the conditional expectation becomes

$$E[N_{L}|G](\omega )=\int_{0}^{\tau }\pi_{e}\mu_{2}N_{R_{1}}(\omega ,t)\;dt.$$

Apply iterated conditioning and Fubini’s theorem:

$$E[N_{L}]=E[E[N_{L}|G]]=\int_{0}^{\tau }\pi_{e}\mu_{2}E[N_{R_{1}}](t)\;dt.$$

For the SGV $R_{2}$ cells, notice that obviously $E[\pi_{e}N_{R_{2}}(\tau )]=\pi_{e}E[N_{R_{2}}(\tau )]$. Hence combining everything together:

$$\begin{array}{rl} E[N_{\mathrm{RM}}] & =\pi_{e}E[N_{R_{2}}]\\ & \;+\pi_{e}\left(\int_{0}^{\tau }\mu_{2}E[N_{R_{1}}](t)\;dt+\int_{0}^{\tau }\mu_{1}E[N_{R_{2}}](t)\;dt\right)\\ & \;+\pi_{e}\left(\int_{0}^{\tau }\mu_{2}E[N_{S}](t)\;dt+\int_{0}^{\tau }\mu_{2}E[N_{R_{1}}](t)\;dt\right). \end{array}$$

That is to say that $E[N_{\mathrm{RM}}]=\text{RM}(\tau )$ assuming a correspondence between the mean dynamics of the stochastic system and the ODE model (covered in the previous section). This is empirically shown for different parameter values and striking sizes (Supplementary Fig. A.10).

Recall that there is a discrepancy between the analytical formula for the extinction probability and the simulation results at low costs of resistance (Fig. 4). Even though RM(τ) is still the mean number of rescue mutants, at low costs of resistance the distribution of the number of rescue mutants is not necessarily Poisson (which is shown in the below section).

### Distribution of preexisting established $R_{2}$ cells

To investigate the distribution of $R_{2}$ cells analytically, consider the stochastic master equation formulation of the Probability Mass Function (PMF) for the number of $R_{2}$ cells ($N_{R_{2}}$) during treatment 1:

(D4 ) $$\begin{array}{rl} \frac{dP_{N_{R_{2}}}(n;t)}{dt} & =(1-\delta_{0,n}^{k})b_{R}^{\mathrm{eff}}(t)(n-1)P_{N_{R_{2}}}(n-1;t)\\ & \;+d_{R}^{\mathrm{eff}}(t)(n+1)P_{N_{R_{2}}}(n+1;t)\\ & \;-(b_{R}^{\mathrm{eff}}(t)+d_{R}^{\mathrm{eff}}(t))nP_{N_{R_{2}}}(n;t)\\ & \;+(1-\delta_{0,n}^{k})\mu_{2}S(t)P_{N_{R_{2}}}(n-1;t)-\mu_{2}S(t)P_{N_{R_{2}}}(n;t), \end{array}$$

where the *S* population is approximated as a deterministic function of time and with the effective birth and death rates are as in Eq. G6 and Eq. G7, except with the quantity *N* replaced by the deterministic S(t). This represents a birth-death-mutation process with time-inhomogeneous event rates. Mutations from $R_{2}$ to $R_{1,2}$ are ignored. Following Getz (1975), (D4) can be converted to a single partial differential equation involving the probability generating function ($G_{N_{R_{2}}}(z;t)=E[z^{R_{2}}]$):

(D5 ) $$\begin{array}{rl} \frac{\partial G_{N_{R_{2}}}(z;t)}{\partial t} & =(b_{R}^{\mathrm{eff}}(t)z-d_{R}^{\mathrm{eff}}(t))(z-1)\frac{\partial G_{N_{R_{2}}}(z;t)}{\partial z}\\ & \;+\mu_{2}S(t)(z-1)G_{N_{R_{2}}}(z;t). \end{array}$$

Equation (D5) can be solved using the method of the characteristics (Getz 1975). In particular, denoting

$$\begin{array}{rl} \;p(t) & =\exp\;\left(\int_{0}^{t}b_{R}^{\mathrm{eff}}(\tau )-d_{R}^{\mathrm{eff}}(\tau )\;d\tau \right)\\ k(z,t) & =\frac{1}{p(t)(z-1)}-\int_{0}^{t}b_{R}^{\mathrm{eff}}(\tau )[p(\tau ){]}^{-1}\;d\tau \end{array}$$

and

$$A(\tau )\;:\;=\frac{\mu_{2}S(\tau )[p(\tau ){]}^{-1}}{k(z,t)+\int_{0}^{\tau }b_{R}^{\mathrm{eff}}(\tau^{\prime })[p(\tau^{\prime }){]}^{-1}\;d\tau^{\prime }}$$

the PGF can be written as

(D6 ) $$\begin{array}{rl} & G_{N_{R_{2}}}(z;t)\\ & \;=\exp\;\left(\int_{0}^{t}A(\tau )\;d\tau \right)\cdot {\left(1+\frac{1}{k(z,t)}\right)}^{R_{2}(0)} \end{array}$$

(D7 ) $$\;\approx \exp\;\left(\int_{0}^{t}A(\tau )\;d\tau +\frac{R_{2}(0)}{k(z,t)}\right),$$

with approximation (D7) holding for k(z,t) large. There are two $R_{2}$ subpopulations: one that descends from the initial $R_{2}(0)$ population and another that derives from subsequent mutations. The random variables representing these subpopulations are roughly independent at all times as the total $R_{2}$ population contributes little to competition. Therefore in (D6), $(1+\frac{1}{k(z,t)}{)}^{R_{2}(0)}$ represents the PGF of the first $R_{2}$ subpopulation and the exponential term represents the PGF of the second subpopulation.

To obtain the probability of extinction of all $R_{2}$ cells that arise before switching to the second treatment (SGV or standing genetic variation), let $N_{R_{2}}^{e}$ denote the number of $R_{2}$ cells existing at the time of switching that eventually get established. Since $N_{R_{2}}^{e}|N_{R_{2}}∼\text{Binomial}(N_{R_{2}},\pi_{E})$:

$$\begin{array}{rl} G_{N_{R_{2}}^{e}}(z;t) & =E\left[z^{N_{R_{2}}^{e}}\right]=E\left[E\left[z^{N_{R_{2}}^{e}}|N_{R_{2}}\right]\right]\\ & =E\left[{\left((1-\pi_{e})+\pi_{e}z\right)}^{N_{R_{2}}}\right]=G_{N_{R_{2}}}(1-\pi_{e}+\pi_{e}z;t). \end{array}$$

The probability of extinction from $R_{2}$ cells arising before switching is just the mass of $P(N_{R_{2}}^{e}=0)=G_{N_{R_{2}}^{e}}(0;t)$. In the limit where the term $\int_{0}^{t}b_{R}^{\mathrm{eff}}(\tau )[p(t){]}^{-1}\;d\tau$ is negligible, which occurs when $b_{R}^{\mathrm{eff}}(t)$ is made small at fixed $g_{R}^{\mathrm{eff}}(t)$ ($(b-c)/(d+\delta_{1})\ll 1$) and using approximation (D7), we get

(D8 ) $$\begin{array}{rl} & P_{E}^{\mathrm{SVG},R_{2}}(t)\\ & \;\approx \exp\;\left(-\pi_{e}\left(R_{2}(0)p(t)+p(t)\int_{0}^{t}\mu_{2}S(\tau )[p(\tau ){]}^{-1}\;d\tau \right)\right)\\ & \;\approx \exp\;\left(-\pi_{e}R_{2}(t)\right). \end{array}$$

Expression (D8) is what we use in (C1). However when $b_{R}^{\mathrm{eff}}(t)$ is made large at fixed $g_{R}^{\mathrm{eff}}(t)$ (or $(b-c)/(d+\delta_{1})\approx 1$), $P_{E}^{\mathrm{SVG},R_{2}}(t)=G_{N_{R_{2}}}(1-\pi_{e};t)$ can be made arbitrarily close to 1. Intuitively, as the intrinsic birth rate is made more comparable to the intrinsic death rate at some growth rate, the $R_{2}$ population follows a more heavy-tailed distribution than what a corresponding Poisson approximation would suggest. This leaves more mass at the $N_{R_{2}}^{e}=0$ absorbing barrier, and hence a higher probability of extinction.

Consequently, the probability of extinction of the SGV $R_{2}$ population (that exists before the second treatment) calculated by our analytical model (Eq. 1) is expected to be an *underestimate* of the probability of extinction of this population observed in our stochastic simulations especially when birth and death rates are comparable (Supplementary Fig. A.11). For example, we observe this discrepancy in simulations with a zero cost of resistance and low treatment efficacy (Fig. 4).

Another condition in which the breakdown occurs is when we have a large turnover with constant growth rates. This is because b−c is comparable to $d+\delta_{1}$ when we have a large b+d (turnover) with constant *g*. The intuition for this is that the variance of the $R_{2}$ population size increases with turnover. Consider an $R_{2}$ cell during treatment 1. Fixing $b-d-\delta_{1}<0$, the probability that this cell dies before dividing goes to 100% in the limit b=0 (minimal turnover) to just over 50% in the limit b→+∞ (maximal turnover). The latter case generates much more variance in the $R_{2}$ population. But for a given expected $R_{2}$ population, the probability that $R_{2}(\tau )=0$ is larger when the variance is large. Thus, higher turnover leads to greater variance (see also Appendix E: Derivation of the establishment probability), which in turns leads to a higher probability of there being no SGV rescue mutants and a higher probability of extinction.

### Distribution of established lineages of $R_{1,2}$ cells and $R_{2}$ cells

For the other terms in the probability of extinction, consider the dynamics of the established lineages that derive from $R_{1,2}$ rescue mutants during $E_{1}$ and $R_{1,2}/R_{2}$ rescue mutants during $E_{2}$. These lineages come about by mutation at rate $\pi_{e}\cdot r(t)$. Here, r(t) is the rate of formation of the relevant pre-rescue mutant: $\mu_{1}\cdot R_{2}(t)+\mu_{2}\cdot R_{1}(t)$ for $R_{1,2}$ and $\mu_{2}\cdot S(t)$ for $R_{2}$ cells. Without loss of generality, we focus on the *de-novo* $R_{2}$ lineages. Using *deterministic* dynamics in defining the rate r(t) is approximate, and it should hold as long as the probability of extinction during $E_{1}$ is negligible and the mutation rates $\mu_{i}$ are small. Denoting as *L* the random variable counting the number of established lineages:

$$\frac{dP_{L}(n;t)}{dt}=(1-\delta_{0,n}^{k})\pi_{e}\cdot r(t)P_{L}(n-1;t)-\pi_{e}\cdot r(t)P_{L}(n;t).$$

Solving for the PGF of *L* (Getz 1975):

$$G_{L}(z;t)=\exp\;\left((z-1)\int_{0}^{t}\pi_{e}r(\tau )\;d\tau \right).$$

So, *L* follows a Poisson distribution with the expected rate.

## Appendix E: Derivation of the establishment probability

Here we provide the derivation of the establishment probability of a single lineage of resistant cells. This result is taken from Lambert (2006). As described in Appendix B: Using results from evolutionary rescue theory, the establishment probability is the probability with which lineages with positive fitnesses escape stochastic extinction and establish themselves in the population. This leads to evolutionary rescue and thus, founders of lineages with positive fitness that get established are called rescue mutants.

We use the Continuous-time, real-valued Branching (CB) process to model the population dynamics of the resistant lineages. We chose this process because CB processes are the only diffusion processes that satisfy the additive property of the well-known Bienamé-Galton-Watson (BGW) processes, which are commonly used to model stochastic population dynamics. Therefore, a CB process can be used to model the total population size summed over several lineages evolving independently. In our case, we use a CB process which is a continuous function of time, also called branching diffusion. As described in Lamperti (1967), branching diffusions are strong solutions of stochastic differential equations (SDEs) of the form:

(E1 ) $$dZ_{t}=rZ_{t}dt+\sqrt{\sigma Z_{t}}dB_{t},$$

where *B* is the standard Brownian motion, r∈R is the intrinsic growth rate, and $\sigma \in R^{+}$ is the reproduction variance, defined by Martin et al. (2013) as

(E2 ) $$r=\underset{\Delta t\to 0}{\lim}\frac{E\left(\Delta Z_{t}|Z_{t}\right)}{Z_{t}\Delta_{t}}$$

(E3 ) $$r=\underset{\Delta t\to 0}{\lim}\frac{\mathrm{Var}\left(\Delta Z_{t}|Z_{t}\right)}{Z_{t}\Delta_{t}}.$$

We use a general result from diffusion theory that the probability $u(z_{0})$ of the diffusion hitting an absorbing barrier $z_{\mathrm{abs}}$, where $z_{0}$ is the initial condition ($Z_{0}=z_{0}$) is such that the function *u* solves the equation Gu(z)=0 subject to appropriate boundary conditions. Here, *G* is the infinitesimal generator of the diffusion and characterizes the behavior of the diffusion at small time intervals. For our one-dimensional diffusion (Equation E1), *G* is of the form,

(E4 ) $$Gf(z)=rf^{\prime }(z)+\frac{\sigma }{2}f^{\prime \prime }(z).$$

For the absorbing barrier $z_{\mathrm{abs}}=0$, we will obtain the extinction probability of a resistant population starting with $z_{0}$ cells by solving $Gu(z_{0})=0$, with boundary conditions $u(z_{\mathrm{abs}})=1$ and u(∞)=0. The solution of this differential equation is,

(E5 ) $$u(z_{0})=\exp\;\left[\frac{-2rz_{0}}{\sigma }\right].$$

As explained in Martin et al. (2013), the reproduction variance for a simple birth-death process with the birth rate *b* and death rate *d* can be approximated by b+d (turnover). Consequently, we obtain the following expression for the establishment probability, defined as one minus the extinction probability, for a potential rescue lineage starting from a single cell.

(E6 ) $$\pi_{e}=1-\exp\;\left[\frac{-2(b-d)}{b+d}\right].$$

With a cost of resistance, we get,

(E7 ) $$\pi_{e}=1-\exp\;\left[\frac{-2(b-c-d)}{b-c+d}\right].$$

We use this result to compute the extinction probabilities with our analytical model in Methods.

We can compare our establishment probability (Eq. E7) with a well-known result for the birth-death process:

(E8 ) $$\pi_{e}^{A}=1-\frac{d}{b}.$$

With a cost of resistance, *b* in (E8) should be replaced with b−c. This expression is consistent with the method of simulation we use since the Gillespie simulation is a numerical solution of the master stochastic equation corresponding to the linear birth-death process. The difference between the extinction probabilities obtained using (E7) and (E8) is negligible. To see this, firstly note that the first order Taylor approximations of $\pi_{e}(b,d)$ and $\pi_{e}^{A}(b,d)$ centered about $\left(q_{0},q_{0}\right)$ are $(b-d)/q_{0}$ and so behave quite similarly for small g=b−d. Their values are made most different as (b,d)→(∞,d) for any fixed *d* and in this limit become $\pi_{e}\to 1-e^{-2}$ and $\pi_{e}^{A}\to 1$. These values differ by ≈0.13, which is quite large, but the more relevant difference is between expressions of the form $e^{-\pi_{e}z}$ and $e^{-\pi_{e}^{A}z}$, where *z* is the number of potential rescue mutants (preexisting or newly generated). As (b,d)→(∞,d), the maximum difference is around 0.05 and is obtained at z≈1.07. Finally, any qualitative comparisons between probabilities of extinction remain the same independent of choice of $\pi_{e}$ or $\pi_{e}^{A}$.

## Appendix F: Comparison with combination therapy

To illustrate why combination therapy will not necessarily outperform a two-strike strategy, consider the case where the total dose is limited by toxicity. Let *u* represent the proportion of the drug cocktail that is drug 1. Suppose that drugs 1 and 2 are strictly additive in both efficacy and toxicity. Our model is then as follows (without competition):

$$\begin{array}{rl} & \dot{S}=(\gamma_{S}-\delta_{1}u-\delta_{2}(1-u))S,\\ & {\dot{R}}_{1}=(\gamma_{1}-\delta_{2}(1-u))R_{1}+\mu_{1}S,\\ & {\dot{R}}_{2}=(\gamma_{2}-\delta_{1}u)R_{2}+\mu_{2}S,\\ & {\dot{R}}_{1,2}=g_{1,2}R_{1,2}+\mu_{2}R_{1}+\mu_{1}R_{2}. \end{array}$$

Now suppose that $\delta_{2}>\gamma_{1}>\delta_{2}/2$ and $\delta_{1}>\gamma_{2}>\delta_{1}/2$. If we opt for combination therapy then for any 0≤u≤1 we must have 1−u≤1/2 or u≤1/2, or both. Therefore $\delta_{2}(1-u)\leq \delta_{2}/2<\gamma_{1}$ or $\delta_{1}u\leq \delta_{1}/2<\gamma_{2}$, or both. It follows that $R_{1}$ and $R_{2}$ cannot both be contained (explicitly, in the stochastic formulation of the model, at least one of them will likely increase indefinitely). On the other hand, because $\delta_{2}>\gamma_{1}$ and $\delta_{1}>\gamma_{2}$, two-strike therapy might succeed in eliminating the tumor. Hence, there are some settings in which two-strike therapy can be better.

## Appendix G: Stochastic simulation model

The total population at time *t* is $N(t)=S(t)+R_{1}(t)+R_{2}(t)+R_{1,2}(t)$. The code developed for the implementation of this model is designed to be highly versatile and easy to modify.

### Gillespie implementation

The stochastic simulation algorithm (SSA), commonly referred to as the Gillespie algorithm (Gillespie 1977), is a Monte Carlo method initially devised in 1977 for modeling the temporal evolution of chemical reactions in a well-mixed system. We implement the Gillespie algorithm to simulate the population dynamics of our system in the context of two-strike therapy. The idea is that given a set of rates corresponding to birth, death and mutation events, we can track the size of each subpopulation. These rates are specified for all cell types and can change with time or in response to the environment. The basic steps of our algorithm are as follows:

1. Initialize the system by specifying an initial population for all cell types (S(0), $R_{1}(0)$, $R_{2}(0)$, $R_{1,2}(0)$) and setting the time to zero. Define all possible demographic events (birth, death, mutation) and their corresponding rates.
2. Compute the rate of any one event occurring, which is the sum of all individual rates. Individual event rates are denoted by $\omega_{i}(t)$ for each event *i*. Then, obtain the time interval after which the next event will take place. To do this, generate a sample from an exponential random variable with the rate parameter equal to the total rate $\sum_{i}\omega_{i}(t)$. Alternatively, one can generate a random number $z_{1}$ from the uniform distribution between 0 and 1 and use the following formula to determine the time interval for the next event: $t_{\mathrm{int}}=-\ln\;(z_{1})/\sum_{i}\omega_{i}(t)$
3. Calculate event probabilities for the next event by dividing the individual event rates by the total rate: $p_{i}(t)=\omega_{i}(t)/\sum_{i}\omega_{i}(t)$. Use these probabilities to select the next event by generating a random number $z_{2}$ between 0 and 1. The chosen event *k* would be the largest *j* such that $z_{2}-\sum_{i=0}^{\;j}p_{i}(t)>0$.
4. Implement the chosen event by updating the population of the corresponding cell types. Increment time $t=t+t_{\mathrm{int}}$. For example, if the chosen event is the birth of an $R_{2}$ cell, then $R_{2}$$(t+t_{\mathrm{int}})=$ $R_{2}$(t)+1.
5. At each time point, record the population size and whether it is before or after the nadir. The value of the population nadir, $N_{\min}$ is known (computed for each set of simulations according to the algorithm described later in this appendix). If in the before(after) nadir regime, the population size is less(greater) than the switching size N(τ) and the first treatment is ongoing, switch to the second treatment. If the second treatment is ongoing, then skip this step.
6. Repeat steps 2–4 till a stopping condition is reached.

Note that the effects of carrying capacity and treatment are included while specifying birth and death rates in the next section. Simulations are stopped under one of three conditions: if the population goes extinct, if it exceeds the maximum simulation time, or if it exceeds the maximum population size, $N_{\max}$. Similarly, the outcome of one run of a simulation can be one of three possibilities: extinction (N(t)=0), progression (N(t)≥N(0)) or persistence (0<N(t)<N(0)). Note that there is not a one-to-one correspondence between stopping conditions and simulation outcome (see Table G2).

### Determining rates of demographic event

Following the Gillespie algorithm, one must define all possible demographic events at the beginning of the simulation and define rates corresponding to those events at each time step. Note that an individual event includes the type of event and the type of cell. For example, a mutation event *S*$S\to R_{1}$ is one individual event and has a rate specified for it. Similarly, the birth of an *S* cell is a different event than the birth of an $R_{1}$ cell. All simulation parameters used to compute the individual event rates are listed in Table 1.

**Table G2.** Stopping conditions and corresponding outcomes of a simulation.

Stopping condition	Outcome condition	Outcome
N(t)=0	N(t)=0	Extinction
t≥T	N(t)≥N(0)	Progression
	0<N(t)<N(0)	Persistence
$N(t)\geq \min(0.99K,N_{\max})$	N(t)≥N(0)	Progression
	0<N(t)<N(0)	Persistence

In the second condition, *T* is the maximum simulation time defined at the beginning of the simulation. If we see a significant number of outcomes with persistence (more than 10%), it means that *T* is not large enough and the simulation is run again with a higher *T* value. In the third condition, the threshold 0.99K is arbitrary. The outcome remains the same as long as the threshold is close to *K*. We take the minimum of two quantities for cases where *K* is much larger than the initial population size N(0), and a threshold of $N_{\max}$ is enough to declare the outcome. In all cases, K≥N(0), and $N_{\max}>N(0)>0$, so persistence in the last condition is not observed. However, the condition is mentioned for the sake of completion. T=500; $N_{\max}=1.5\times 10^{6}$.

We derive the birth and death rates for all cell types from the deterministic logistic model for population growth.

(G1 ) $$\frac{dM(t)}{dt}=g_{M}(t)M(t),$$

where $g_{M}(t)$ is the growth rate of subpopulation $M\in \{S,R_{1},R_{2},R_{1,2}\}$. Since the growth rate of all resistant subpopulations are taken to be equal ($g_{R_{1}}=g_{R_{2}}=g_{R_{1,2}}=g_{R}$), we have,

(G2 ) $$g_{S}(t)=g_{S}\left(1-\frac{N(t)}{K}\right)-\delta_{i};\;i=1,2$$

(G3 ) $$g_{R}(t)=g_{R}\left(1-\frac{N(t)}{K}\right)-\delta_{i};\;i=1,2$$

where the total treatment-induced death rate in environment *i* is $\delta_{i}$, and the presence of this term depends on the sensitivity of different cell types in both environments. For example, the growth rate of $R_{1}$ cells in $E_{1}$ will not include the treatment-induced death term, but in $E_{2}$ will have a $\delta_{2}$ term. Here, to derive the demographic rates used in the stochastic simulations, we consider the case of a cell type susceptible to one treatment but resistant to another.

The intrinsic growth rates of sensitive and resistant subpopulations differ by the cost of resistance. We can write the intrinsic growth rates as the difference between intrinsic birth and death rates like so:

(G4 ) $$g_{S}(t)=(b-d)\left(1-\frac{N(t)}{K}\right)-\delta_{i};\;i=1,2$$

(G5 ) $$g_{R}(t)=(b-c-d)\left(1-\frac{N(t)}{K}\right)-\delta_{i};\;i=1,2$$

where b,d and *c* are the intrinsic birth rate, death rate and the cost of resistance. Now, we split the subpopulation growth rates into “effective” birth and death rates to use for our simulations. This is because the stochastic simulation model requires separate birth and death rates, while the analytical model is parametrized in terms of growth rates of cell lineages ($g_{S}$ and $g_{R}$). Therefore, the effective birth and death rates take into account the effects of competition and treatment while simulating birth and death as separate events.

(G6 ) $$b_{S}^{\mathrm{eff}}(t)=b-(b-d)\left(\frac{N(t)}{K}\right);\;d_{S}^{\mathrm{eff}}(t)=d+\delta_{i}$$

(G7 ) $$b_{R}^{\mathrm{eff}}(t)=(b-c)-(b-c-d)\left(\frac{N(t)}{K}\right);\;d_{R}^{\mathrm{eff}}(t)=d+\delta_{i}.$$

We use Equations G6 and G7 in our simulations. Note that the separation of terms in the last step is not unique. The only constraint is that the separation follows the condition required for the implementation of carrying capacity, i.e. when the total population is equal to *K*, we must have $b_{S}^{\mathrm{eff}}(t)=d_{S}^{\mathrm{eff}}(t)$ and $b_{R}^{\mathrm{eff}}(t)=d_{R}^{\mathrm{eff}}(t)$ (without treatment). We chose a simple way to implement this by taking a density-dependent birth rate and a density-independent death rate. Additionally, it is convenient and intuitive to group the death-rate terms together in $d_{S}^{\mathrm{eff}}(t)$ and $d_{R}^{\mathrm{eff}}(t)$.

For mutation events, we consider the same mutation rate $\mu_{E_{i}}(i=1,2)$ for all cell types, which may change with the environment. Once the source population for an event is chosen (say, *S* cells), the target cell is chosen according to the rates of acquiring each type of mutation. These mutation probabilities from one cell type to the other can be modified.

### Switching to the second treatment

We consider two environments $E_{1}$ and $E_{2}$, each corresponding to the two strikes or treatments. Now we ask the question: in the simulations, how does the environment change with time? In other words, when do we switch treatments and apply the second strike?

We can either switch at a given population size or a given time. For most of our results, we choose to switch at a given size (N(τ)). We run simulations with the same parameter values but with different random seeds, using the algorithm described previously. For a single random seed, we have multiple simulation runs, each with a different switching size N(τ). This constitutes one set of simulations. We do this so that the population trajectory before the second treatment is the same and any difference observed in the extinction probabilities is due to changes in the switching size N(τ). For a set of simulations, the population nadir $N_{\min}$ is the same for all runs.

To produce plots like Fig. 2a, we run simulations with 100 random seeds (100 independent sets of simulations). Effectively, we run 100 independent simulations for a given parameter set and switching size. In the end, we compute extinction probabilities for different values of N(τ) by dividing the number of simulations with an extinction event by the total number of simulation sets (100 by default). The algorithm is as follows:

1. Select a set of parameter values. These parameters are kept constant throughout this set of simulations.
2. Set a random seed for this set of simulations, thus eliminating differences due to stochasticity before switching to the second treatment.
3. Run the first simulation without any second strike. Equivalently, set N(τ) equal to zero for this run. From the results of this run, save the value of $N_{\min}$ and its corresponding time point $t(N_{\min})$.
4. Run the remaining set of simulations with increasing values of N(τ), following the algorithm described previously. See Fig. 1a for an illustration of the switching sizes. Record the outcomes of all the runs according to Table G2.
5. Repeat steps 2–4 for the desired number of random seeds. Each set of simulations with a different random seed is independent and will have different values of $N_{\min}$. However, the values of N(τ) are kept the same for each independent realization in order to calculate extinction probabilities.
6. Compute extinction probabilities for each switching size using outcomes from all sets of simulations (random seeds):
  $$P_{E}(N(\tau ))=\frac{\text{number of simulations ending in extinction}}{\text{total number of simulations}}.$$
  For example, if $P_{E}(5,000)=0.8$, it means that in 80 of the 100 simulation sets (each with a different random seed), we observed population extinction when switching at a population size of 5,000.
