# Supplementary material for: Preventing evolutionary rescue in cancer using two-strike therapy
Source: Genetics. 2025 Nov 26;232(2):iyaf255. doi: 10.1093/genetics/iyaf255 (PMC13270307; doi:10.1093/genetics/iyaf255)
Supplement: iyaf255_Supplementary_Data [file iyaf255_Supplementary_Data.pdf]

Preventing evolutionary rescue in cancer using two-strike therapy: Supplementary Figures

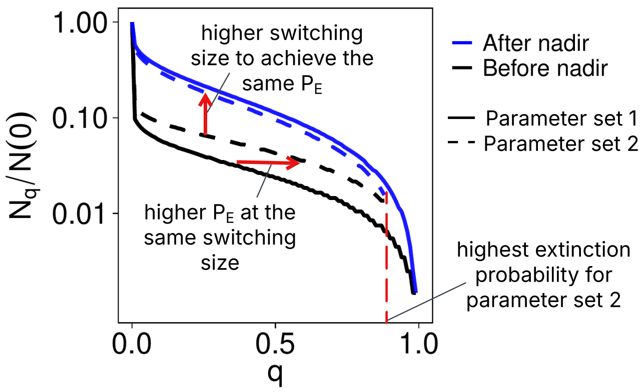

Supplementary Figure A.1 An illustration of why an N_q curve lying above the other implies a better treatment outcome. Here, the dashed curve lies above the solid curve in the before-nadir regime and therefore indicates better treatment outcome in that regime. However, if switching after the nadir, the parameter set corresponding to the solid curve leads to better treatment outcome. This is because the solid curve is higher in the after-nadir regime.

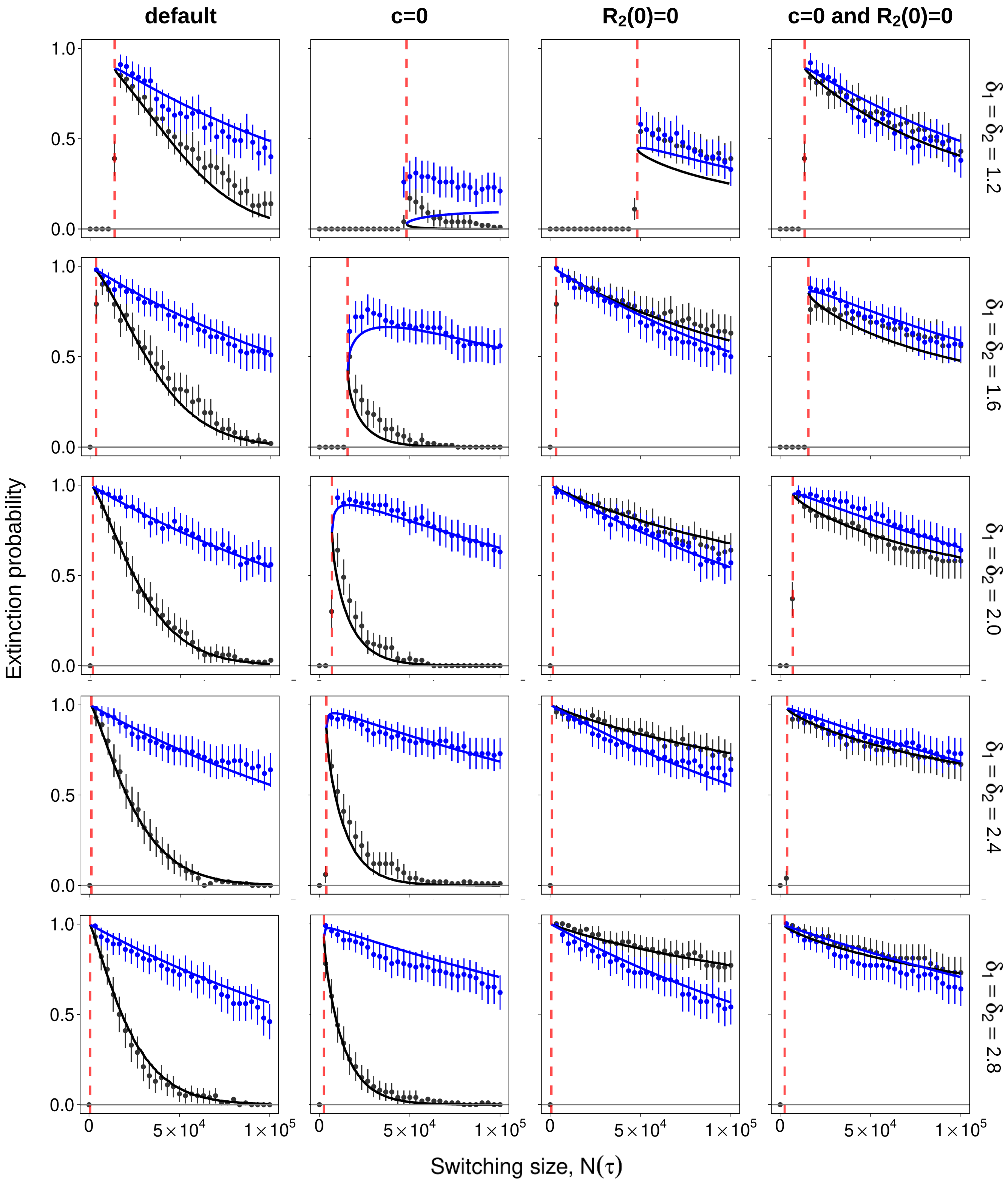

Supplementary Figure A.2 Simulation results for switching sizes before and after $N_{\min}$. Extinction probabilities for different switching sizes (see Appendix G: Stochastic simulation model) are plotted. The black points indicate before nadir switching, and blue points indicate switching after $N_{\min}$. All parameters except the cost of resistance, treatment efficacy and initial R_2 population are set to their default values. Error bars show 95% binomial proportion confidence intervals. Extinction probabilities from the simulations are estimated for each switching size as the proportion of extinction outcomes in 100 independent runs.

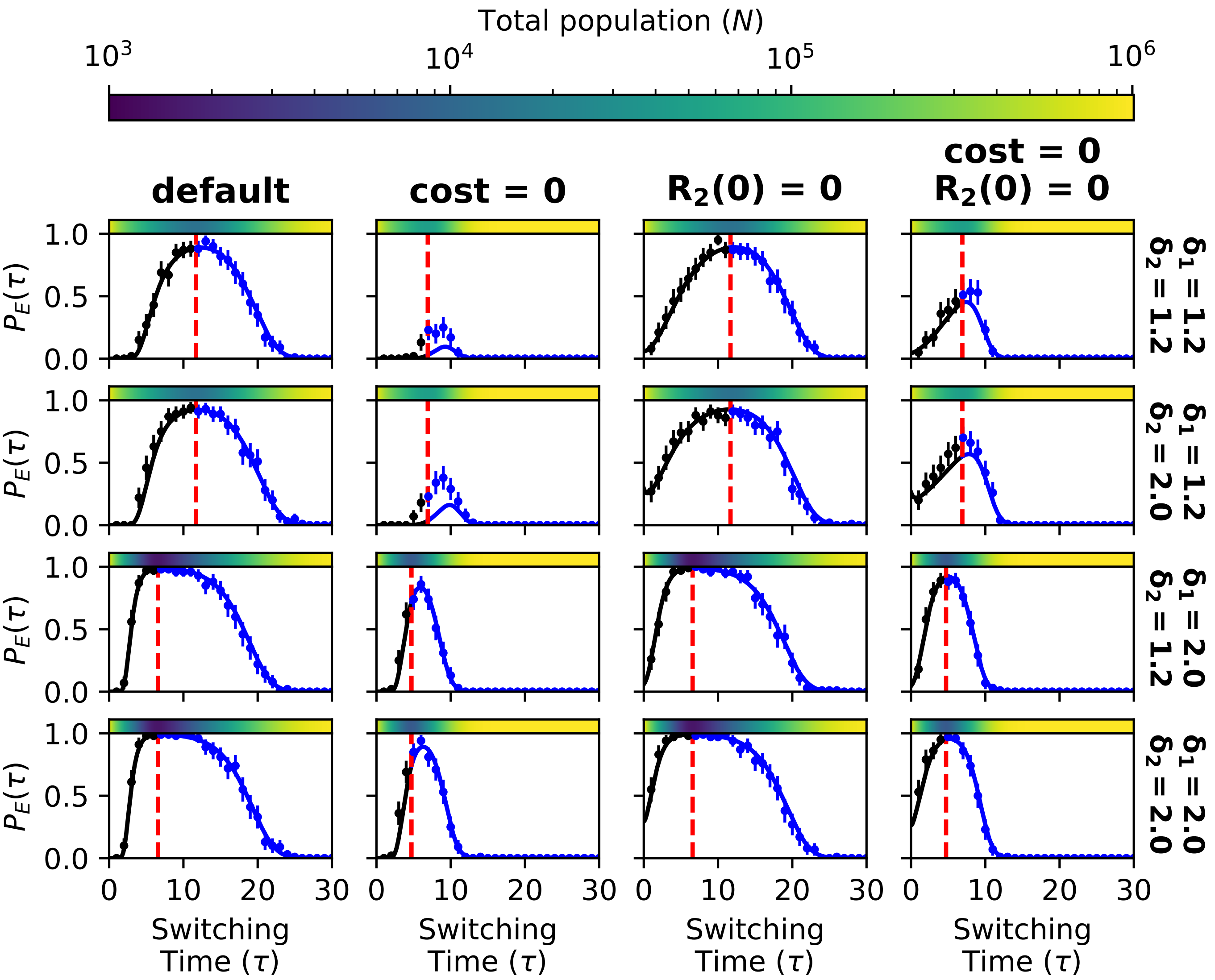

Supplementary Figure A.3 Time windows of high extinction probability. Solid lines are obtained from Equation 1 under deterministic population dynamics with switching to the second drug at time τ. The dots with 95% confidence intervals are from 100-replicate Gillespie runs. Each subplot shows extinction probability trajectories under different modifications of the default parameter values. Population sizes (from the ODE model) are shown in the coloured bar, and the dotted red line shows when the nadir is achieved in the absence of a second strike.

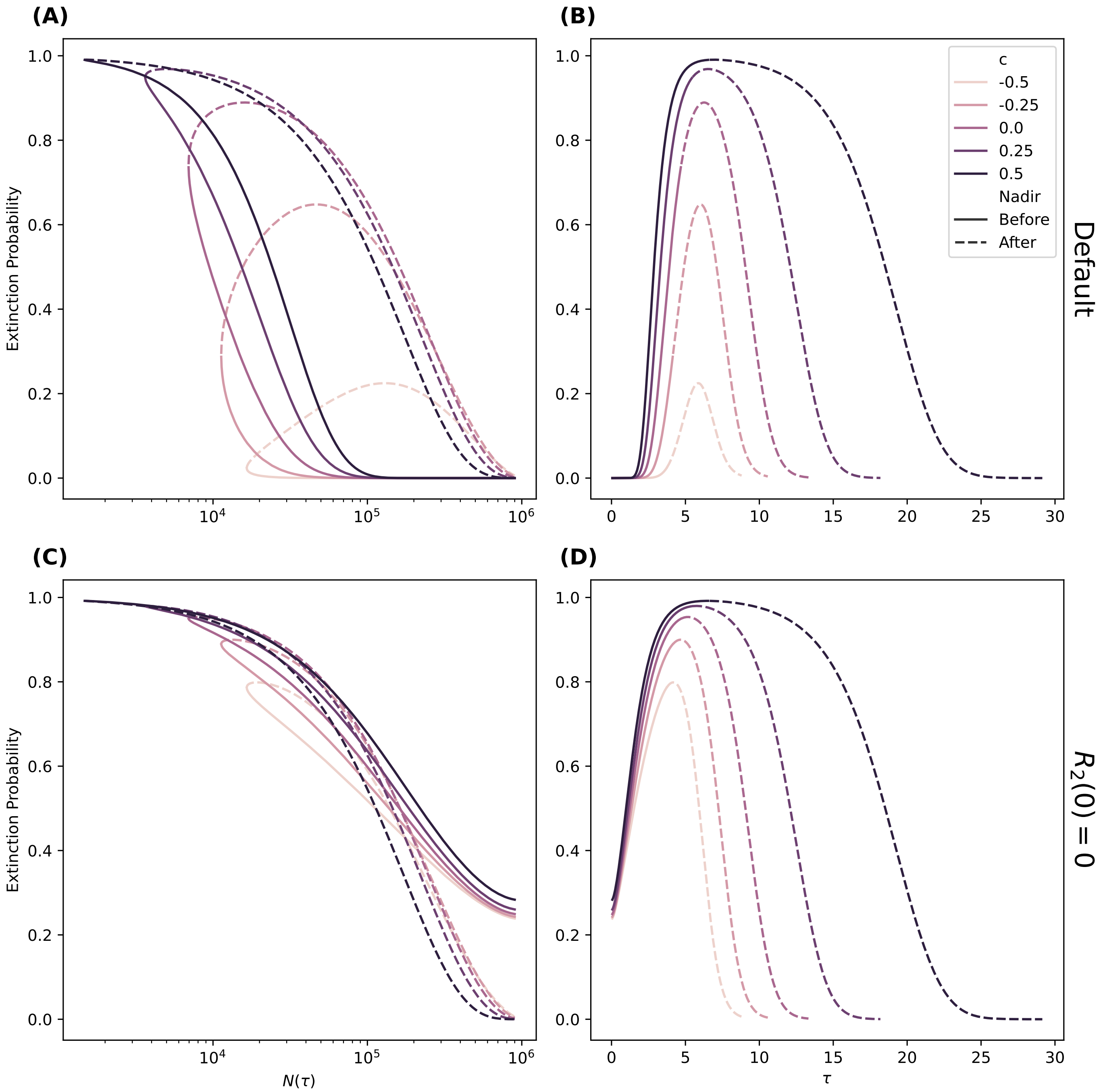

Supplementary Figure A.4 Probability of tumour extinction according to the analytical model with competition for various costs of resistance: for a given switching size **(A,C)** or a given switching time **(B,D)**. The initial R_2 population is set to 0 in the bottom row (C,D). Other parameters have default values (Table 1).

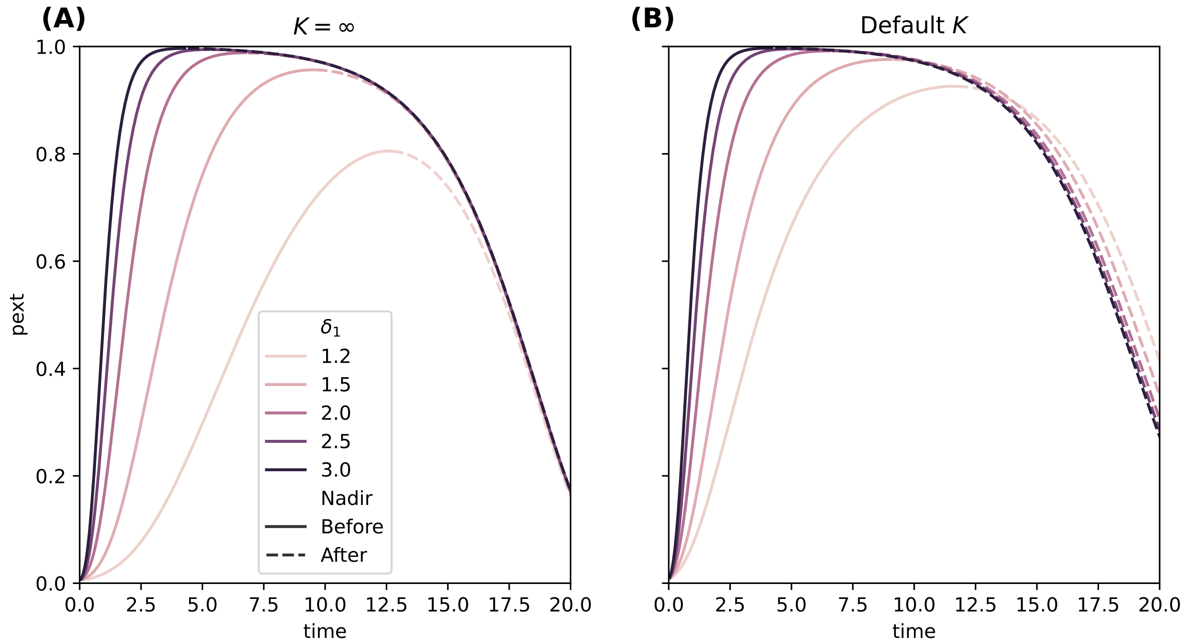

Supplementary Figure A.5 Probability of extinction as a function of switching time according to the analytical model, either without competition **(A)** or with competition **(B)**. Treatment 1 is varied within $\delta_1 \in [1.2, 3]$. Other parameters have default values (Table 1).

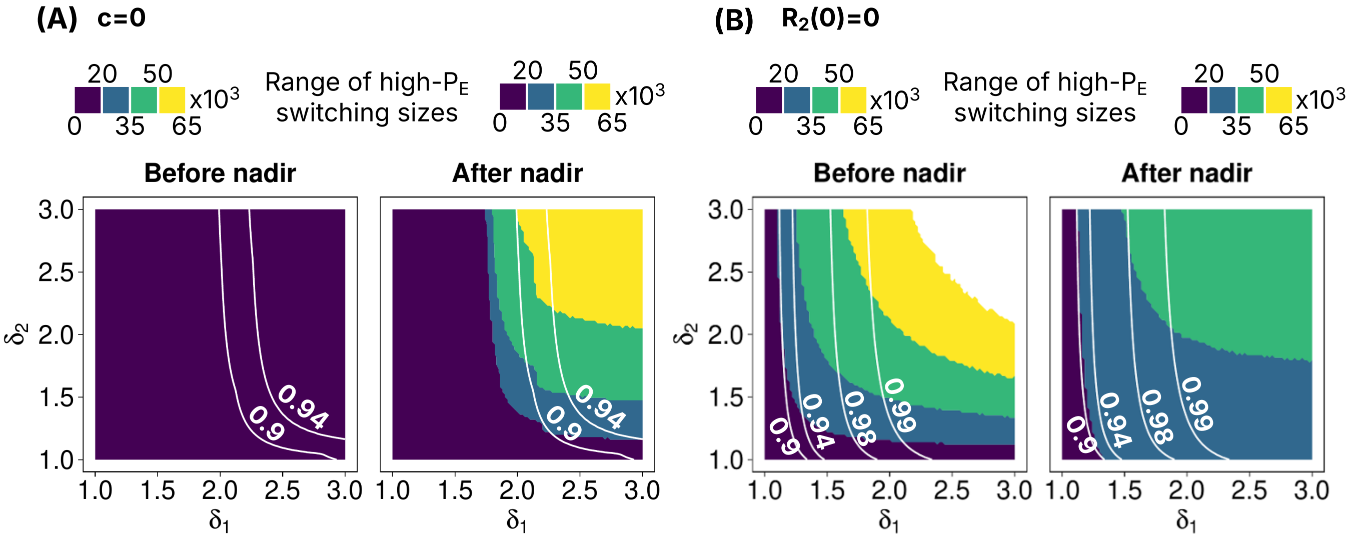

Supplementary Figure A.6 Heatmaps (obtained from the analytical model) showing the range of $N(\tau)$ values that give a high extinction probability (≥ 0.8) for different combinations of treatment efficacies δ_1 and δ_2 in the cases with cost$= 0$ **(A)** and $R_2(0) = 0$ **(B)**. For either case, both before-nadir and after-nadir switching points are considered. White lines indicate optimal extinction probability contours (highest extinction probability across all switching sizes). In the leftmost panel, no high-P_E regions exist.

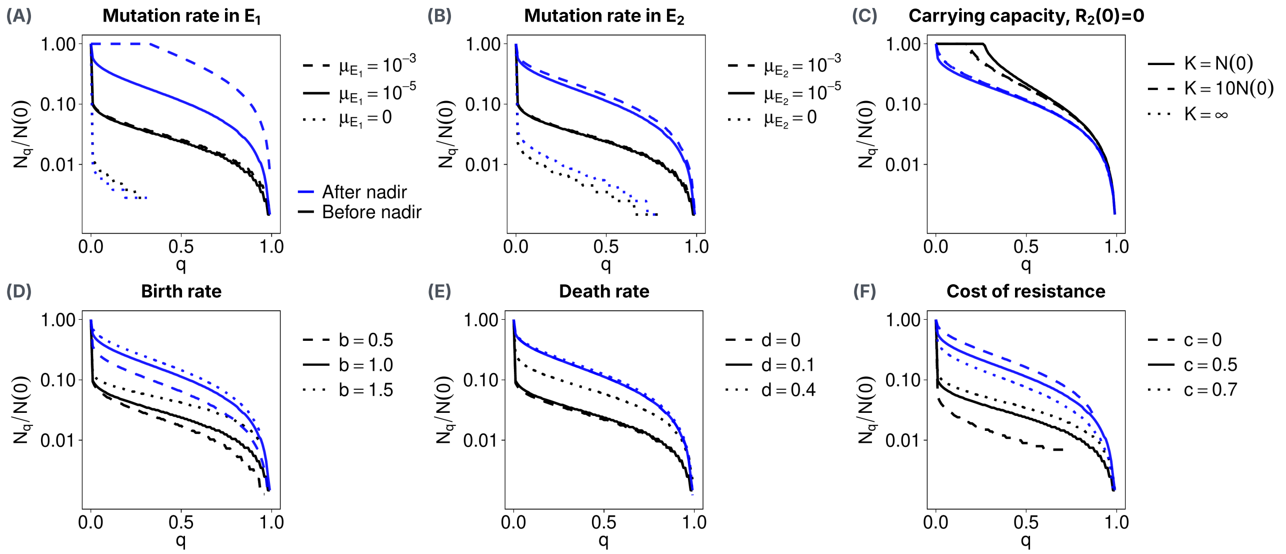

Supplementary Figure A.7 (A,B): Normalised N_q vs q plots for different values of total mutation rates in E_1 (panel A) and E_2 (B). The value of $\mu_{E_1}(\mu_{E_2})$ is kept constant at 10^{-5} when $\mu_{E_2}(\mu_{E_1})$ is varied. Changing the total mutation rate in both environments individually has the same effect (qualitatively). **(C):** Normalised N_q vs q plot for different values of the carrying capacity in the case of $R_2(0) = 0$. **(D,E,F):** Normalised N_q vs q plots for different values of the intrinsic birth rate, death rate and cost of resistance. In all panels, the solid line indicates default parameter values. This figure is obtained using the analytical model only.

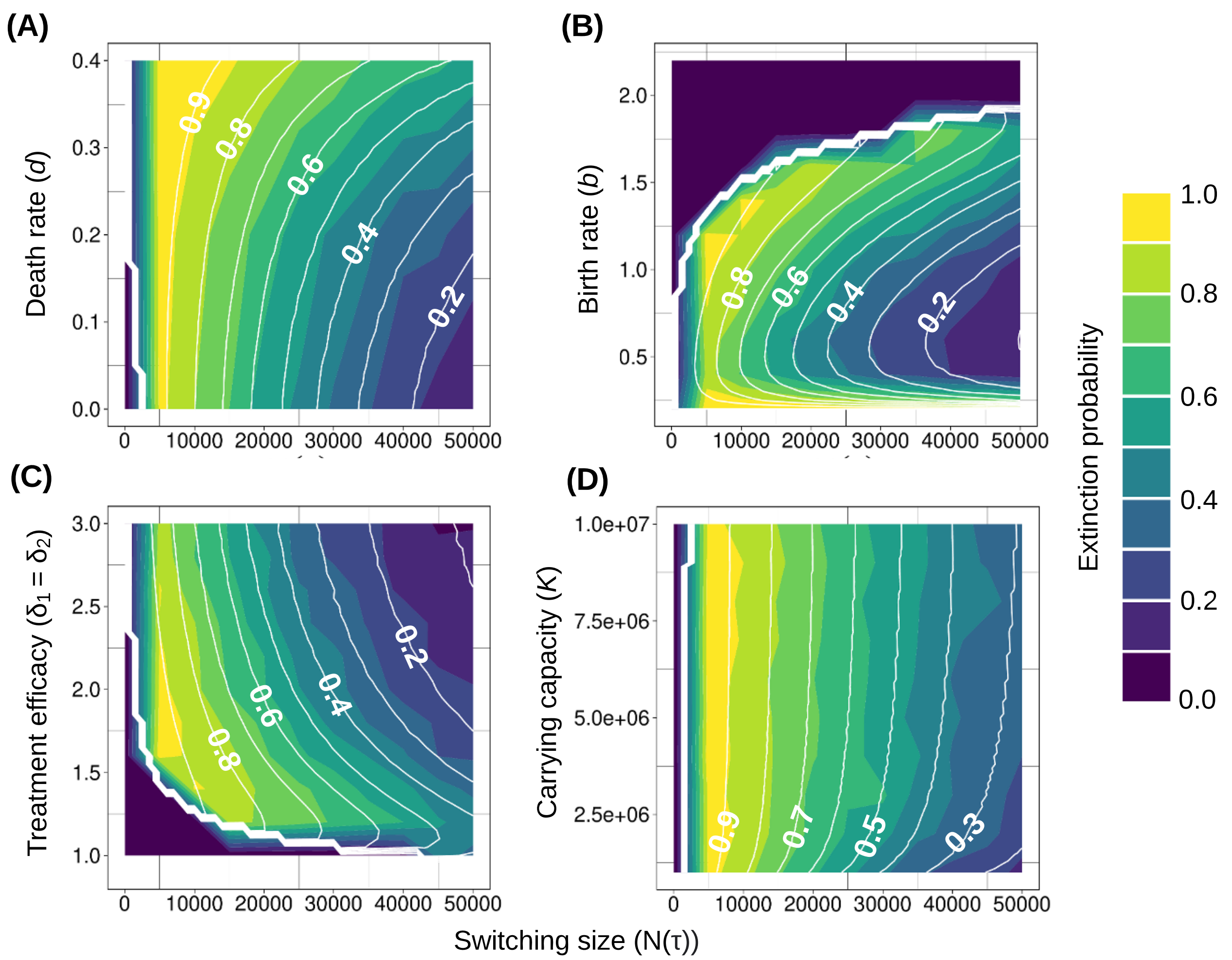

Supplementary Figure A.8 Before-nadir extinction probability heatmaps for varied death rate **(A)**, birth rate **(B)**, treatment efficacy **(C)**, and carrying capacity **(D)**. Solid white contours (with labels) show analytical results. Stochastic simulation results are denoted by the colour scale. Extinction probabilities from the stochastic model are obtained by using the outcomes of 500 simulations with the same parameter values and initial conditions. In panel C, treatment efficacies in both environments are equal (δ_1=δ_2). We do not consider treatment efficacies below 0.9 because that is the intrinsic growth rate of S cells, due to which $\delta <$ 0.9 will only give positive growth rates for all cells in the population. The dark regions in the plots (e.g. top-left region in panel B) have low extinction probabilities because $N(\tau) < N_{\text{min}}$ at those points. Other parameters have default values (Table 1).

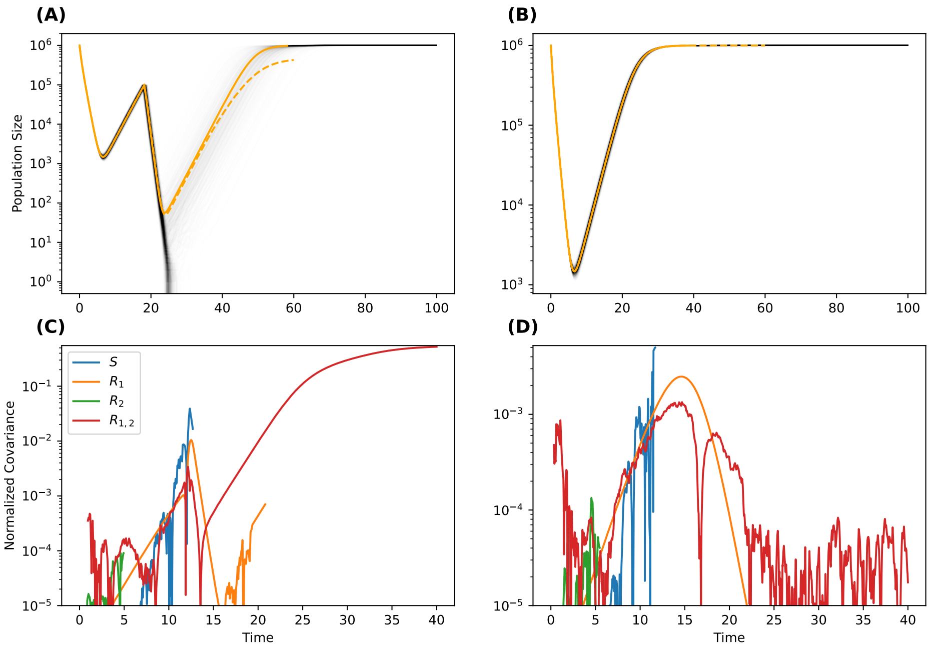

Supplementary Figure A.9 Mean stochastic and ODE dynamics under default parameters with an after nadir strike at $N(\tau) = 10^5$ **(A,C)** or no strike **(B,D)**. For the top row (A,B), the dotted orange line indicates the sample mean from 1,000 Gillespie runs and the solid orange line indicates the total population $N(t)$ obtained from solving the ODE model. Black lines indicate individual stochastic traces. The bottom panel plots sample estimates for $\frac{\mathrm{CoV}[X, N_S + N_{R_1} + N_{R_2} + N_{R_{1,2}}]}{\mathbb{E}[X]K}$ where $X \in \{S, R_1, R_2, R_{1,2}\}$.

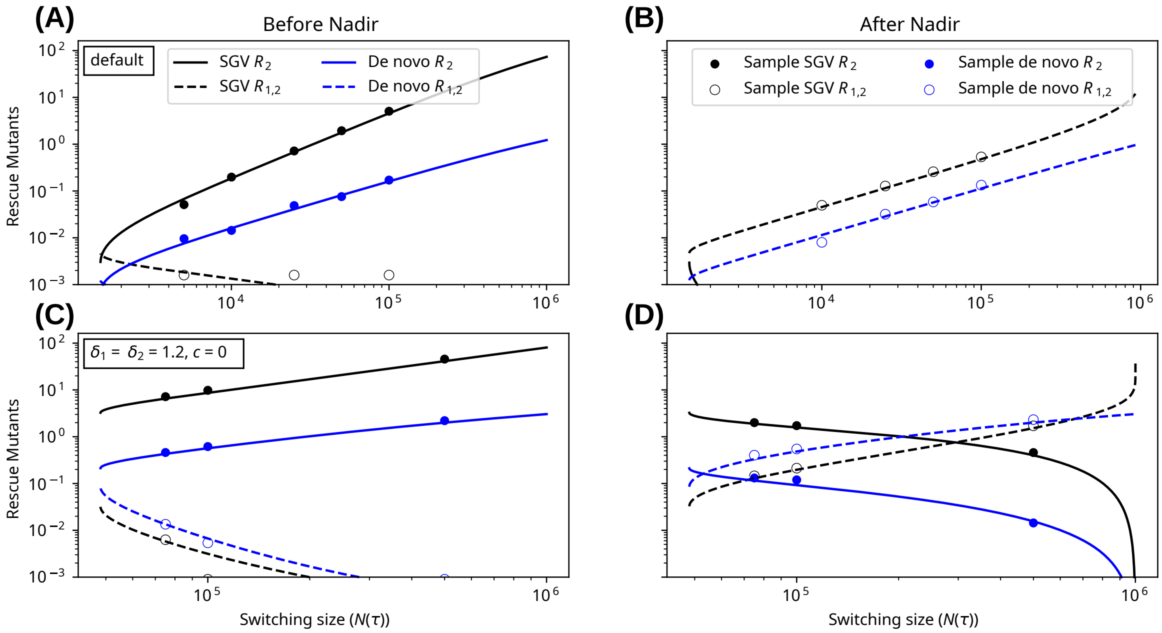

Supplementary Figure A.10 Comparing the sample means for the mean number of rescue mutants generated from simulation (circles) and the calculated number of rescue mutants from the ODE model (see Eq. 7) for varied switching sizes either before the nadir **(A,C)** or after the nadir **(B,D)**. 1000-replicate Gillespie runs are performed for each switching size. The top row (A,B) uses default parameters and the bottom row (C,D) uses $\delta_1 = \delta_2 = 1.2$ and $c = 0$.

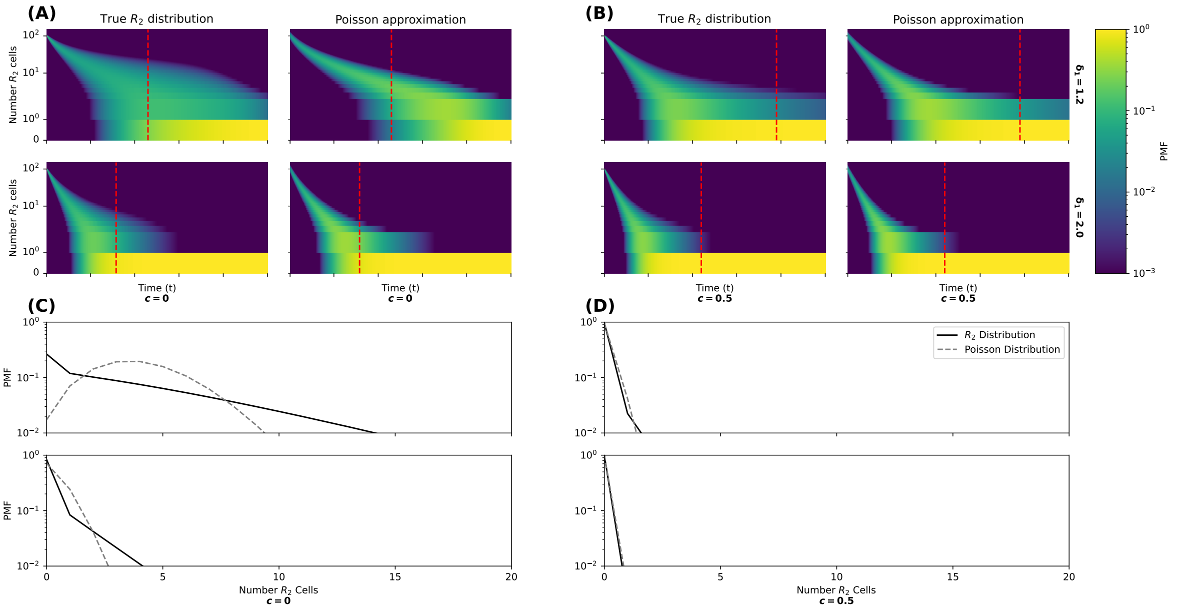

Supplementary Figure A.11 (A,B): Distribution of R_2 cells over time in the absence of a strike when $c = 0$ (panel A) and $c = 0.5$ (B). Distributions are obtained by numerically solving Eq. 33 (left columns of A and B) or by considering a Poisson approximation with rate $\lambda = R_2(t) \approx \mathbb{E}[N_{R_2}]$ (right columns). Results are shown for low treatment efficacy (top row) and high efficacy (bottom row). Other parameters have default values (Table §). The dashed red line indicates when the nadir is achieved. **(C,D):** Distributions at the time the nadir is reached when $c = 0$ (panel C) and $c = 0.5$ (D). Distributions are shown for low treatment efficacy (top row) and high efficacy (bottom row).

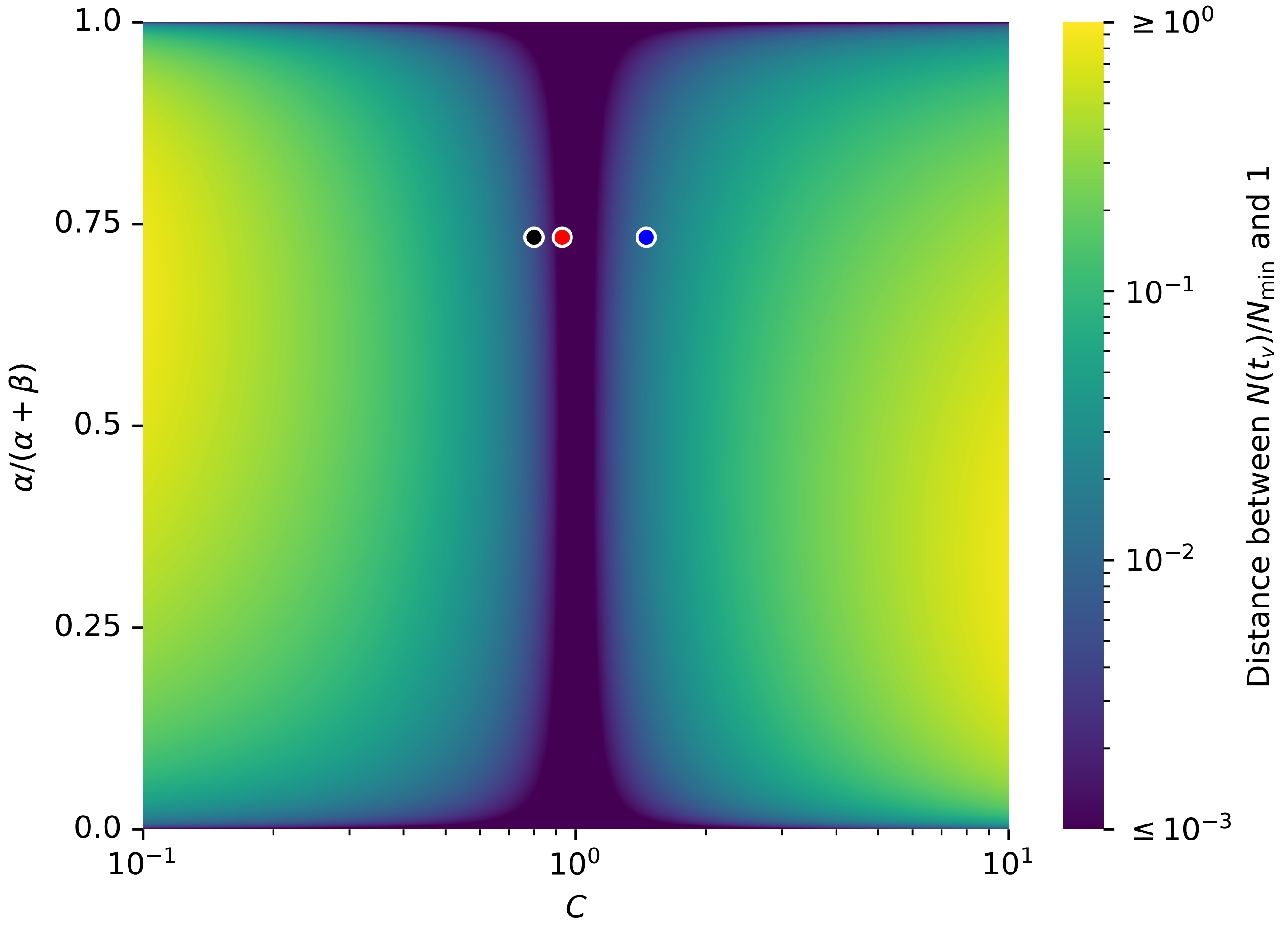

Supplementary Figure A.12 Measuring how close $\frac{N(t_v)}{N_{\min}}$ (calculated using Lemma A1) is to 1, where t_v can stand for the time of minimal standing genetic variants (SGV), time of minimal de-novo variants (DN), or time of optimal probability of extinction (opt). The coefficients α and β were defined in Appendix 1 to be $\delta_1 - \gamma_S$ and γ_1, respectively. Then quantity C on the horizontal axis corresponds to $C = \frac{\gamma_1 \pi_2}{c_2 \pi_{1,2}}$ for $v = \text{SGV}$, $C = \frac{\pi_2/(\delta_2-\gamma_S)}{\pi_{1,2}/(\delta_2-\gamma_1)}$ for $v = \text{DN}$, and $C = \frac{\pi_2\left(\frac{1}{\delta_2-\gamma_S}+\frac{1}{c_2}\right)}{\pi_{1,2}\left(\frac{1}{\delta_2-\gamma_1}+\frac{1}{\gamma_1}\right)}$ for $v = \text{opt}$ (see the sections on optimal switching in Appendix 1. The black, blue, and red dots indicate the default parameter choices for $v = \text{SGV}, \text{DN}, \text{opt}$, respectively.

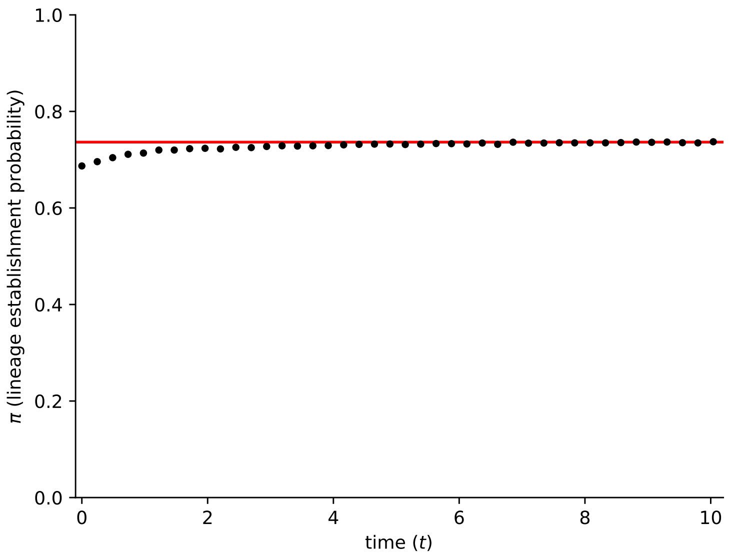

Supplementary Figure A.13 Establishment probability of rescue lineages, starting with 1 cell at different times, assuming competition under just S-cell dynamics. Parameters used are $b = 1.0$, $d = 0.1$, $c = 0.5$, $\delta = 1.2$. The red line represents the theoretical establishment probability assuming no competition. The black dots are generated from numeric solutions to the branching diffusion equation (15) using the Euler-Maruyama scheme with $\Delta t = 10^{-3}$ and 100,000 replicates
