# Supplementary material for: Preventing evolutionary rescue in cancer using two-strike therapy
Source: Genetics. 2025 Nov 26;232(2):iyaf255. doi: 10.1093/genetics/iyaf255 (PMC13270307; doi:10.1093/genetics/iyaf255)
Supplement: iyaf255_Peer_Review_History [file iyaf255_Peer_Review_History.pdf]

GENETICS

Preventing evolutionary rescue in cancer using two-strike therapy

Srishti Patil, Armaan Ahmed, Yannick Viossat, and Robert Noble

NOTE: The reviews and decision letters are unedited and appear as submitted by the reviewers.
In extremely rare instances and as determined by a Senior Editor or the EIC, portions of a review may be redacted. If a review is signed, the reviewer has agreed to no longer remain anonymous.
The review history appears in chronological order.

Review Timeline:

Submission Date:	2024-08-27
Editorial Decision:	2024-11-15
Resubmission Received:	2025-06-30
Editorial Decision:	2025-08-17
Resubmission Received:	2025-09-22
Accepted:	2025-10-24

November 15, 2024

GENETICS-2024-307406
Preventing evolutionary rescue in cancer

Dear Dr. Noble:

Three experts in the field have reviewed your manuscript, and I have read it as well. All find the paper interesting, myself included. All also point to important shortcomings in terms of presentation/structure/explanations. Improving the presentation and improving the explanation of the results (give more intuition to readers) is key for this paper. You will see that the reviewers offer really many and detailed suggestions (many in common). Revising the text represents a significant work, given the numerous places where it can be improved, and given that the structure of the text can be improved as well. Overall, it will certainly result in a much improved version. Hence, while your manuscript is not currently acceptable for publication in GENETICS, we would welcome a substantially revised manuscript. Reviewers have substantial comments and concerns that need to be addressed point by point in a revised manuscript. You can read their reviews at the end of this email. We look forward to receiving your revised manuscript. Please let the editorial office know approximately how long you expect to need for revisions.

Upon resubmission, please include:
1. A clean version of your manuscript;
2. A marked version of your manuscript in which you highlight significant revisions carried out in response to the major points raised by the editor/reviewers (track changes is acceptable if preferred);
3. A detailed response to the editor's/reviewers' feedback and to the concerns listed above. Please reference line numbers in this response to aid the editor and reviewers.

Your paper will likely be sent back out for review.

Additionally, please ensure that your resubmission is formatted for GENETICS
https://academic.oup.com/genetics/pages/general-instructions

Follow this link to submit the revised manuscript: Link Not Available

Sincerely,

Thomas Lenormand
Associate Editor
GENETICS

Approved by:
Nicholas Barton
Senior Editor
GENETICS

Reviewer #1 :

Summary

The authors use a mathematical model to investigate the optimal way to administer 2-strike cancer therapy: when to switch from drug 1 to drug 2 to minimize the probability of resistance to drug 2. They assume a fixed number of resistance mutations (arbitrarily chosen or at mutation-selection balance) at the time drug 1 is applied. The dynamics in drug 1 are treated deterministically, with susceptibles declining, cells resistant to drug 1 arising and increasing, cells resistant to drug 2 arising and declining, and cells resistant to both drug 1 and 2 arising and potentially establishing. At some point the patient switches to drug 2, and the authors apply stochastic evolutionary rescue theory to find the probability of resistance to drug 2. It is best for the patient to switch drugs near the minimum in population size, but it could be slightly before or after this minimum depending on parameter values. Analytical results are backed up and extended with stochastic simulations.

I find the presentation clear, the problem conceptually interesting, and the application important. Coming from the evolutionary rescue side of the literature, I am excited to see the case of multiple abrupt environmental shifts analyzed and the connection

between evolutionary rescue and drug resistance in cancer strengthened. Below are a few bigger picture comments and then a slew of minor suggestions and questions.

Major comments

1. While the presentation of the paper is very clear, in many places results are stated without added intuition. For example in section 3.1 it is stated that de novo rescue is minimized if the switch happens after the minimum population size, but it is not explained why. See more examples below in the minor comments. I think many readers will be reading the paper for intuition rather than application, and so providing the intuition will give the paper bigger impact. The discussion section is particularly light on intuition, pehaps because some intuition has already been given in the results. I think it would be worth repeating the intuition, especially since many readers may skip to the discussion for exactly that.

2. I am very happy to see an exchange of ideas between evolutionary rescue and cancer. I think this paper has the chance to really help cross-talk between these fields. I therefore would like to see more citations of the rescue literature. For example, Orr & Unckless 2014 also calculate minimum population sizes and the times they occur. I've tried to lay out all the missed connections I see in the minor comments.

3. I'm surprised it is OK to treat the mutants deterministically in drug 1, especially the rare subcritical cells that are only resistant to drug 2, which are also so critical for rescue. I know section A.4 deals with this, but perhaps more could be said at the end of section 3.3 in the main text. A figure that compares the predicted dynamics of R2 vs time with mulitple simulation replicates might also clarify why the deterministic approximation is working (and why it breaks down at large b+d).

Minor comments

- line numbers would have been helpful

intro
- I would rather say that rescue arose from conservation than is conventionally applied to conservation -- I think it is now very well known, and applied, that rescue can be good or bad (eg, it is bad in herbicide resistance, epidemiology, antimicrobial drug resistance, etc)
- what mechanism causes Allee effects in cancer?
- in the clinical trials, will they give the second drug even if there is no sign of resistance/relapse? that would come at a pretty big cost to the patient, no?

methods
- make it clear that only drug 2 in environment 2, not adding drug 2 to drug 1 (ie, sequential rather than combination)
- clarify that SGV means anything arising prior to E2 (it could equally have been defined as anything arising before E1)
- table 1: where do the parameter values come from? what is the relevant time unit?
- 2.3: why does a slight difference between N_0.1 and N_0.9 imply the extinction probability rises steeply between these two population sizes?

results
- opening section overly repetitive with methods
- 3.1: why are the last two integral terms (de novo rescue) minimized after Nmin? Because there will be fewer susceptible cells left and, even though there will be more R1 cells, they decay faster than susceptibles in drug 2 because of the cost of resistance.
- 3.2: R12 cells are generated at rate \mu R1 and R2 cells decay at rate \delta_1 - g_R. Does this help clarify when the final point of this subsection holds, that effective treatment extends further to the right of the nadir? It requires \mu R1 < \delta_1 - g_R. If the RHS is O(1) then we need R1>1/\mu for this point to fail?
- 3.3: I find the paragraph about R2(0)=0 a bit strange here, as it seems unrealistic to have R2(0) but then have R2 generated by mutation in E1. Maybe this paragraph would fit better at the end of subsection 3.2, where it could bolster the argument that it is the presence of R2(0) that makes it better to implement the second strike after the nadir?
- 3.3: I'm confused about the Poisson approximation comments. In Orr and Unckless 2008, the equivalent of exp(-\pi_e R2) comes from an approximation of (1-\pi_e)^R2. It is not that they assume the number of R2 is Poisson distributed. Rather, they assume R2 is at a deterministic mutation-selection balance. To allow for stochasticity in R2 they integrate exp(-\pi_e R2) over R2 at mutation-selection-drift equilibrium. Given you have the distribution of R2, this is something you could do too.
- 3.3: Some intuition could be added to the Poisson breakdown. It breaks down at large b+d, and this is because b+d is a measure of genetic drift/demographic stochasticity?
- 3.5: It is not so surprising that there is a limit on the size of tumours for which two-strike therapy is likely to succeed. Can we say something more about what this size is, as a function of parameter values? Or how much better is it than 1-strike?
- 3.7: why does the optimal N(\tau) increase with birth rate?
- 3.7: it would be nice to connect turnover with genetic drift (eg Martin et al 2013, Uecker & Hermisson 2011)
- 3.8: why does extinction probability increase with K? I don't understand why a lower K results in a lower decay rate of S cells and a higher growth rate of R1 cells.

discussion
- "A notable exception is when ... Figure 5, fourth row)." I don't understand how to see this in the figure.
- remind the reader what "conventional treatment" is

##appendix
- A.1: define S_0 = S(0)
- A.1: "is easy to solve" -> "can be solved exactly" (this is a general request for the entire appendix, "easy" is relative and can frustrate people, like myself!)
- A.1: explain why you are interested in R12 mutants instead of reproduction of R12 cells -- because rescue is determined by multiplynig each new mutant by the establishment probability
- A.1: R_{1,2}^*(0) is the number of R12 cells that arise before any drug is applied. This number is then multiplied by the establishment probability to get the number of rescue mutants. I was initially confused by this. But now I realize that in this model these mutants have the same establishment probability in no drug, E1, and E2. I imagine that having the same establishment probability in E1 and E2 is justifiable, and that breaking this assumption doesn't lead to qualitative differences. But I find it a bit unusual that R12 cells also establish with the same probability without any drug. I guess I am used to the pre-drug environment being a competitive one at equilibrium (eg, Wright-Fisher), but here the assumption is pure exponential growth both with and without drug. I would find it helpful to explain this.
- A.1: to "where the most important terms are those in the first line", add ", the number of mutants arising from R1 and R2 before the first drug and the number of mutants arising from R1 in the first drug."
- A.1: the t in (9) should be \tau
- A.1.1: I could use more detail about "Integrating the analog of (11)"
- A.1.2.1: the first equation in this section could be compared with eqn 6 in Orr & Unckless 2014 (PLoS Genetics), who also calculate the minimum population size and the time this occurs
- A.1.2.2: It could be helpful to mention that the relative optimal population size (eqn 13) mostly depends on a_s/a_1, and therefore on the decline rate of R1s vs Ss in the second drug, ie the cost of resistance
- A.1.2.4: "an appropriate average sense" could be made more specific by referring to harmonic means
- A.1.2.4: a t has replaced a \tau in the final eqn of this subsection
- A.1.2.5: footnote 7 could be connected with Uecker et al 2014 (Am Nat) and Day and Read 2016 (PLoS Comp Bio)
- A.1.2.5: some lowercase s's have snuck into the place of S's in the sole eqn of this subsection
- A.1.2.6: "probability of survival" - ambiguous if this is tumour or patient
- A.2: "rescue lineages become important only when the population size is much smaller than the carrying capacity" - I see what you are saying but I don't agree with the exact statement because rescue occurs whenever a lineage establishes, regardless of current population size. I would rather say that since the drugs cause tumour sizes to decrease then rescue lineages will predominantly arise and/or establish when the population size is well below carrying capacity.
- A.2: it would be nice to connect time-varying establishment with Uecker & Hermisson 2011 (Genetics)
- A.2: t_ext is the time at which the wildtype (ie sensitive) population is expected to go extinct
- A.2: In the final paragraph you say that your model extends current rescue theory in two ways: multiple abrupt changes and multiple mutations. I think this would be a nice place to make a few more connections with rescue theory. For instance, Greenspoon & Mideo 2017 TPB, Marrec & Bitbol 2020 PLoS Comp Bio, and Marrec & Bank 2023 Proc B all examine evolutionary rescue under drug cycling. And Iwasa et al 2004 JTB, Martin et al 2013 Phil Trans B, Uecker & Hermisson 2016 Genetics, Osmond et al 2020 Genetics, Stana et al 2024 bioRxiv, and Nyhoegen et al 2024 Evol Appl all examine rescue with multiple mutations.
- A.3: you say that you (numerically?) evaluate the last two integrals until population size goes to 1, but given that both S and R1 are exponentially declining in E2, can you not just approximate them with S(0)/(\delta_2 - \gamma_S) and R1(0)/(\delta_2 - \gamma_1)?
- A.4: some of the Kronecker deltas dont have a ^k
- A.4: the b^eff, d^eff and g^eff are not defined (yet)
- A.4: as mentioned above in section 3.3, add some intution for why we see a fat tail of R2 when b+d is large
- A.5.1: eqn 30 is a standard result used in previous rescue papers, such as Iwasa et al 2004 and Stana et al 2024. It would be nice to make the connections.
- A.6.2: I find this attempt to generalize the ODEs quite awkward -- why not just refer to fig 1 or write them out in full? If sticking with the general form, replace "S/R" subscripts with "M"?
- A.6.2: "now separate the positive and negative parts" -- I find this a bit confusing. Eg, in the "positive part" there is a -bN/K<0 term and a -c<0 term. I would rather you just say that make an arbitrary decision to split birth and death this way.
- A.8: I find this section difficult to follow, especially the idea that N_c only approximates the time of the drop in extinction probability well if N_0.9 is large and N_0.1 is small. Maybe some cartoon/toy supplementary figures would help?

Reviewer #2 :

The authors adapted theories of evolutionary rescue in the context of cancer chemotherapy and created the first analytical model of two-strike chemotherapy. They analyzed the behavior of extinction probabilities from both deterministic solutions and

stochastic simulations, lending theoretical support to the clinical intuition that switching chemos after the cancer cell pool has shrunk to its smallest.

Overall I enjoyed reading this manuscript, as this refreshing idea has been around for a while but few actualized the model and carried on subsequent analyses. Other than the minor comments on wording and plotting, I only have recommendations (rather than requests) that the authors consider some other possible parameter space to account for certain cancer mechanisms.

- The default model (Fig 1A) assumes that all resistant cells have the same growth rate; this is unlikely to be the case in reality, as many oncogenic mutations work by promoting cell proliferation. Maybe this could be accounted for by 1) making the cost vary by mutation, and 2) considering negative c?

- Section 3: as the \delta parameters in the model are the rate of extra cell deaths resulting from the drug, I think their values say more about the drug efficacy than the drug doses. These are two distinct quantities and don't always correlate linearly. (E.g., many have an inverted U-shape efficacy-dosage relationship.) Though the authors have acknowledged the PD and PK factors, it'd still be better to use "efficacy" instead of "dose" in this study. It'd make their values easier to explain too: a \delta=2 means the treatment makes the cells die twice as fast as they grow, whereas \delta=1.2 makes them die 20% faster than they grow, etc..

- How is "cure rate" defined? How do you quantify it? (Might be useful to move some content in Sec A.8 to the main text?)

- I'm a bit confused about what the colors on the heatmap indicate. The N_min? I don't think these heatmaps are effective/intuitive in showing the relationship between efficacies and ranges of viable N(t).

- If I understand it correctly, the way I interpret results in Section 3.4 is that the efficacy of treatment 1 doesn't need to be high in order to achieve high P_E? If so, wouldn't the subsection title be better worded as "Higher efficacies are not necessary for optimal treatment outcomes"? (Fig. 5 adds to this point too)

- Fig3: I find it very inconvenient to go to another figure to find out what the ① ② ③ ④ labels stand for. I strongly suggest the authors use proper figure legends instead. I don't think it's a good practice for figure captions to cross-reference (especially if the caption refers to a future figure).

- It might be easier for the reader to split the two panels into two separate figures so that each plot could be letter-labeled. This would make in-text references to them a lot easier too.

- I particularly like the results showing how carrying capacity doesn't noticeably affect the treatment effects. This potentially indicates ET could be applied to both primary and metastatic cancers, which would be good news for the clinical practitioners.

Reviewer #3 :

Please see attached document.

Associate Editor Comments:

Comments to Authors (GENETICS-2024-307406)

In this manuscript, Patil et al. use a combination of analytical and simulation techniques in a theoretical investigation of “two-strike” chemotherapy for cancer. The Abstract and Introduction are well written and give a nice background to the problem, particularly its clinical relevance. The Methods section is concise, and in my opinion not detailed enough. Overall, having gone through the extensive Appendices in addition to the main text, I believe the approach is generally sound, but I found the results difficult to follow. I think this issue could be addressed with substantial re-structuring to the text, clearer figure labels/captions, fixing some wording issues (with particular attention to defining terminology), and adding more detail to the main text. The Discussion reviewed some key results of the present study, but in my opinion, should have gone further in placing this study into the broader context of the literature on modelling evolutionary rescue and cancer therapies. I have provided more specific suggestions below.

In case of revisions, please could the authors add line numbers to their manuscript for ease of reference when commenting?

Main comments:

1. There is a lack of logical correspondence between results subsections and figures. Some results sections only refer to a single small sub-panel within a figure (e.g. section 3.5 only refers to “Figure 4A, second row, second panel”) whereas other sub-panels are not specifically mentioned; references to panels within the same main figure are scattered across multiple sections; and the order in which figures are mentioned in the text does not always following the order in which they appear (e.g. parts of Fig. 4 and, briefly, Fig. 5 are already mentioned in section 3.3, but Fig. 3 is not mentioned until section 3.4, before jumping back to other parts of Fig. 4 in later sections). Some figures (2, 3) do not have a one-sentence title/description at the start of the caption describing the main message, while another (Fig. 4) has a generic title (“Effects of varying parameter values or initial conditions”) that does not make the message clear. Figure 5 is mainly described in the Discussion, which is too late to be bringing up new results. Overall, I strongly encourage the authors to re-think the structure of the Results, so that each figure has a clear main message, and follows in a logical order corresponding to the main messages conveyed in the Results subsections. This might require re-thinking which plots/panels belong together in one figure, and which plots belong in main vs. supplementary. It might also call for reconsidering what really are the main findings of this study, and whether all the current subsections of the results are equally important, or if some points should be combined or shifted to supplementary.
2. The figures would benefit from clearer labels and descriptions. The plots tend to be quite complex, which is not necessarily a problem, but does call for careful exposition. I think this will already be greatly improved if the authors re-think the order and main message of each figure as suggested above. Some more specific suggestions for clarity on the plots follow – these are individually minor, but

collectively would help a lot. (I have only referred to main figures here, but similar comments apply to the supplementary figures.)

a. N_q vs. q plots (Fig. 2C, Fig. 4A): These are clever but perhaps the hardest to get one's head around. I suggest moving the explanation of how to interpret the plots from the Methods (2.3) to the Results (at first appearance of such a plot) and perhaps expanding on the interpretation. In particular explain why some curves are "cut off".

b. Again in Nq vs. q plots: There are too many curves on the same plot to easily distinguish, and it is often difficult to match up which annotated label corresponds to which curve. I suggest reducing the number of curves per plot and/or using more distinct colours/dashes if possible, and using a legend or caption description instead of the annotated labels.

c. Fig. 2B: The axis labels are unclear, please be more specific: time = τ and # of cells = $N(\tau)$? Also note that this panel is never specifically mentioned in the text, so consider whether it could be better used for illustrating a key point.

d. Heat maps (Fig. 3A, 4B) do not actually state what variable is represented by the colour. The captions say they show "high-P_E regions" but I don't see how this is shown; the scale on the colour bar is up to thousands so it can't be a probability. I was also confused why the colour bar scale is not evenly spaced? Adjacent plots should also use the same scale to avoid misleading comparisons. Finally, it was unclear what an "optimal extinction probability contour" is – optimal over what parameters/constraints?

e. Fig. 3C: it is again very difficult to see which annotated label (coloured circled numbers) matches up with which curve, and having to flip back to a different figure (2A) to find some of the labels makes it hard to follow.

f. Fig. 5 was relatively easier to follow, but could be further improved: Would it help to indicate the time window corresponding to "high P_E"? Please also clarify in the caption whether the population sizes are from ODE solutions (in absence of a second strike?).

3. Please make sure to clearly define key terms when they are <u>first</u> used, both in the main text and (where applicable) again in the appendices.

a. Pre-existing mutants (i.e. existing prior to any treatment, or prior to the second strike?) and *de novo* mutants (again, *de novo* during either treatment or only during the second?)

b. Resistant mutants versus rescue mutants; also "potential rescue mutants", as mentioned in Appendix A.1

c. Establishment: is this taken equivalent to non-extinction in a branching process model, as opposed to reaching a finite threshold population size?

d. Progression and persistence (first mentioned in section 2.2 but never defined in the main text)

e. Extinction therapy

f. Conventional treatment/therapy – this term is used in the Discussion without explanation

4. The Methods section is missing some important details:

   a. It is not sufficiently clear how $R_{1,2}$ is modelled. Fig. 1 (Part 1) shows an ODE for $R_{1,2}$ but I gather this is never actually used? This comes up again in Appendix A.1 and again it is unclear how exactly $R_{1,2}$ is modelled.

   b. During the second treatment (cf. Fig. 1, Part 2), are ODEs also used to compute $S(t)$, $R_1(t)$, $R_2(t)$ as they appear in the integrals in the P_E equations? This also needs to be stated in Appendix A.1

   c. Equation 1-2 for extinction probability is arguably the most important equation in the manuscript and would benefit from a more detailed explanation. It would be helpful to identify what contribution each term in line 2 represents. (This is especially relevant later, e.g. in section 3.1 where you refer to particular terms.) Also clarify, to which resistant strain(s) is the establishment probability relevant (only those resistant to drug 2?). State in words what the mentioned parameters b, d, c represent.

   d. Consider moving more material from Appendix A.2 into the main Methods; a lot of this seems important for understanding the approach.

   e. It could be more clearly emphasised already in Methods that you will consider two possible switching criteria: based on population size, or based on time. Throughout the Methods and Results, this distinction was not always obvious but is a critical point for understanding some of the parameter effects that you describe.

   f. More detail on the stochastic simulation methods should be included in the main text. In particular, it is unclear what you mean by "100 independent runs, each with several switching points" in the caption of Fig. 2, or "100 paired simulations with different random seeds" in the caption of Fig. 3. It is also unclear whether all parts of the model were fully stochastically simulated, or whether some parts still rely on the derived equations. In particular, Appendix A.6.3 seems to suggest that extinction probabilities are still "calculated" in the simulation, which does not seem like a sufficient check on the quality of the mathematical approximations.

5. Some of the results are quite complex and subtle, and I often found the authors' verbal explanations hard to parse. Several wording issues came up repeatedly:

   a. I repeatedly struggled to see what the authors meant by "high-P_E region": region in what space? (Population size?) Could this "region" be indicated on the relevant plots?

   b. When the term "initial" is used, it isn't always clear whether it refers to the start of treatment 1, or the start of treatment 2.

   c. The term "benefit" is used loosely and it isn't always clear what kind of benefit – to the patient or to the tumour? (This was particularly confusing when phrased as "the benefit of a cost of resistance".)

d. Likewise, the term “optimal” is not always clear – optimal under what criteria, with respect to what parameters?

It will also generally be helpful to add definitions of key terms (main comment 2 above) and ensure it is clear from the outset when the results refer to a fixed-size versus fixed-time switching criterion (cf. main comment 3d above). (Sometimes, this point was mentioned but only very late, e.g. bottom of p.7 after presenting results that crucially rely on this assumption.) I have highlighted other, specific wording issues in “Other minor comments” below.

I have two other general suggestions the authors could consider to help with exposition of their results:

e. Showing more plots simply of the population dynamics (S, R_1, R_2, $R_{1,2}$ over time), under different parameter regimes, could be extremely helpful in building the reader’s intuition and illustrating some of the finer points the authors describe – e.g. trade-offs associated with minimising the S population versus minimising the R_1 population at the time of the treatment switch.

f. Just from reading the main text, it would be easy to miss the extent of analytical results derived in the Appendices. Importantly however, these results also come with a few caveats (e.g. derived for a model with no competition; certain approximations are only valid under certain parameter conditions). I think the authors could make better use of their extensive analytical work by making it clearer in the main text what they have done: e.g. a brief summary of the approach/assumptions in Appendix 1 could be included in the main Methods. They could also consider putting a couple of key equations (e.g. maybe for N_{min}, $RM(\tau)$) into the main Results.

6. I felt that several important points were missing from the Discussion:

   a. The authors mentioned various multi-drug treatment strategies in the Introduction, and I immediately wondered why two-strike therapy (sequential use of two drugs) would ever be theoretically preferable to simultaneous use of both drugs from the start? However, this point was never addressed in the Discussion. I imagine there would be clinical arguments e.g. around drug toxicity, but this aspect is neglected in the model. Wouldn’t simultaneous treatment be optimal for maximising extinction probability? This question has been widely debated in the drug resistance literature and I strongly suggest it should be touched upon here.

   b. In general, the Discussion does not go far enough to place the present study in the context of previous work modelling the impact of treatment strategies on evolution of drug resistance in cancer. The authors state that extinction therapy has been modelled previously (ref. 16), and claim that their work “yields clearer explanations and more general results” but it is unclear how. Meanwhile, they have not cited some other studies that also modelled stochastic extinction in cancer treatment, e.g. Hansen & Read, 2020 (doi:10.1111/eva.12994). There is a brief discussion of alternative treatment strategies on p.13 but this should be expanded; please also explain/remind

the reader what “conventional therapy”, “extinction therapy”, “adaptive therapy” mean.

c. The authors only briefly mention a crucial point: that realistic “tumour detection thresholds are much higher than the initial population sizes we consider”. This warrants a much deeper discussion: How should switching strategies be designed in practice? Given that the modelled population sizes are always below detection, how does the present study then inform realistic switching strategies? How can switching after the nadir be considered the “safer” strategy, if the nadir cannot be observed?

d. The role of mutation rate, as found in the present study (section 3.6), is unsurprising. The result that mutations increase the chance of evolutionary rescue is essentially built into the model, since the only mutations considered are those that lead to treatment resistance. This effect of mutation rate is well known in the evolutionary rescue literature (reviewed e.g. the manuscript’s ref. 10), and should be placed in that context if highlighted at all. Given this model limitation, it seems inappropriate to suggest that the model has any bearing on “mutagenic therapies”, which crucially rely on the accumulation of deleterious mutations, which are not modelled here. If the authors go down this route, they should provide a clinical reference for mutagenic therapies, and point to existing model(s) in the evolutionary rescue literature that do explore deleterious mutations alongside rescue mutations (I am aware of only one: Anciaux et al. 2019, doi:10.1111/evo.13771).

e. The result that extinction probability increases with turnover (section 3.7) has also been found by several other studies, besides the one (ref. 21) cited in the Discussion: see e.g. Raatz & Traulsen 2023 (doi:10.1093/evolut/qpad042) and Czuppon et al. 2023 (doi:10.1371/journal.pcbi.1011364).

Other minor comments on main text:

1. Consider whether a more specific title would be more suitable – there are other papers addressing evolutionary rescue in cancer; perhaps highlight the specific focus on two-strike therapy?
2. Start of Methods and later: I found it strange to refer to a “deterministic analytical model” where extinction probabilities are being calculated, fundamentally indicating an underlying stochastic process. I suggest it would be more suitable just to call this an “analytical approach” (to what is, effectively, a hybrid deterministic/stochastic model), in contrast to a “(stochastic) simulation approach”.
3. It may be worth explicitly mentioning the assumption that resistance is “complete”, i.e. resistant strains are not affected at all by the corresponding drug.
4. Figure 1 (Part 2) seems to suggest that the population is at equilibrium prior to treatment. It would be helpful to state explicitly in the Methods that equilibrium is

not necessarily assumed (neither demographic equilibrium, nor population genetic equilibrium, i.e. mutation-selection balance).

5. Figure 1: The caption describes the solid curve in the Part 2 plot but not the dashed curve. In general, the description of Part 2 is brief/incomplete in the caption.
6. Section 2.3: Please clarify what you mean by “in absence of a second strike”: In this case do you assume that treatment with drug 1 continues indefinitely?
7. Section 2.3: “Any given switching point $N(\tau)$ is reached twice…” – this was confusing wording. A given population size $N(\tau)$ would be reached twice if treatment 1 simply continued, but a switch to treatment 2 only occurs once. It might help to emphasise that you are using population size as a switching rule here.
8. Last line before Section 3.1: “the maximum population size threshold that must be crossed” – this is unclear wording (crossed in which direction?) and “must be crossed” could simply be deleted for a clearer sentence.
9. Section 3.1: “the decay in the R2 population relative to…” – I’m struggling to parse this sentence or see how it explains the optimal $N(\tau)$.
10. Taken at face value, the title of section 3.2 (“It is better to implement the second strike after the nadir than before”) directly contradicts the statement above within section 3.1 that “the optimal switching point may… lie slightly before or after N_{min}”. Please clarify.
11. Section 3.2 (p.6): The explanation of the pre-existing R2 population is hard to follow. It sounds like the hypothesis proposed would be sensitive to R2(0); but is the default value realistic? The argument that delaying switching results in a smaller rescue population during the second treatment is hard to infer from the referenced figure, which shows varying R2(0), not $R2(\tau)$.
12. Section 3.3, first paragraph on p.7: I found this explanation very hard to follow. Not all of the referenced figures/curves within the plots clearly illustrate or agree with the statements made. The meaning of “optimal” extinction probability is unclear. It would also help to be clearer from the outset that the switching criterion here is a certain population size, not a certain time.
13. Section 3.3, last paragraph: “intermediate” treatment dose is unclear – there are only two doses illustrated.
14. Section 3.4: Most of this section deals with a switch implemented before the nadir, where it turns out that a higher dose is not always best. However, earlier in the Results (section 3.2) it was already stated that a switch after the nadir is better. So why not just stick with switching after the nadir, then the highest possible dose is unambiguously best? (In other words, why do we still care about before-nadir results at this stage? This is interesting theoretically but the emphasis seems misplaced.)
15. Section 3.7 (second paragraph): the “good” region is too vaguely defined.
16. Section 3.7 (last paragraph): “turnover <u>may</u> relate to the establishment probability”? Can the authors be more definitive?

17. Section 3.8: I found this section hard to follow. The referenced figure does not clearly illustrate the effects described in the text; e.g. K = 10 N(0) is not plotted. I don't follow why lower K results in "a lower decay rate for S cells and a higher growth rate for R1 cells". The phrase "both factors are present" is also unclear.
18. Discussion, section "When do we get the optimal extinction probabilities?", paragraph 2: What do you mean by an extinction "rate"? How does it relate to the "high-P_E region"?
19. Discussion, 2nd-last paragraph on p.12: "the effect of changing the mutation rate might be different…" – I suggest either explaining in what way it will be different, or removing this sentence. The statement that "our results are subject to certain methodological assumptions and limitations" is extremely generic and could be applied to any modelling study, so again, either elaborate (highlighting the most important assumptions) or delete.
20. Conclusion and future directions: "two-strike therapy… could plausibly increase cancer cure rates" – increase compared to what?

Other minor comments on appendices:

1. General comment: It would be helpful to number more of the equations and refer back to them specifically whenever they are re-used/substituted in. (E.g. above Eq. 12, which expressions for $R_i(t)$ are subbed in?)
2. General comment: There is a lot of notation in the appendices and I would have found it helpful to have another table summarising all the symbols and their definitions.
3. General comment: Please ensure that wherever an approximation is made, the assumption/condition for it to hold is stated. This is often, but not always, done. Where possible, it would also help build intuition for the results to explain the interpretation of conditions required for approximations to hold. E.g. top of p.17, $exp(-(\delta_1 - \gamma_S)\tau) \ll 1$ means the S population declines fast under treatment 1.
4. Appendix A.1: Remind the reader that time 0 is the start of treatment 1 and define $S_0 := S(0)$ (presumably)
5. A.1.1, Eq. 11: The final approximation represents mutation-selection balance; I think this would be worth pointing out.
6. A.1.1, just after Eq. 12: "the R2 population is fully hidden…" – I don't understand what you mean here. Also when you say "This allows us to study…" what is "This"?
7. Throughout: "Eq. 11" and "the reference case" seem to be used interchangeably; it would be helpful always to state "the reference case (Eq. 11)" instead of just one or the other (inconsistently).
8. A.1.2.1: "Around the nadir, most tumour cells are either S cells or R_1 cells" – this should be true for the reference case but not for arbitrary $R_2(0)$.
9. A.1.2.4, 2nd line: What are "relevant" switching times?

10. A.1.2.4, 3rd paragraph: "if they hold in an appropriate average sense" – what do you mean by this? Simply that the inequality above holds?
11. A.1.2.4, 6th paragraph ("We conclude..."): Most of this paragraph seems like main text material in my opinion. However, the robustness of the result that "tumour size and the number of expected rescue mutants tends to decrease relatively quickly before the nadir, and then to rebound more slowly" should also be illustrated, and the mathematical result in footnote 6 should actually be shown.
12. A.1.2.5, 1st sentence: "not taking into account treatment toxicity" – this seems like a very important caveat that should have been stated somewhere in the main text!
13. A.1.2.5, 3rd paragraph: "a lower value of $\delta 1$ leads to... a higher growth rate of the resistant populations" – do you mean specifically R2?
14. A.1.2.5: It was unclear what the conclusion of this section was.
15. A.1.2.6: Be careful about defining "benefits" – there is particularly confusing language in this section around "benefit of costs" and "all costs of resistance are beneficial".
16. A.1.2.6, 2nd paragraph: costs "do not impact the sensitive population" – when neglecting competition?
17. A.1.2.5, 3rd paragraph: "they do not significantly affect switching time" – how do we know this?
18. P.21, footnote 7: "the net effect should be estimated more carefully" – it is unclear whether you have actually done this somewhere? (It seems important, since the main text model does include competition.)
19. A.1.2.6, final sentence: "We conclude that... a larger cost of resistance <u>might</u>..." – so was this effect indeed observed in the numerical results?
20. Appendix A.2: Haldane's 2*s* is a poor approximation of establishment probability if *s* is large, as will often be the case for drug resistant mutants under effective drug treatment.
21. A.2: I suggest avoiding the term "stress", particularly "degree of stress", since this could be confused with other biological meanings of stress (e.g. related to physiological responses of cells). I suggest simply saying "drug treatment/dose".
22. A.2, 3rd paragraph: "we make the assumption that the probability of establishment of a resistant mutant is constant throughout"... i.e. over time, within the second treatment phase? Please clarify and state why it is reasonable to assume this.
23. A.2, top of p.23: "t_{ext} is the time at which the population would go extinct if it is not rescued" – isn't this a random variable? (Or in the ODE model, the population never technically goes extinct.) Wouldn't it be easier to integrate to time infinity?
24. A.2, top of p.23: I'm not following the wording here – what are "the first and second terms"? What do you mean by "a population that only becomes resistant to the stress induced at t=0", or "a rescue population generated by mutation from N"?

25. A.3, 1st paragraph: "This [pre-existing variants] gives us a good estimate of the rescue probability due to R2 mutants." Why can *de novo* R2 mutations during treatment 2 be neglected?
26. A.3, below Eq. 15: "integrating them until the population size is equal to one" – which population? Why equal to one?
27. A.3, last paragraph: "solve" the equations would be a clearer word choice than "evolve"
28. A.4, top of p.24: "This assumption is generally valid for the established rescue lineages" – explain why.
29. Eq. 18: What does the *k* superscript denote? What does 'eff' denote?
30. A.4, below Eq. 21: "holding for k(z,t) large" – is that a reasonable assumption?
31. A.4, end of paragraph below Eq. 21: It is unclear what are the "first" and "second" terms in Eq. 20, and what about in Eq. 21?
32. A.4, bottom of p.24: G_{NR2} can be made arbitrarily close to 1 – what does this imply?
33. A.4, final paragraph (top of p.25): Was the discrepancy at intermediate treatment doses also expected?
34. A.5: Define the abbreviations BGW and SDE
35. A.5.1: It would be helpful to explain at the outset why you include two different derivations of the establishment probability. I did not understand why you invoked the Gillespie algorithm here instead of just referring to a birth-death process. The derivations leading up to Eq. 30 seemed unnecessary to include, since they are not novel, and Eq. 30 itself is a very well-known result. In the final paragraph, it is unclear what is the point of taking the limits and checking discrepancies. Please also clarify what is *z* here?
36. A.6: Why is the simulation algorithm only considered "Gillespie-like" or "a variant of the Gillespie algorithm"? The description sounds like the exact Gillespie method. If there is some subtle difference, please highlight it and explain why you used a non-standard method.
37. Table A.1: Missing $0 < N(t)$ in the "Persistence" cases
38. A.6.2: Why are there two different notations, $g_M(t)$ and $g_{S/R}(t)$?
39. A.6.2, below Eq. 35: what are "effective" birth and death rates?
40. A.6.2, 2nd-last paragraph: The choice of how to implement density dependence in birth vs. death rates is important, and should be stated in the main text as it could have an impact on the stochastic simulation results.
41. A.6.3: I'm confused by the approach of running multiple simulations with a single random seed. At which stage are you simulating in which environment(s), treatment 1 or 2? Why do you want to "eliminate differences due to stochasticity" (algorithm step 2) in a stochastic simulation?

42. A.6.3, algorithm step 6: Extinction probabilities are calculated using which equation? Wouldn’t it be a better check on the analytical approximations also to simulate the occurrence of mutations and mutant lineage dynamics?
43. A.7: This section is very brief and could easily (and usefully) have been included in the main text.
44. A.8: I’m struggling to follow this section. The drop in extinction probability is continual, not at a specific N: Do you mean an abrupt drop, maybe the steepest point of the curve? I don’t understand what you mean by “favourable for ET” (please also define the abbreviation) or “beneficial in some cases”. The end of the first paragraph is too vague: How is “ease of implementation” defined? Are “higher extinction probabilities” required for all N? Higher compared to what? Finally, what is the conclusion of this section? Which of the three metrics did you choose to use in the main text and why?

Reviewer 1

Summary

The authors use a mathematical model to investigate the optimal way to administer 2-strike cancer therapy: when to switch from drug 1 to drug 2 to minimize the probability of resistance to drug 2. They assume a fixed number of resistance mutations (arbitrarily chosen or at mutation-selection balance) at the time drug 1 is applied. The dynamics in drug 1 are treated deterministically, with susceptibles declining, cells resistant to drug 1 arising and increasing, cells resistant to drug 2 arising and declining, and cells resistant to both drug 1 and 2 arising and potentially establishing. At some point the patient switches to drug 2, and the authors apply stochastic evolutionary rescue theory to find the probability of resistance to drug 2. It is best for the patient to switch drugs near the minimum in population size, but it could be slightly before or after this minimum depending on parameter values. Analytical results are backed up and extended with stochastic simulations.

I find the presentation clear, the problem conceptually interesting, and the application important. Coming from the evolutionary rescue side of the literature, I am excited to see the case of multiple abrupt environmental shifts analyzed and the connection between evolutionary rescue and drug resistance in cancer strengthened. Below are a few bigger picture comments and then a slew of minor suggestions and questions.

We're glad to hear that the reviewer found our work to be clear, interesting and important.

Major comments

1. **While the presentation of the paper is very clear, in many places results are stated without added intuition. For example in section 3.1 it is stated that de novo rescue is minimized if the switch happens after the minimum population size, but it is not explained why. See more examples below in the minor comments. I think many readers will be reading the paper for intuition rather than application, and so providing the intuition will give the paper bigger impact. The discussion section is particularly light on intuition, perhaps because some intuition has already been given in the results. I think it would be worth repeating the intuition, especially since many readers may skip to the discussion for exactly that.**

   Our extensive revisions of our Results and Discussion include adding more intuitive explanations.

2. **I am very happy to see an exchange of ideas between evolutionary rescue and cancer. I think this paper has the chance to really help cross-talk between these fields. I therefore would like to see more citations of the rescue literature. For example, Orr & Unckless 2014 also calculate minimum population sizes and the times they occur. I've tried to lay out all the missed connections I see in the minor comments.**

Thank you for suggesting the references, which we have added to relevant sections as detailed below.

3. **I'm surprised it is OK to treat the mutants deterministically in drug 1, especially the rare subcritical cells that are only resistant to drug 2, which are also so critical for rescue. I know section A.4 deals with this, but perhaps more could be said at the end of section 3.3 in the main text. A figure that compares the predicted dynamics of R2 vs time with multiple simulation replicates might also clarify why the deterministic approximation is working (and why it breaks down at large b+d).**

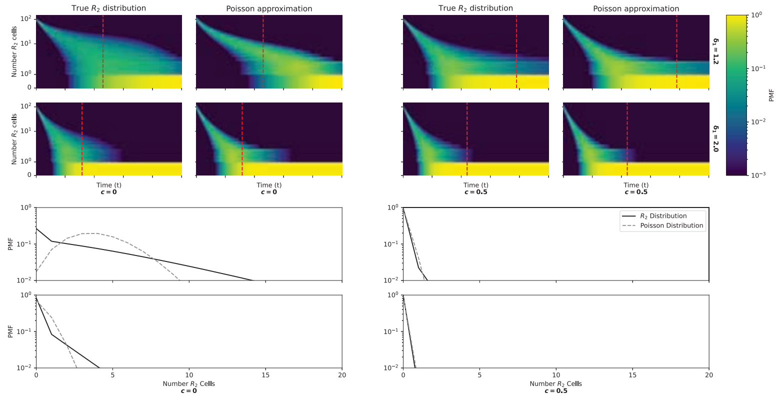

Figure 1: Distribution of R_2 cells over time by numerically solving Eq. 33 (in the main text) and by considering a Poisson approximation with rate $\lambda = R_2(t) \approx \mathbb{E}[N_{R_2}]$ in the absence of a strike. Default parameter values are used unless otherwise indicated. The dashed red line indicates when the nadir is achieved.

We should first clarify that we are *not* treating mutants deterministically. We are computing the expected number of rescue mutants from our model. We provide further justification of the correspondence between the rescue mutants calculated from the ODE model and the average number of rescue mutants generated in our stochastic model in our newly added Section A.4.2.

Assuming that these rescue mutants are Poisson distributed, knowing their expected number allows us to determine the probability that there are no rescue mutants, that is, the probability of tumour extinction. The assumption that rescue mutants are Poisson distributed turns out to be quite good for all $R_{1,2}$ rescue mutants (for a given and relatively large initial R_1 population), and for de novo R_2 rescue mutants (that is, occurring after switching to treatment 2) (see Section A.4.4). SGV R_2 rescue mutants (those occurring before switching to treatment 2) can fail to be Poisson distributed at certain parameter values (see Section A.4.3).

To computationally see this, we add a figure that shows the dynamics of R_2 using its approximate master equation formulation (Figure 1), which is explicitly written out as the first equation in Section A.4.3. These dynamics, at various parameter settings, are compared to a Poisson distribution using rate parameter $\lambda = R_2(t)$, where $R_2(t)$ is the population under the deterministic model (and which is also the mean population of the master equation formulation). At parameter settings of small c and small δ_1 (which yields a large effective birth rate compared to death rate), the true distribution has a much larger variance than what a corresponding Poisson approximation would suggest, and at larger c and larger δ_1, the true distribution becomes more Poisson-like. But since it suffices to assume the R_2 population dynamics follow a Poisson distribution to obtain the probability of extinction contribution from de novo R_2 cells, the PMF of the true R_2 distribution having more mass at the 0 barrier implies that in the case of small c, small δ_1 (ie $(c, \delta_1) \in \{(0, 1.2), (0, 1.6)\}$), the probability of extinction will be underestimated. And this underestimation is quite significant at relevant switching times (there is significantly less density close to the absorbing barrier near when the population achieves the nadir). Note that our assumption doesn't break down at large $b - c$, large $d + \delta_1$, in an absolute sense, but rather when the two quantities are made *comparable*.

We now refer to Appendix 4.4 in the relevant part of the Methods (Section 2.1). We also add further explanation and intuition for the Poisson assumption in the main text (Section 3.4) and in Appendix A.4.3.

Minor comments

- **line numbers would have been helpful**

  We have added line numbers.

Intro

- **I would rather say that rescue arose from conservation than is conventionally applied to conservation – I think it is now very well known, and applied, that rescue can be good or bad (eg, it is bad in herbicide resistance, epidemiology, antimicrobial drug resistance, etc)**

  Rephrased.

- **What mechanism causes Allee effects in cancer?**

  During tumour initiation, angiogenesis and growth factor signalling are hallmarks of cancer that are possible causes of Allee effects in cancer [1, 2, 3, 4]. We now mention these mechanisms in the Introduction.

- **in the clinical trials, will they give the second drug even if there is no sign of resistance/relapse? that would come at a pretty big cost to the patient, no?**

  Indeed, two of clinical trials we cite [5, 6] switch to another drug regimen when the tumour burden has been reduced (and prior to evidence of relapse). For

example, in the study related to prostate cancer [6], they switch to the second treatment if the patient's PSA has reduced below a certain threshold. These trials assess the effectiveness of what they call "first strike-second strike" therapy. Moreover, a clinical trial of patients with breast cancer [7] is being conducted to assess the proportion of patients able to complete two-strike therapy, along with its safety and tolerability. In our study, examining two-strike therapy from such a perspective is not the main goal, and in any case, we condition on cases where single-strike therapy or other conventional therapies fail to be curative.

It is natural to want to wait for evidence of relapse before switching to the second treatment, in case the first treatment works permanently and cures the patient. But if previous data on a given treatment tells us that it is unlikely to be curative then waiting for signs of relapse before switching treatment may represent a lost opportunity to cure the patient. Indeed, our analysis confirms that the tumour is more likely to go extinct if treatment 2 starts when the tumour is close to its nadir. Thus, if the evidence of relapse comes long after the nadir is reached, waiting for such evidence before switching to treatment 2 leads to a lower probability of cure than switching earlier.

We have revised the Discussion subsection "When should two-strike therapy be used?" to acknowledge limitations of the approach. This includes adding the following caveat: "Moreover, even when it is predicted to be the best strategy, some patients and physicians might be reluctant to initiate a second treatment before the first has been seen to fail."

Methods

- **make it clear that only drug 2 in environment 2, not adding drug 2 to drug 1 (ie, sequential rather than combination)**

  We have added a sentence to the Methods to clarify this point.

- **clarify that SGV means anything arising prior to E2 (it could equally have been defined as anything arising before E1)**

  We have added definitions of the different rescue populations to the Methods section, where we first introduce them.

- **table 1: where do the parameter values come from? what is the relevant time unit?**

  All parameter values are expressed in per-day units. We now clarify this and include a new subsection in the Methods explaining how we chose our default parameter values.

- **2.3: why does a slight difference between N_0.1 and N_0.9 imply the extinction probability rises steeply between these two population sizes?**

  We have added an explanation of how to interpret the N_q plots and an explanatory figure in the appendix (Figure S1).

Results

- **opening section overly repetitive with methods**

  We have edited the opening sections of the Methods and Results to make them less repetitive.

- **3.1: why are the last two integral terms (de novo rescue) minimized after Nmin? Because there will be fewer susceptible cells left and, even though there will be more R1 cells, they decay faster than susceptibles in drug 2 because of the cost of resistance.**

  We now provide a similar explanation in the main text, Section 3.1.

- **3.2: R12 cells are generated at rate μ R1 and R2 cells decay at rate δ_1 - g_R. Does this help clarify when the final point of this subsection holds, that effective treatment extends further to the right of the nadir? It requires $\mu \cdot R_1 < \delta_1 - g_R$. If the RHS is O(1) then we need $R1 > 1/\mu$ for this point to fail?**

  This remark is related to the analysis in Appendices A.1.2.2, A.1.2.3, and A.1.2.4. We have substantially rewritten Appendices A.1.2.2 and A.1.2.4. We tried to provide more intuition and to explain the conditions on parameters and on the initial R_2 population that are needed for switching after the nadir to be safer. We hope that the logic is now clearer. We also re-explain it below.

  There are two sources of potential rescue mutants: de novo rescue mutants (R_2 and $R_{1,2}$ arising after switching to treatment 2) and standing genetic variation (SGV) rescue mutants (R_2 and $R_{1,2}$ existing when switching). In a nutshell, your remark goes in the right direction, but focuses on SGV rescue mutants only, forgets that R_2 cells do not only decay but also appear by mutations from S to R_2, and there is an R_2 missing in the last two formulas (a typo I guess). Here are details (note that in order to focus on key points, small approximations are made below, such as $g_R - \mu \approx g_R$, and we just consider the case where the cost of resistance is the same for all resistant cells as in the main text. This is why some formulas are slightly different than in Appendix 1).

  There are two sources of rescue mutants:

  - de novo rescue mutants : in the absence of resistance costs, the expected number of de novo rescue mutants only depends on tumour size when switching to treatment 2. In the presence of a resistance cost for R_1 cells however, for a given switching size, the number of de novo rescue mutants is smaller after the nadir than before, because after the nadir, there is a higher proportion of R_1 cells, which decay quicker than S cells. You explain this very well in your point 3.1. above. We mention it in the main text, and with greater details in Appendix A.1.2.2. This is the first reason why effective treatments extend more to the right than to the left of the nadir (or said otherwise, for the same switching size, it is best to switch after the nadir than before).

  - SGV rescue mutants: $R_{1,2}$ cells are indeed generated at rate μ R_1, and R_2 cells decay at rate $\delta_1 - g_r$, but they are also created by mutations from S cells at rate

$\mu S/R_2$, so the total "decay rate" is $\delta_1 - g_R - \mu S/R_2$. As a result, the number of rescue mutants decreases if $\mu R_1 < (\delta_1 - g_R)R_2 - \mu S$. Using the approximation $N \approx S + R_1$, this may be rewritten as

$$\frac{R_2}{N} > \frac{\mu}{\delta_1 - g_R}$$

as implicitly follows from Appendix A.1.2.3 where it is shown that the number of SGV rescue mutants is minimal when the above inequality is an equality. For our parameters values and a relatively large initial value of $R_2(0)$, as assumed in our simulations, this condition is satisfied at the nadir. It follows that the number of SGV rescue mutants is still decreasing at the nadir. This is the other reason why, in our simulations, effective treatments extend more to the right than to the left of the nadir.

In the model without competition studied in the appendix, both effects can be studied together using Eq. 15, which says that the number of rescue mutants is approximately:

$$\text{RM}(\tau) \approx \mu[\alpha_S N(\tau)+(\alpha_1-\alpha_S)R_1(\tau)]+\pi_2\widetilde{R}_2(0)\exp(-(\delta_1-g_r)\tau)+C \quad \text{(C1)}$$

where C is a constant, α_1 and α_S are defined in Eq. (16) of the Appendix, and $\widetilde{R}_2(0) = R_2(0) - \frac{\mu S_0}{c}$ may be positive or negative.

In terms of switching size, to say that the window to strike is wider on the right than on the left of the nadir is to say that if $\tau_1(N), \tau_2(N)$ denote the first and second time where the population reaches size N, then, at least for N close to the minimum population size,

$$\text{RM}(\tau_1(N)) - \text{RM}(\tau_2(N)) > 0$$

or equivalently

$$\mu(\alpha_1 - \alpha_S)\left[R_1(\tau_1) - R_1(\tau_2)\right] + \pi\widetilde{R}_2(0)\left[1 - e^{-(\delta_1-g_r)(\tau_2-\tau_1)}\right] > 0$$

where $\tau_i = \tau_i(N)$. This may be rewritten as:

$$\pi\widetilde{R}_2(0)\left[1 - e^{-(\delta_1-g_r)(\tau_2-\tau_1)}\right] > \mu(\alpha_1 - \alpha_S)\left[R_1(\tau_2) - R_1(\tau_1)\right] \quad \text{(C2)}$$

where both brackets are positive.

In the reference case of Eq. (19) in Appendix A.1.1, $\tilde{R}_2(0) = 0$, so the left-hand side is 0, and this boils down to $\alpha_1 < \alpha_S$. As discussed in Appendix A.1.2.4, this condition is not met with our parameter values, due to the high cost of resistance $c = 0.5$ we consider, but it would be satisfied for slightly lower costs of resistance, namely $c \leq 0.47$.

In our simulations however, $\tilde{R}_2(0)$ is positive and relatively large, and condition (C2) is met. We thus get $RM(\tau_2(N)) < RM(\tau_1(N))$, hence effective treatments extend more to the right than to the left of the nadir.

An additional remark: in terms of switching size, what we find is that when the maximal probability of tumour extinction is obtained by switching after the nadir, the region of high probability of tumour extinction is mostly after the nadir, which is intuitive. The situation is more interesting in terms of switching times. Indeed, for a low initial R_2 population, it may be that the optimal switching time is before the nadir, yet it is safer to switch after, in the sense that, in terms of switching times, the region of high probability of tumour extinction extends more to the right than to the left of the nadir.

To see this, consider the reference case of Eq. (19) where $\tilde{R}_2(0) = 0$. Then minimizing $RM(\tau)$ boils down to minimizing the bracket in (C1). Since during treatment 1, $N \approx S + R_1$, this is equivalent to minimizing $\alpha_S S + \alpha_1 R$. But with the values of our parameters, during treatment 1, the sensitive population decays faster than the population of R_1 grows: $\delta_1 - g_S > g_1$. As a result, tumour size tends to decrease quickly before the nadir and rebound more slowly. Since we are talking about exponential decay or growth rate, this quickly makes a large difference. So for comparable values of α_1 and α_S, even when the optimal switching point is before the nadir (that is, when $\alpha_S < \alpha_1$), it may be safer to switch after the nadir than before, in the sense that for $\delta > 0$ and not too small, $RM(t_{min} + \delta)$ quickly becomes substantially smaller than $RM(t_{min}) - \delta$. This conclusion is reversed if $\delta_1 - g_S < g_1$, as we now mention in the manuscript.

In addition, in our simulations, $\tilde{R}_2(0) > 0$ so waiting for the SGV R_2 population to decay is an additional motivation to switch after rather than before the nadir. All the more so that the decay rate of R_2 is then larger than the growth-rate of R_1. This reinforces the fact that the number of rescue mutants decreases quickly before reaching its minimum and then rebounds more slowly.

- **3.3: I find the paragraph about R2(0)=0 a bit strange here, as it seems unrealistic to have R2(0) but then have R2 generated by mutation in E1. Maybe this paragraph would fit better at the end of subsection 3.2, where it could bolster the argument that it is the presence of R2(0) that makes it better to implement the second strike after the nadir?**

  We have restructured the Results and moved the subsection about the effect of varying $R_2(0)$ to Section 3.2.

- **3.3: I'm confused about the Poisson approximation comments. In Orr and Unckless 2008, the equivalent of $\exp(-\pi_e R_2)$ comes from an approximation of $(1 - \pi_e)^{R_2}$. It is not that they assume the number of R2 is Poisson distributed. Rather, they assume R2 is at a deterministic mutation-selection balance. To allow for stochasticity in R2 they integrate $\exp(-\pi_e R2)$ over R2 at mutation-selection-drift equilibrium. Given you have the distribution of R2, this is something you could do too.**

  Thank you for the reference. There might be a slight confusion. We don't obtain the expression $\exp(-\pi_e R_2)$ from an approximation of $(1-\pi_e)^{R_2}$ (or I guess not in the sense that literally this approximation holds for π_e small and R_2 large, for R_2 a deterministic large quantity), but instead using the Poisson approximation.

We have now noted this difference in Appendix A.2. Furthermore, R_2 denotes the deterministic R_2 population (implicitly at time t), not a random variable. We denote in section A.4.3 N_{R_2} as the random variable representation of this population (it happens that $\mathbb{E}[N_{R_2}] = R_2(t)$).

Here is the constructive picture: Suppose N is some population that happens to be Poisson distributed (with rate λ), π is the probability of establishment of an individual cell, and that the establishment of a cell line deriving from one of the $N(\omega)$ members is independent of the establishment (or non-establishment) of others. Denote by N^e the random variable corresponding to the number of established cells. Then, for any particular realization of N (ie for a fixed $\omega \in \Omega$ the sample space), $N^e|N \sim \text{Bin}(N, \pi)$.

But note via iterated conditional expectation $G_{N^e}(z) = \mathbb{E}[z^{N^e}] = \mathbb{E}[((1-\pi) + \pi z)^N] = G_N(1 - \pi + \pi z)$, as shown in Appendix A.4.3, where G_X is the probability generating function (PGF) of random variable X. The PGF of a Poisson distribution is $\exp(\lambda(z-1))$, hence $G(1-\pi+\pi z) = \exp(\lambda(1-\pi+\pi z-1)) = \exp(\pi\lambda(z-1))$. But the probability of extinction is simply the PMF of N^e evaluated at 0, which is the PGF of N^e evaluated at $z = 0$, which is the (as expected) expression $\exp(-\pi\lambda)$. Hence, it *suffices* to use a Poisson approximation to obtain this expression. The important question is then how necessary this assumption is. Section A.4 deals with this by calculating (to some approximation) the true PGF and then the probability of extinction of N_{R_2}. We find under some assumptions, like $\frac{b-c}{d+\delta_1} \ll 1$, we reclaim our expected expression (and according to the figure this also corresponds with the N_{R_2} population being approximately Poisson), but once we deviate to the other extreme of $\frac{b-c}{d+\delta_1} \approx 1$, as noted in Appendix A.4.3, the true expression can be made strictly larger (i.e. our expected expression would be an underestimate of the extinction probability).

- **3.3: Some intuition could be added to the Poisson breakdown. It breaks down at large b+d, and this is because b+d is a measure of genetic drift / demographic stochasticity?**

  It breaks down when $b - c$ is comparable to $d + \delta_1$ (ie $\frac{b-c}{d+\delta_1} \approx 1$, or small c with small δ_1), which for example occurs at constant g and large $b + d$. The intuition for this breakdown at constant growth rates but sufficiently large turnover is that turnover is roughly proportional to the variance of the R_2 population.

  Consider an R_2 cell during treatment 1. Fixing $b - d - \delta_1 < 0$, the probability that this cell dies before reproducing goes to 100% in the limit $b = 0$ (minimal turnover) to just over 50% in the limit $b \to +\infty$ (maximal turnover). The latter case generates much more variance in the R_2 population. But for a given expected R_2 population, the probability that $R_2(\tau) = 0$ is larger when the variance is large. Thus, a large turnover leads to a larger variance in the R_2 population than in a Poisson distribution, which in turns leads to a larger probability that there are no SGV rescue mutants and a larger probability of extinction than predicted by our model.

  We have added this explanation to Appendix A.4.3 and have added the following sentences to Section 3.4: "This breakdown occurs when the variance of the R_2

population is higher than in a Poisson distribution, which leads to a higher probability that there are zero pre-existing rescue mutants before the second treatment. Since turnover is a measure of demographic stochasticity, larger values of $b + d$ at a constant growth rate lead to larger variance in the R_2 population and breakdown of the Poisson assumption."

- **3.5: It is not so surprising that there is a limit on the size of tumours for which two-strike therapy is likely to succeed. Can we say something more about what this size is, as a function of parameter values? Or how much better is it than 1-strike?**

  What is easy to see is that two-strike therapy allows to deal with tumours more than 1000 times larger than with single-strike therapy, for a given probability of tumour extinction. To see this, let us consider the model without competition of Appendix A.1. We model 1-strike therapy as following the same model but with a switching time $\tau = \infty$. Let $P_0 = (S_0, R_1(0), R_2(0), R_{1,2}(0))$ denote the vector of initial conditions of the reference case (18), see Appendix A.1, Section A.1.1. Denoting by N_0 the initial total tumour size and using that $N_0 \approx S_0$, we have:

$$P_0 \approx \left(N_0, \frac{\mu_1}{c_1}N_0, \frac{\mu_2}{c_2}N_0, \mu_1\mu_2\frac{N_0}{\gamma_S}\left(\frac{c_1+c_2}{c_1c_2}\right)\right) \tag{1}$$

  Thus, the initial conditions are (roughly) proportional to the initial tumour size N_0. Since the model consists of linear differential equations, it follows that *the solutions and the number of rescue mutants generated when striking at a given time are (roughly) proportional to* N_0. It also follows that in two-strike therapy, the optimal switching time τ is independent of N_0.

  Let us compare 1-strike therapy to a 2-strike therapy switching at this optimal switching time τ. Let

$$P_0^1 = \frac{P_0}{N_0} \approx \left(1, \frac{\mu_1}{c_1}, \frac{\mu_2}{c_2}, \mu_1\mu_2\frac{1}{\gamma_S}\left(\frac{c_1+c_2}{c_1c_2}\right)\right)$$

  denote the vector of initial frequencies of the various types of tumour cells. For $i = 1, 2$, let $RM^i(P_0)$ and $RM^i(P_0^1)$ denote the number of rescue mutants generated by i-strike therapy if the initial condition is P_0 and if the initial condition is P_0^1, respectively. The formula used for 1-strike therapy is:

$$\mathrm{RM}^1 = \pi_e R_{1,2}(0) + \pi_e R_1(0) + \pi_e\mu_1\int_0^\infty S(t)\,dt + \pi_e\mu_2\int_0^\infty R_2(t)\,dt$$

  where the term $R_{1,2}(0)$ is negligible.

  By the above emphasized observation $\mathrm{RM}^i(P_0) \approx N_0\mathrm{RM}^i(P_0^1)$. Fix a desired tumour extinction probability p_{ext}. Let N_0^i denote the tumour size leading exactly to this extinction probability under i-strike therapy. Then:

$$p_{\text{ext}} \approx \exp\left(-N_0^i\,\mathrm{RM}^i(P_0^1)\right)$$

Therefore, $N_0^i\,\mathrm{RM}^i(P_0^1) \approx -\ln(p_{\mathrm{ext}})$ hence $N_0^1\,\mathrm{RM}^1(P_0^1) \approx N_0^2\,\mathrm{RM}^2(P_0^1)$. Finally,

$$\frac{N_0^2}{N_0^1} \approx \frac{\mathrm{RM}_1^1}{\mathrm{RM}_1^2}$$

This turns out to be on the order of $3 \cdot 10^3$ using the reference case (and of $6 \cdot 10^3$ using the default population distribution from Table 1), meaning much larger tumours can be eliminated at a desired probability using two-strike compared to one-strike therapy. The analysis would be trickier with the nonlinear model of the main text (with competition), but we expect the same order of magnitude.

The above explanation now features in Appendix A.1.3.

- **3.7: why does the optimal $N(\tau)$ increase with birth rate?**

  There is a relatively easy explanation for this. Suppose that striking at or close enough to the nadir maximizes the extinction probability. Then, we may simply explore how $N_{\min}$ changes as b increases. Well, $N \approx S + R_1$ and since birth rates are increased the decay of S cells is slower and the growth of R_1 cells is faster, meaning that the new $N_{\min}$ is larger, so the optimal $N(\tau)$ should be larger.

  We have added this explanation to Section 3.7

- **3.7: it would be nice to connect turnover with genetic drift (eg Martin et al 2013, Uecker & Hermisson 2011)**

  In Uecker & Hermisson 2011, a population composed of two cell types that differ by a mutation are investigated, and specifically fixation of the mutant cell type under time-varying birth and death rates (assuming it is favourable). They show that a larger turnover decreases the probability of fixation resulting from genetic drift. This is equivalent to an increase in extinction probability in our case. We mention this reference in Section 3.7.

- **3.8: why does extinction probability increase with K? I don't understand why a lower K results in a lower decay rate of S cells and a higher growth rate of R1 cells.**

  First, we apologize for a typo: a lower K (more competition) results in a HIGHER decay rate of S cells and a LOWER growth-rate of R_1 cells. Nevertheless, the extinction probability may unexpectedly increase with K (i.e. less intra-tumour competition may lead to an increase probability of cure). We were initially puzzled by this paradoxical result. The key is that this holds only when switching treatment *at a given tumour size*, not when switching *at a given time*. Indeed, as we just said, a larger value of K (less competition) leads to a slower decay of S cells, hence to more mutations from S to R_1 or R_2 cells, as well as a slower decay of R_2 cells and a quicker growth of R_1 cells. Thus, though we did not try to prove it formally (this might be possible using variants of Grönwall's Lemma), it is intuitively clear that a larger value of K leads to a larger expected number of rescue mutants at a given time, hence to a lower probability of extinction if switching at a fixed, predetermined time.

The situation is different if the switch from treatment 1 to treatment 2 occurs at a given tumour size. To get an intuition, note that the model is such that if $K = N$, the cost of resistance does not bite. In particular, if $K = N$, in the absence of mutations, the populations of R_2 cells and S cells decay exponentially at the same rate. By contrast, if $K \gg N$, the cost of resistance bites and in the absence of mutations, the R_2 population decays quicker than the S population. So a given decrease in the S population corresponds to a larger decrease in the R_2 population.

Moreover, when switching at a given size substantially before the nadir, the switching time is largely determined by how much the S population has decreased (since we still have $S \approx N$ at the time of switching). Thus a larger value of K leads to a lower expected R_2 population when reaching the switching size, hence to less SGV rescue mutants.

This is partially compensated by the fact that a larger value of K leads to a slower decay of the S population, hence a larger number of new mutations from S to R_2 cells for a given decrease of the S population. However, if as in our simulations, we assume that the R_2 population is initially relatively large, then the first effect dominates (at least initially), and we expect less competition to lead to less SGV R_2 rescue mutants when switching to treatment 2, as we observe.

Mathematically, as long as $S \approx N$,

$$\frac{dR_2}{dS} \approx \frac{R_2}{S}\frac{\delta_1 - g_R[1 - N/K] - \mu_2 S/R_2}{\delta_1 - g_S[1 - N/K]} \approx \frac{R_2}{S}\frac{\delta_1 - g_R[1 - S/K] - \mu_2 S/R_2}{\delta_1 - g_S[1 - S/K]}$$

Letting $x = 1 - S/K$ and letting $f(S, R_2, x)$ denote the right-most expression in the above equation, it may be checked that $\partial f/\partial x$ has the sign of $(g_S - g_R)\delta_1 - \mu_2 g_S S/R_2$, which is positive if $\mu_2 S/R_2$ is relatively small, as is initially the case in our simulations. Thus we expect an increase in K (leading to an increase in x) to lead to a larger decay of the R_2 population for a given decay of the S population, as explained above.

When the R_1 population becomes significant, another effect going in the same direction appears. Indeed, it may be checked that for a larger value of K, a given decrease of the S population corresponds to a larger increase of the R_1 population (formally, $-dR_1/dS$ is increasing in K). Therefore, for a larger value of K, a larger decrease in S is needed to reach the switching size. This leads to an even stronger decay of the initial R_2 population by the time the switching size is reached, and to a larger proportion of R_1 cells, which create less de novo rescue mutants per cell than S cells.

This phenomenon is similar to the fact that a lower efficacy δ_1 of treatment 1 may paradoxically increase the probability of tumour extinction when switching at a given tumour size before reaching the nadir, as discussed in Appendix A.1.2.5. Indeed, a lower efficacy of treatment 1 also reinforces the impact of the cost of resistance on the relative speed at which the S and R_2 population decay, so that a given decrease in the S population corresponds to a larger decrease of the R_2 population when δ_1 is small.

We now write at the end of Appendix A.1.2.5: For similar reasons, a larger carrying capacity K, by reducing the decay rate of S cells, may decrease the number of SGV R_2 rescue mutants and lead to a larger probability of tumour extinction when switching at a given size and before the nadir, as discussed in Section 3.6.

Discussion

- **“A notable exception is when ... Figure 5, fourth row).” I don’t understand how to see this in the figure.**

  We added a panel to Figure 4 with a direct comparison between the case with and without the cost of resistance when switching at a fixed time (not fixed size), and we now refer to that figure instead. Figure 5 in the previous version of the paper is now moved to the appendix (Figure S3).

- **remind the reader what ”conventional treatment” is**

  Conventional treatment can either be single-strike therapy or sequential therapy in which second-line treatment is provided only after evidence of rebound rather than attempting to strike where most beneficial (i.e. from our analysis close to the nadir).

  We have added the relevant description whenever conventional treatment is mentioned.

Appendix

- **A.1: define $S_0 = S(0)$**

  Done

- **A.1: ”is easy to solve” -¿ ”can be solved exactly” (this is a general request for the entire appendix, ”easy” is relative and can frustrate people, like myself!)**

  We have removed the instances of “easy”.

- **A.1: explain why you are interested in R12 mutants instead of reproduction of R12 cells – because rescue is determined by multiplynig each new mutant by the establishment probability**

  We clarify this in Appendix A.1 and explicitly mention it when deriving the expression for extinction probability in Appendix A.3

- **A.1: $R^*_{1,2}(0)$ is the number of R12 cells that arise before any drug is applied. This number is then multiplied by the establishment probability to get the number of rescue mutants. I was initially confused by this. But now I realize that in this model these mutants have the same establishment probability in no drug, E1, and E2. I imagine that having the same establishment probability in E1 and E2 is justifiable, and that breaking this assumption doesn’t lead to qualitative differences. But I find it a bit unusual that R12 cells also establish with the same probability without any drug. I guess I am used**

to the pre-drug environment being a competitive one at equilibrium (eg, Wright-Fisher), but here the assumption is pure exponential growth both with and without drug. I would find it helpful to explain this.

We now mention this point in Appendix 1: "Note that due to the absence of competition in the above model, the probability of establishment of an $R_{1,2}$ mutant is the same in all environments, including before treatment." We add the following as a footnote: "Not considering competition is most debatable before treatment begins, as this is when the tumour is expected to be most dense. Competition would reduce the probability of establishment of potential $R_{1,2}$ rescue mutants before treatment, but not taking this fact into account is harmless for two reasons. First, even without taking competition into account, the expected number of potential $R_{1,2}$ mutants arising before treatment 1, $R^*_{1,2}(0)$, is negligible. Lowering this value would only very slightly increase the probability of extinction for a given treatment. Second, if an $R_{1,2}$ rescue mutant got established before treatment begins, then the probability of tumour extinction is zero whatever the treatment. Thus, to determine which treatment maximises the probability of extinction, we can condition on the event $R^*_{1,2}(0) = 0$. The probability of establishment of $R_{1,2}$ cells before treatment begins is then irrelevant."

- **A.1: to "where the most important terms are those in the first line", add ", the number of mutants arising from R1 and R2 before the first drug and the number of mutants arising from R1 in the first drug."**

  Done (and appended the adjective "rescue" to mutants).

- **A.1: the t in (9) should be τ**

  Done.

- **A.1.1: I could use more detail about "Integrating the analog of (11)"**

  Good point, this formulation was indeed not clear. We modified the passage to make it clearer. We numbered more equations, introduced a couple of new ones, and now refer precisely to the equations we use.

- **A.1.2.1: the first equation in this section could be compared with eqn 6 in Orr & Unckless 2014 (PLoS Genetics), who also calculate the minimum population size and the time this occurs**

  The equation we derived for the expected tumour size $N(t)$ during treatment 1 is very basic and we certainly do not claim any originality. We now write in a footnote: "[This equation] is similar to e.g. Eqn 6 of (Orr & Unckless, 2014), with the difference that the latter does not consider the impact of mutations from S to R_1 cells arising during treatment 1."

  The point of Orr & Unckless is that such an equation does not represent well the expected tumour size conditional on tumour survival under only treatment 1, which is true, but first this is not the quantity we are interested in, and second they also agree with us that their more subtle analysis is not necessary when the probability of tumour extinction (under only treatment 1) is very low, as is the case for us.

- **A.1.2.2: It could be helpful to mention that the relative optimal population size (eqn 13) mostly depends on a_s/a_1, and therefore on the decline rate of R_1s vs Ss in the second drug, ie the cost of resistance**

  We have added a paragraph detailing the intuition and explaining that the reason why the number of de novo rescue mutants is minimized by switching after the nadir is due to the cost of resistance. Given this new paragraph, we believe that adding a remark after the former Eq. 13 is no longer necessary.

- **A.1.2.4: "an appropriate average sense" could be made more specific by referring to harmonic means**

  Actually, the "appropriate" average sense was simply an arithmetic mean, but we modified the whole passage, as the reason for why this is the case became clearer to us while revising the article. Basically (up to a constant term), the number of de novo rescue mutants is of the form $x_S^{\mathrm{DN}} S + x_1^{\mathrm{DN}} R_1$, the number of SGV rescue mutants of the form $x_S^{\mathrm{SGV}} S + x_1^{\mathrm{SGV}} R_1$, and so the total number of rescue mutants of the form $(x_S^{\mathrm{DN}} + x_S^{\mathrm{SGV}})S + (x_1^{\mathrm{DN}} + x_1^{\mathrm{SGV}})R_1$, where x_S^{DN}, x_1^{DN}, x_S^{SGV} and x_1^{SGV} are appropriate coefficients. Each time, these quantities are minimized before the nadir if the coefficient of S is smaller than the coefficient of R_1, and after otherwise. Because the third expression is the sum of the first two, the condition for the total number of rescue mutants to be minimized before the nadir:

  $$x_S^{\mathrm{DN}} + x_S^{\mathrm{SGV}} < x_1^{\mathrm{DN}} + x_1^{\mathrm{SGV}}$$

  is just the arithmetic mean of conditions $x_S^{\mathrm{DN}} < x_1^{\mathrm{DN}}$ and $x_S^{\mathrm{SGV}} < x_1^{\mathrm{SGV}}$, that say when the number of de novo or SGV mutants is minimized by switching before the nadir, respectively. We do not say all of that, but we make this logic more apparent by deriving explicitly the number of rescue mutants as the sum of the numbers of de novo and SGV rescue mutants.

- **A.1.2.4: a t has replaced a τ in the final eqn of this subsection**

  Thank you, fixed.

- **A.1.2.5: footnote 7 could be connected with Uecker et al 2014 (Am Nat) and Day and Read 2016 (PLoS Comp Bio)**

  Thank you for these references, which we now cite. Yours and another reviewer's comment prompted us to examine this issue in greater detail. We realized that, indeed, in the model with competition, a larger treatment efficacy could decrease the probability of tumour extinction at a given switching time when this time is quite large. We now explain this in a footnote:

  "The situation would be more complex in a model with competition, as reducing the sensitive population quickly leads to the competitive release of the resistant populations. Thus, a larger value of δ_1 would also have negative effects, and the net effect should be estimated more carefully. For example, with a lower δ_1, the time before R_1 can grow at its competition-free rate increases, meaning that at a given time, there would be fewer R_1 cells, hence fewer R_{12} mutants arising from R_1 cells, whether before or after switching to treatment 2. So, if switching at a

large time, when S cells and SGV R_2 mutants are insignificant, a lower value of δ_1 could increase the probability of extinction. This is observed comparing the probability of extinction between $\delta_1 = 1.2$ and $\delta_1 = 2.0$ at otherwise default parameter values when switching at $\tau = 20$ (see Figure S3). Similar issues are studied by [8] and [9], e.g., pp. E31-E32, and in the literature on tumour containment [10]."

- **A.1.2.5: some lowercase s's have snuck into the place of S's in the sole eqn of this subsection**

  Fixed.

- **A.1.2.6: "probability of survival" - ambiguous if this is tumour or patient**

  Rephrased.

- **A.2: "rescue lineages become important only when the population size is much smaller than the carrying capacity" - I see what you are saying but I don't agree with the exact statement because rescue occurs whenever a lineage establishes, regardless of current population size. I would rather say that since the drugs cause tumour sizes to decrease then rescue lineages will predominantly arise and/or establish when the population size is well below carrying capacity.**

  Yes, its better to say that density independence is a reasonable assumption as pre-rescue lineages predominantly establish when the population is well below carrying capacity. We edited this line in Appendix A.2.

- **A.2: it would be nice to connect time-varying establishment with Uecker & Hermisson 2011 (Genetics)**

  Thank you. We now cite the article and also provide a figure (reproduced here) showing that it's not very important to consider time-varying establishment.

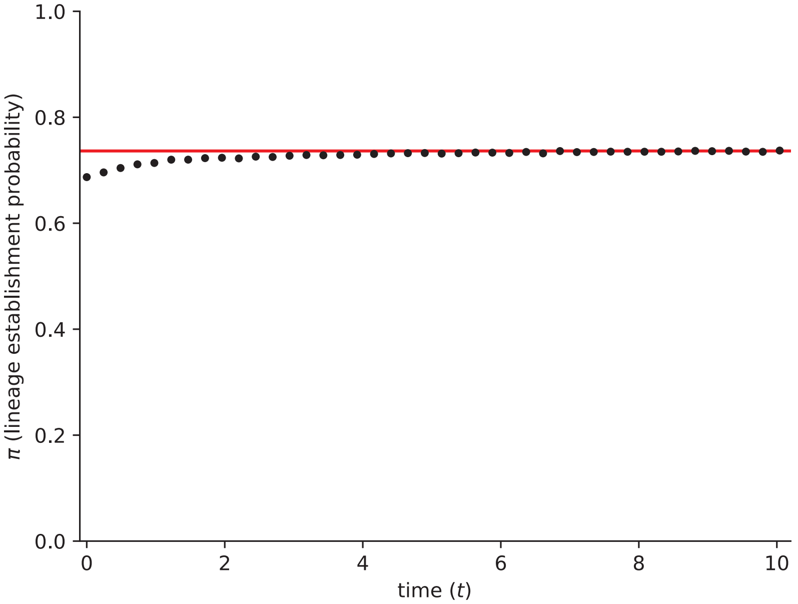

Figure 2: Establishment probability of rescue lineages, starting with 1 cell at different times, assuming competition under just S-cell dynamics. Parameters used are $b = 1.0$, $d = 0.1$, $c = 0.5$, $\delta = 1.2$. The red line represents the theoretical establishment probability assuming no competition. The black dots are generated from numeric solutions to the branching diffusion equation (15) using the Euler-Maruyama scheme with $\Delta t = 10^{-3}$ and 100,000 replicates

Even if a pre-rescue mutant appears early on, the true establishment probability differs by less than 10% from the one we use (and the probability of a pre-rescue mutant appearing so early should be quite small anyway).

- **A.2: t_{ext} is the time at which the wildtype (ie sensitive) population is expected to go extinct**

  We remove references to this as it's simpler to have the integral go to ∞.

- **A.2: In the final paragraph you say that your model extends current rescue theory in two ways: multiple abrupt changes and multiple mutations. I think this would be a nice place to make a few more connections with rescue theory. For instance, Greenspoon & Mideo 2017 TPB, Marrec & Bitbol 2020 PLoS Comp Bio, and Marrec & Bank 2023 Proc B all examine evolutionary rescue under drug cycling. And Iwasa et al 2004 JTB, Martin et al 2013 Phil Trans B, Uecker & Hermisson 2016 Genetics, Osmond et al 2020 Genetics, Stana et al 2024 bioRxiv, and Nyhoegen et al 2024 Evol Appl all examine rescue with multiple mutations.**

Thank you for suggesting these papers. We have moved the last paragraph of Appendix A.2 to the Discussion, revised it, and added references.

- **A.3: you say that you (numerically?) evaluate the last two integrals until population size goes to 1, but given that both S and R1 are exponentially declining in E2, can you not just approximate them with $S(0)/(\delta_2 - \gamma_S)$ and $R1(0)/(\delta_2 - \gamma_1)$?**

  Yes, we could make this approximation, and it would be good since the S and R_1 population during treatment 2 are much smaller than K. However, we wanted to use exact dynamics to obtain the probability of extinction (and for exact dynamics we have competition, for example). Also the formulas would be slightly different from what you suggest (probably a typo), because the integral for E_2 is over the interval $[\tau, \infty)$, not $[0, \infty)$.

- **A.4: some of the Kronecker deltas dont have a ^{k}**

  Fixed.

- **A.4: the b^{eff}, d^{eff} and g^{eff} are not defined (yet)**

  We now first reference them in Appendix A.4.1, and we point to where these terms are defined.

- **A.4: as mentioned above in section 3.3, add some intuition for why we see a fat tail of R2 when b+d is large**

  We add an explanation in the main text as well as Appendix A.4.3.

- **A.5.1: eqn 30 is a standard result used in previous rescue papers, such as Iwasa et al 2004 and Stana et al 2024. It would be nice to make the connections.**

  We now mention that Eq. 30 is well-known but have not added further references because it is a standard result for the birth-death process.

- **A.6.2: I find this attempt to generalize the ODEs quite awkward – why not just refer to fig 1 or write them out in full? If sticking with the general form, replace ”S/R” subscripts with ”M”?**

  We have replaced “S/R” subscript with “M” and added further explanation.

- **A.6.2: ”now separate the positive and negative parts” – I find this a bit confusing. Eg, in the ”positive part” there is a $-bN/K < 0$ term and a $-c < 0$ term. I would rather you just say that make an arbitrary decision to split birth and death this way.**

  We have replaced ”separate positive and negative parts” with ”split the subpopulation growth rates into effective birth and death rates”.

- **A.8: I find this section difficult to follow, especially the idea that N_c only approximates the time of the drop in extinction probability well if $N_{0.9}$ is large and $N_{0.1}$ is small. Maybe some cartoon/toy supplementary figures would help?**

We removed this section because we neither use nor refer to any of these metrics in our results. Instead, we now explain in the main text how we compare different parameter values using the N_q vs q plots (Section 2.3). We have also added an explanatory figure in the appendix for the same (Figure S1).

Reviewer 2

The authors adapted theories of evolutionary rescue in the context of cancer chemotherapy and created the first analytical model of two-strike chemotherapy. They analyzed the behavior of extinction probabilities from both deterministic solutions and stochastic simulations, lending theoretical support to the clinical intuition that switching chemos after the cancer cell pool has shrunk to its smallest.

Overall I enjoyed reading this manuscript, as this refreshing idea has been around for a while but few actualized the model and carried on subsequent analyses. Other than the minor comments on wording and plotting, I only have recommendations (rather than requests) that the authors consider some other possible parameter space to account for certain cancer mechanisms.

We are glad to hear that the reviewer enjoyed reading our work and found no need for major revisions.

- **The default model (Fig 1A) assumes that all resistant cells have the same growth rate; this is unlikely to be the case in reality, as many oncogenic mutations work by promoting cell proliferation. Maybe this could be accounted for by 1) making the cost vary by mutation, and 2) considering negative c?**

  1) Although interesting, this would substantially increase the parameter space and would be out of scope of the current study.

  2) We now perform numerical experiments with the analytical model to investigate a negative cost of resistance.

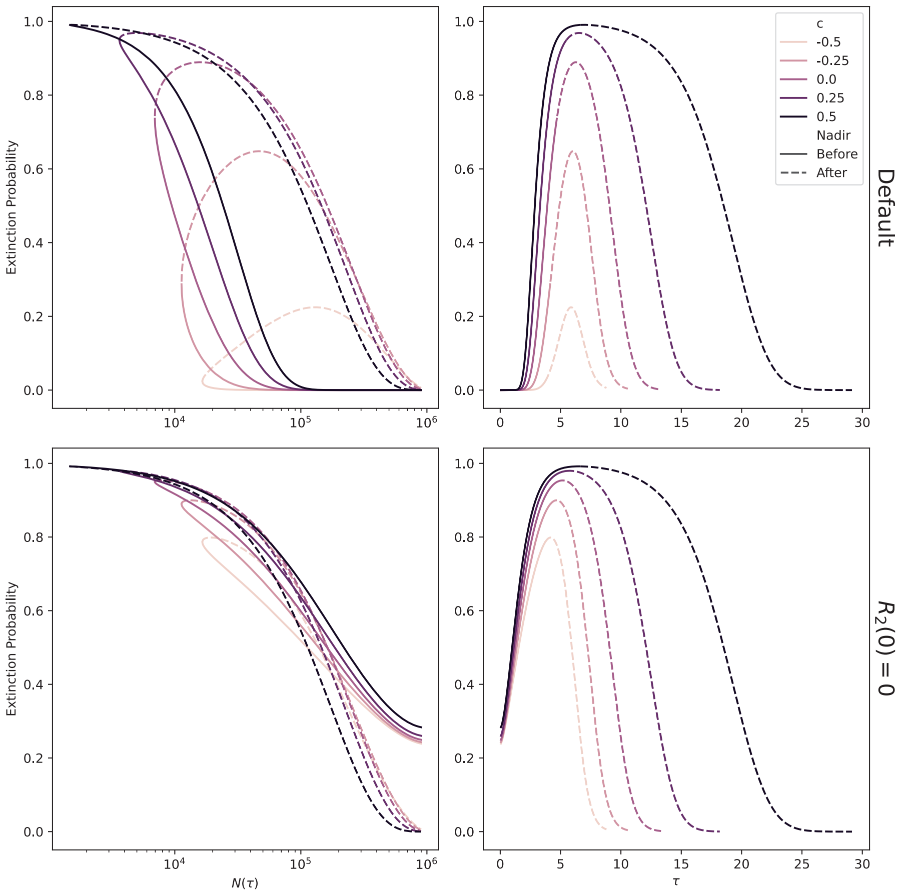

Figure 3: Probability of extinction as a function of switching size (left) or switching time (right), with costs of resistance $c \in [-0.5, 0.5]$

Under very low costs of resistance with otherwise default parameter values, the optimal striking location deviates from the location of the nadir. This is because lower costs of resistance decrease $t_{\min}$ but slow the decay of SGV R_2.

We have added a figure (Fig. S4 in the Appendix, reproduced here) and the following text: Fig. S4 shows the impact of varying the cost of resistance for the analytical model. Here, as in the main text, the same cost of resistance is applied to all resistant types: $c_1 = c_2 = c_{12} = c$. Fig. S4 also considers the case of a negative cost of resistance, that is, when resistant cells are fitter than sensitive cells even in the absence of treatment.[1] The second column illustrates that for a given switching time, the probability of extinction increases with the cost of

[1]We did not run simulations of the stochastic model for the case of negative cost of resistance. Results of the analytical model in this case should be taken with care.

resistance c. The first column illustrates that, for a given switching size, when switching long after the nadir, the probability of extinction may paradoxically decrease when the cost of resistance increases (though the optimal extinction probability increases). It also shows that with a low or negative cost of resistance, the conclusion that it is optimal to switch close to the nadir depends on the initial R_2 population. Indeed, if the cost of resistance c_2 is low or negative, the R_2 population decays more slowly during treatment 1. It may then be optimal to switch to treatment 2 substantially after reaching the nadir, in order to decrease the number of SGV R_2 rescue mutants (Fig. S4, Top-Left panel). This effect disappears if we assume a low initial R_2 population (compare the Top-Left and Bottom-Left panels of Fig. S4).

- **Section 3: as the δ parameters in the model are the rate of extra cell deaths resulting from the drug, I think their values say more about the drug efficacy than the drug doses. These are two distinct quantities and don't always correlate linearly. (E.g., many have an inverted U-shape efficacy-dosage relationship.) Though the authors have acknowledged the PD and PK factors, it'd still be better to use "efficacy" instead of "dose" in this study. It'd make their values easier to explain too: a $\delta = 2$ means the treatment makes the cells die twice as fast as they grow, whereas $\delta = 1.2$ makes them die 20% faster than they grow, etc...**

  We have changed "treatment dose" and "treatment level" to "treatment efficacy" throughout.

- **How is "cure rate" defined? How do you quantify it? (Might be useful to move some content in Sec A.8 to the main text?)**

  We are using the term "cure rate" more informally in various locations, and therefore do not define it explicitly or use it in the Methods or Results. Within the Introduction and Discussion, it is taken as the probability of a cancer patient being cured (which the probability of extinction of the tumour is a surrogate for). In the Introduction, we mention that the prevention of evolutionary rescue is equivalent to cure.

  We have removed Sec A.8 since we do not refer to alternate metrics for comparing parameter values based on the N_q curves. We have added an explanation in the main text on how to interpret the N_q curves and also added an explanatory figure (Figure S1) instead.

- **I'm a bit confused about what the colours on the heatmap indicate. The $N_{\min}$? I don't think these heatmaps are effective/intuitive in showing the relationship between efficacies and ranges of viable $N(t)$.**

  We now explain what quantity is plotted in the heatmap next to the colour bar. We plot the range of high-P_E switching sizes, that is, the difference in the largest and the smallest switching size that would give a probability of extinction ≥ 0.8. We also provide a more detailed explanation in the figure captions and the main text.

- **If I understand it correctly, the way I interpret results in Section 3.4 is that the efficacy of treatment 1 doesn't need to be high in order to achieve high P_E? If so, wouldn't the subsection title be better worded as "Higher efficacies are not necessary for optimal treatment outcomes"? (Fig. 5 adds to this point too)**

  While that is one of the conclusions of this subsection, we want to emphasize that lower treatment efficacies can actually be more beneficial for treatment than higher efficacies when switching at a given size before the nadir. In this case, not only is a higher efficacy unnecessary, but it is also less favourable than a lower-efficacy treatment. Therefore, the section is titled "Higher treatment efficacy is not necessarily beneficial for treatment". Note that this would not be true if switching at a given time (as opposed to a given tumour size). Moreover, we do not use the term "optimal treatment outcome" because the result only pertains to the before-nadir regime. Even if higher treatment efficacies lead to higher optimal P_E (across all switching sizes), it is good to know and emphasize that higher treatment efficacies are not always better for treatment since the optimal switching size may not be achievable in practice.

- **Fig3: I find it very inconvenient to go to another figure to find out what the (1), (2), (3), (4) labels stand for. I strongly suggest the authors use proper figure legends instead. I don't think it's a good practice for figure captions to cross-reference (especially if the caption refers to a future figure).**

  We have restructured our figures so as to avoid this issue.

- **It might be easier for the reader to split the two panels into two separate figures so that each plot could be letter-labelled. This would make in-text references to them a lot easier too.**

  We have significantly restructured the Results and redesigned the corresponding figures to make them self contained, such that each figure conveys a clear message.

- **I particularly like the results showing how carrying capacity doesn't noticeably affect the treatment effects. This potentially indicates ET could be applied to both primary and metastatic cancers, which would be good news for the clinical practitioners.**

  Thank you. We now mention this potential benefit in the Discussion (Section 4).

Reviewer 3

Comments to Authors (GENETICS-2024-307406)

In this manuscript, Patil et al. use a combination of analytical and simulation techniques in a theoretical investigation of "two-strike" chemotherapy for cancer. The Abstract and Introduction are well written and give a nice background to the problem, particularly its clinical relevance. The Methods section is concise, and in my opinion not detailed enough. Overall, having gone through the extensive

Appendices in addition to the main text, I believe the approach is generally sound, but I found the results difficult to follow. I think this issue could be addressed with substantial re-structuring to the text, clearer figure labels/captions, fixing some wording issues (with particular attention to defining terminology), and adding more detail to the main text. The Discussion reviewed some key results of the present study, but in my opinion, should have gone further in placing this study into the broader context of the literature on modelling evolutionary rescue and cancer therapies. I have provided more specific suggestions below.

Thank you for the insightful comments. We have made substantial revisions as detailed below.

- **In case of revisions, please could the authors add line numbers to their manuscript for ease of reference when commenting?**

  We have added line numbers.

Main comments:

1. **There is a lack of logical correspondence between results subsections and figures. Some results sections only refer to a single small sub-panel within a figure (e.g. section 3.5 only refers to "Figure 4A, second row, second panel") whereas other sub-panels are not specifically mentioned; references to panels within the same main figure are scattered across multiple sections; and the order in which figures are mentioned in the text does not always following the order in which they appear (e.g. parts of Fig. 4 and, briefly, Fig. 5 are already mentioned in section 3.3, but Fig. 3 is not mentioned until section 3.4, before jumping back to other parts of Fig. 4 in later sections). Some figures (2, 3) do not have a one-sentence title/description at the start of the caption describing the main message, while another (Fig. 4) has a generic title ("Effects of varying parameter values or initial conditions") that does not make the message clear. Figure 5 is mainly described in the Discussion, which is too late to be bringing up new results. Overall, I strongly encourage the authors to re-think the structure of the Results, so that each figure has a clear main message, and follows in a logical order corresponding to the main messages conveyed in the Results subsections. This might require re-thinking which plots/panels belong together in one figure, and which plots belong in main vs. supplementary. It might also call for reconsidering what really are the main findings of this study, and whether all the current sub-sections of the results are equally important, or if some points should be combined or shifted to supplementary.**

   We thank the reviewer for bringing this up. We have significantly restructured the Results and redesigned the corresponding figures to make them self contained, such that each figure conveys a clear message.

2. **The figures would benefit from clearer labels and descriptions. The plots tend to be quite complex, which is not necessarily a problem, but does call for careful exposition. I think this will already be greatly improved if the**

authors re-think the order and main message of each figure as suggested above. Some more specific suggestions for clarity on the plots follow - these are individually minor, but collectively would help a lot. (I have only referred to main figures here, but similar comments apply to the supplementary figures.)

Thank you for these suggestions. We have redesigned the figures and added new ones.

a) **Nq vs. q plots (Fig. 2C, Fig. 4A): These are clever but perhaps the hardest to get one's head around. I suggest moving the explanation of how to interpret the plots from the Methods (2.3) to the Results (at first appearance of such a plot) and perhaps expanding on the interpretation. In particular explain why some curves are "cut off".**

   We have expanded our explanation of the N_q vs q plots in the Methods section (Section 2.3) and added a figure in the appendix explaining their interpretation (Figure S1). Instead of moving the explanation to the first appearance of such a plot, we refer to the explanation at the beginning of the Results section (Section 3) and state that we use it as a metric throughout the section.

b) **Again in Nq vs. q plots: There are too many curves on the same plot to easily distinguish, and it is often difficult to match up which annotated label corresponds to which curve. I suggest reducing the number of curves per plot and/or using more distinct colours/dashes if possible, and using a legend or caption description instead of the annotated labels.**

   We have removed a subset of curves from the N_q vs q plots to make each curve more discernable. We have added legends instead of annotated labels.

c) **Fig. 2B: The axis labels are unclear, please be more specific: time = t and # of cells = N(t)? Also note that this panel is never specifically mentioned in the text, so consider whether it could be better used for illustrating a key point.**

   We moved this panel to Figure 1 to illustrate switching before and after the nadir.

d) **Heat maps (Fig. 3A, 4B) do not actually state what variable is represented by the colour. The captions say they show "high-PE regions" but I don't see how this is shown; the scale on the colour bar is up to thousands so it can't be a probability. I was also confused why the colour bar scale is not evenly spaced? Adjacent plots should also use the same scale to avoid misleading comparisons. Finally, it was unclear what an "optimal extinction probability contour" is - optimal over what parameters/constraints?**

   The heatmap legend is in units of total number of cells. It represents the difference between the maximum and minimum switching size needed (separately before the nadir and after) to achieve a high probability of extinction

($P_E \geq 0.8$). A value of 0 indicates that no striking strategy allows for $P_E \geq 0.8$. For optimal extinction probability, we are referring to the maximum probability of extinction that can be achieved by varying the switching size, over fixed parameters (where all parameters, except the variables being modified use default values).

We edited the figure legends and captions to explain the heatmaps and optimal extinction probability contours better. The colour scales are now evenly spaced. Using the same scale for adjacent plots in Figure 3 would be less informative because the values shown in the before nadir regime are much smaller than those shown in the after nadir regime, so the heatmap would be all black if we used the same scale. We now explicitly mention this in the figure caption and point out the different scales to avoid misleading comparisons.

e) **Fig. 3C: it is again very difficult to see which annotated label (coloured circled numbers) matches up with which curve, and having to flip back to a different figure (2A) to find some of the labels makes it hard to follow.**

We have significantly restructured our plots and have removed this multi-figure spanning legend.

f) **Fig. 5 was relatively easier to follow, but could be further improved: Would it help to indicate the time window corresponding to "high PE"? Please also clarify in the caption whether the population sizes are from ODE solutions (in absence of a second strike?).**

We have added some clarification to the caption regarding how we obtain the "solid line". Adding a high-P_E horizontal line makes the figure a bit cluttered, and it should be visually clear how large such a time-window is.

We have now moved this figure to the appendix and added some of its panels to relevant figures in the main text.

3. **Please make sure to clearly define key terms when they are first used, both in the main text and (where applicable) again in the appendices.**

We have now clearly defined all key terms.

a) **Pre-existing mutants (i.e. existing prior to any treatment, or prior to the second strike?) and de novo mutants (again, de novo during either treatment or only during the second?)**

Both pre-existing (or standing genetic variation) mutants and *de-novo* mutants are defined with respect to the beginning of the second treatment. So, rescue mutants present at switching time τ are pre-existing mutants and those generated during treatment 2 are *de-novo* mutants.

We now define these terms in the Methods section, Results section and Appendix.

b) **Resistant mutants versus rescue mutants; also "potential rescue mutants", as mentioned in Appendix A.1**

We changed our vocabulary as we felt that the expression resistant mutants could be misleading. We now explain our vocabulary in the second paragraph of Section 2.1. We write: "We use the following vocabulary. A potential rescue lineage is a lineage of cells with positive fitness for the whole future. That is, in our model, of $R_{1,2}$ cells, R_2 cells present at the switching time, and R_2 cells arising after the switching time. While R_1 cells have a positive fitness during treatment 1, they are counterselected during treatment 2, hence cannot rescue the population. We include in potential rescue lineages those that get extinct. Founders of potential rescue lineages are called potential rescue mutants. Potential rescue lineages that escape stochastic extinction and cause evolutionary rescue are called rescue lineages, and their founders rescue mutants. Rescue mutants already present at the switching time τ are called *pre-existing* rescue mutants, or *standing genetic variants*. Those appearing after are called *de-novo* rescue mutants."

We also recall this vocabulary in the Appendix.

c) **Establishment: is this taken equivalent to non-extinction in a branching process model, as opposed to reaching a finite threshold population size?**

   Yes, we are using as an approximation establishment to be non-extinction of a single cell following a branching process model (with no competition). We define "establishment" at its first mention in the Methods section and in Appendix A.5.

d) **Progression and persistence (first mentioned in section 2.2 but never defined in the main text)**

   Progression and persistence are both simulation outcomes. Progression is when the total population at the end of the simulation is greater than initial total initial population. Persistence is when the population does not go extinct but is smaller than the initial population.

   We now clearly define these terms in Section 2.2.

e) **Extinction therapy**

   We believe that the term "extinction therapy" was coined by Gatenby & Brown [11, 12]. We now use this term only once, where we note that it is an alternative name for what we call multi-strike therapy.

f) **Conventional treatment/therapy - this term is used in the Discussion without explanation**

   We refer to several conventional treatment strategies. Conventional single-strike therapy is the use of a single drug, while conventional sequential therapy is when the subsequent treatment is only given after evidence of relapse, not guided by eco-evolutionary principles. We now clearly mention in each instance which conventional treatment we are referring to.

4. **The Methods section is missing some important details:**

a) **It is not sufficiently clear how $R_{1,2}$ is modelled. Fig. 1 (Part 1) shows an ODE for $R_{1,2}$ but I gather this is never actually used? This comes up again in Appendix A.1 and again it is unclear how exactly R1,2 is modelled.**

$R_{1,2}$ is modelled as is (following the ODE). There is no explicit $R_{1,2}$ term in Eq 2 because we assume $R_{1,2}(0) = 0$, and only the number of $R_{1,2}$ cells generated by mutation (as opposed to reproduction of former $R_{1,2}$ cells) is required to calculate the extinction probability. We now clarify how we model the number of $R_{1,2}$ rescue mutants in the Methods section and in the Appendix.

b) **During the second treatment (cf. Fig. 1, Part 2), are ODEs also used to compute $S(t)$, $R_1(t)$, $R_2(t)$ as they appear in the integrals in the P_E equations? This also needs to be stated in Appendix A.1.**

Yes, the dynamics of S and R_1 populations in E_2 are also modelled using ODEs. These ODEs are mentioned in Appendix A.3 (Eq 29 and 30). We now refer to this in the Methods section as well as in Appendix A.1.

c) **Equation 1-2 for extinction probability is arguably the most important equation in the manuscript and would benefit from a more detailed explanation. It would be helpful to identify what contribution each term in line 2 represents. (This is especially relevant later, e.g. in section 3.1 where you refer to particular terms.) Also clarify, to which resistant strain(s) is the establishment probability relevant (only those resistant to drug 2?). State in words what the mentioned parameters b, d, c represent.**

We clarify that the establishment probability is the same across time and for all resistant cell types. We state that the parameters b, d and c are the intrinsic birth, death rate, and cost of resistance respectively.

We add the following explanation of Eq 2 in the Methods section (Section 2.1) and in Appendix A.3:

The quantity in the exponent represents the total expected number of rescue mutants generated if we switch at time τ. The first term represents the number of established R_2 cells at the switching time. The next two terms ($\pi_e(\int_0^\tau \mu_2 R_1(t)\,dt + \int_0^\tau \mu_1 R_2(t)\,dt)$) represent the total expected number of established $R_{1,2}$ mutants generated by mutation from either R_1 or R_2 cells during E_1. The last two terms ($\pi_e \mu_2(\int_0^\tau S(t)\,dt + \int_0^\tau R_1(t)\,dt)$) compute the number of established R_2 and $R_{1,2}$ cells generated by mutation from S and R_1 cells during E_2.

d) **Consider moving more material from Appendix A.2 into the main Methods; a lot of this seems important for understanding the approach.**

We respectfully disagree with the suggestion. Especially after explaining each term appearing in the probability of extinction according to your comment (c), we feel that all that is strictly necessary to follow the paper is already explained in the Methods. The interested reader may always refer to Appendix A.2 and A.3 for a more formal statement of the assumptions

used to derive these formulas. We now refer to both the appendices in the Section 2.1.

e) **It could be more clearly emphasised already in Methods that you will consider two possible switching criteria: based on population size, or based on time. Throughout the Methods and Results, this distinction was not always obvious but is a critical point for understanding some of the parameter effects that you describe.**

We attempt to make this distinction clearer in the Methods and Results sections. We replace the term “switching point” with either “switching size” or “switching time”, depending on the specific criteria we are analysing.

f) **More detail on the stochastic simulation methods should be included in the main text. In particular, it is unclear what you mean by “100 independent runs, each with several switching points” in the caption of Fig. 2, or “100 paired simulations with different random seeds” in the caption of Fig. 3. It is also unclear whether all parts of the model were fully stochastically simulated, or whether some parts still rely on the derived equations. In particular, Appendix A.6.3 seems to suggest that extinction probabilities are still “calculated” in the simulation, which does not seem like a sufficient check on the quality of the mathematical approximations.**

For each switching size and parameter combination, 100 independent replicates of the Gillespie algorithm are run. The extinction probability is only “calculated” in that we estimate it via $\frac{\text{number of runs in which } \exists t, N(t)=0}{\text{total number of runs}}$.

We now add the following sentence to the relevant figure captions: “Extinction probabilities from the simulations are estimated for each switching size as the proportion of extinction outcomes in 100 independent runs.” We removed the term “random seeds” from the main text for simplification. In Appendix A.7.3, we expanded the explanation of the computation of the extinction probability and how the independent simulations are implemented using random seeds.

5. **Some of the results are quite complex and subtle, and I often found the authors’ verbal explanations hard to parse. Several wording issues came up repeatedly:**

a) **I repeatedly struggled to see what the authors meant by “high-PE region”: region in what space? (Population size?) Could this “region” be indicated on the relevant plots?**

We now call this quantity the “range of high P_E switching sizes”, defined at the beginning of Section 3.

b) **When the term “initial” is used, it isn’t always clear whether it refers to the start of treatment 1, or the start of treatment 2.**

Whenever the term “initial” is used, it refers to the beginning of the first treatment. We now state this explicitly on several mentions of the word so that the distinction is clear.

c) **The term "benefit" is used loosely and it isn't always clear what kind of benefit – to the patient or to the tumour? (This was particularly confusing when phrased as "the benefit of a cost of resistance".)**

   We have either replaced the term "benefit" or clarified that it is "beneficial for treatment".

d) **Likewise, the term "optimal" is not always clear – optimal under what criteria, with respect to what parameters?**

   The term "optimal" is always used to refer to the highest extinction probability. So the optimal $N(\tau)$ is the switching size leading to the highest extinction probability. We have clarified this in the beginning of the Results section, at the first couple of mentions of "optimal switching size".

It will also generally be helpful to add definitions of key terms (main comment 2 above) and ensure it is clear from the outset when the results refer to a fixed-size versus fixed- time switching criterion (cf. main comment 3d above). (Sometimes, this point was mentioned but only very late, e.g. bottom of p.7 after presenting results that crucially rely on this assumption.) I have highlighted other, specific wording issues in "Other minor comments" below. I have two other general suggestions the authors could consider to help with exposition of their results:

Thank you, we have added definitions of key terms and mentioned the switching criteria wherever necessary.

e) **Showing more plots simply of the population dynamics (S, R1, R2, R1,2 over time), under different parameter regimes, could be extremely helpful in building the reader's intuition and illustrating some of the finer points the authors describe - e.g. trade-offs associated with minimising the S population versus minimising the R1 population at the time of the treatment switch.**

   After experimenting with different approaches, we have added to Figure 1 a plot showing the total population dynamics for two switching sizes. We opted not to show the subpopulation dynamics as these are difficult to depict clearly.

f) **Just from reading the main text, it would be easy to miss the extent of analytical results derived in the Appendices. Importantly however, these results also come with a few caveats (e.g. derived for a model with no competition; certain approximations are only valid under certain parameter conditions). I think the authors could make better use of their extensive analytical work by making it clearer in the main text what they have done: e.g. a brief summary of the approach/assumptions in Appendix 1 could be included in the main Methods. They could also consider putting a couple of key equations (e.g. maybe for Nmin, RM(t)) into the main Results.**

   Thank you for this suggestion. We now summarize in the Methods the main analytical results of Appendix A.1.

6. **I felt that several important points were missing from the Discussion:**

   (a) **The authors mentioned various multi-drug treatment strategies in the Introduction, and I immediately wondered why two-strike therapy (sequential use of two drugs) would ever be theoretically preferable to simultaneous use of both drugs from the start? However, this point was never addressed in the Discussion. I imagine there would be clinical arguments e.g. around drug toxicity, but this aspect is neglected in the model. Wouldn't simultaneous treatment be optimal for maximizing extinction probability? This question has been widely debated in the drug resistance literature and I strongly suggest it should be touched upon here.**

   We agree that it is important to touch upon the subject of combination therapy and have therefore added the following to the Discussion: "When tolerable, combination therapy (that is, applying both treatments simultaneously) should also be considered. Whether it is better to apply drugs together or in sequence depends on several factors including the doses that can be administered in each case, the shape of the dose-response curves, the extent of synergism, and the degree of cross-resistance [13, 14, 15, 16]. A comprehensive comparison of these alternative strategies using our mathematical methods is beyond the scope of the current study and remains an important topic for future research (but see Appendix A.6)."

   In the new Appendix A.6 we present a simple model illustrating why combination therapy is not necessarily better than a two-strike strategy.

   (b) **In general, the Discussion does not go far enough to place the present study in the context of previous work modelling the impact of treatment strategies on evolution of drug resistance in cancer. The authors state that extinction therapy has been modelled previously (ref. 16), and claim that their work "yields clearer explanations and more general results" but it is unclear how. Meanwhile, they have not cited some other studies that also modelled stochastic extinction in cancer treatment, e.g. Hansen & Read, 2020 (doi:10.1111/eva.12994). There is a brief discussion of alternative treatment strategies on p.13 but this should be expanded; please also explain/remind the reader what "conventional therapy", "extinction therapy", "adaptive therapy" mean.**

   We now clarify in the Discussion that our model "yields clearer explanations and more general results than previous computational modelling of two-strike therapy [17], in that we perform a more systematic analysis of the parameter space and we obtain extensive analytical results." We now discuss alternative treatment strategies in more detail in the Discussion subsection "When should two-strike therapy be used?". We have added more references to previous evolutionary rescue studies and we now cite Hansen & Read, 2020. We define all terms when first used.

   (c) **The authors only briefly mention a crucial point: that realistic "tumour detection thresholds are much higher than the initial population**

sizes we consider". This warrants a much deeper discussion: How should switching strategies be designed in practice? Given that the modelled population sizes are always below detection, how does the present study then inform realistic switching strategies? How can switching after the nadir be considered the "safer" strategy, if the nadir cannot be observed?

Establishing how switching strategies should be designed in practice is beyond the scope of our study. Nevertheless, we have added a subsection to the Discussion entitled "Designing practical two-strike strategies" that outlines ideas for future investigation.

(d) **The role of mutation rate, as found in the present study (section 3.6), is unsurprising. The result that mutations increase the chance of evolutionary rescue is essentially built into the model, since the only mutations considered are those that lead to treatment resistance. This effect of mutation rate is well known in the evolutionary rescue literature (reviewed e.g. the manuscript's ref. 10), and should be placed in that context if highlighted at all. Given this model limitation, it seems inappropriate to suggest that the model has any bearing on "mutagenic therapies", which crucially rely on the accumulation of deleterious mutations, which are not modelled here. If the authors go down this route, they should provide a clinical reference for mutagenic therapies, and point to existing model(s) in the evolutionary rescue literature that do explore deleterious mutations alongside rescue mutations (I am aware of only one: Anciaux et al. 2019, doi:10.1111/evo.13771).**

Thank you. We have removed this subsection from the Results.

(e) **The result that extinction probability increases with turnover (section 3.7) has also been found by several other studies, besides the one (ref. 21) cited in the Discussion: see e.g. Raatz & Traulsen 2023 (doi:10.1093/evolut/qpad042) and Czuppon et al. 2023 (doi:10.1371/journal.pcbi.1011364).**

We have added an additional reference to the Discussion when discussing turnover.

Other minor comments on main text:

1. **Consider whether a more specific title would be more suitable - there are other papers addressing evolutionary rescue in cancer; perhaps highlight the specific focus on two-strike therapy?**

   We agree. We have changed the title to "Preventing evolutionary rescue in cancer using two-strike therapy".

2. **Start of Methods and later: I found it strange to refer to a "deterministic analytical model" where extinction probabilities are being calculated, fundamentally indicating an underlying stochastic process. I suggest it would be more suitable just to call this an "analytical approach" (to what is, effec-**

tively, a hybrid deterministic/stochastic model), in contrast to a "(stochastic) simulation approach".

We have removed reference to "deterministic".

3. **It may be worth explicitly mentioning the assumption that resistance is "complete", i.e. resistant strains are not affected at all by the corresponding drug.**

   In Section 2, we now explain that the resistant cells are completely resistant.

4. **Figure 1 (Part 2) seems to suggest that the population is at equilibrium prior to treatment. It would be helpful to state explicitly in the Methods that equilibrium is not necessarily assumed (neither demographic equilibrium, nor population genetic equilibrium, i.e. mutation-selection balance).**

   We now state this explicitly in Section 2.4.

5. **Figure 1: The caption describes the solid curve in the Part 2 plot but not the dashed curve. In general, the description of Part 2 is brief/incomplete in the caption.**

   We have edited and expanded the caption to describe Figure 1 better.

6. **Section 2.3: Please clarify what you mean by "in absence of a second strike": In this case do you assume that treatment with drug 1 continues indefinitely?**

   Yes. We define $N_{\min}$ in the case when we do not switch to the second treatment, so treatment 1 continues. We now clarify this in Section 2.3.

7. **Section 2.3: "Any given switching point N(t) is reached twice…" – this was confusing wording. A given population size N(t) would be reached twice if treatment 1 simply continued, but a switch to treatment 2 only occurs once. It might help to emphasise that you are using population size as a switching rule here.**

   We have edited this sentence and added an illustration.

8. **Last line before Section 3.1: "the maximum population size threshold that must be crossed" – this is unclear wording (crossed in which direction?) and "must be crossed" could simply be deleted for a clearer sentence.**

   Thank you. We have edited this sentence to make it clearer. Now, it says: "As defined in Section 2, N_q is the maximum switching size to achieve an extinction probability greater than or equal to q."

9. **Section 3.1: "the decay in the R2 population relative to…" – I'm struggling to parse this sentence or see how it explains the optimal N(t).**

   We have added an explanation and rephrased the sentence.

10. **Taken at face value, the title of section 3.2 ("It is better to implement the second strike after the nadir than before") directly contradicts the statement**

above within section 3.1 that "the optimal switching point may... lie slightly before or after Nmin". Please clarify.

The optimal switching point may lie before or after the nadir, but in Section 3.2, we look at the range of high-P_E switching sizes (the so called window of opportunity). The window of opportunity extends further in the after-nadir regime, that is, there is a larger range of high-P_E switching sizes after the nadir than before. Therefore, our result states that it is better to implement the second strike after the nadir than before. We have edited Section 3.1 to clarify this distinction.

11. **Section 3.2 (p.6): The explanation of the pre-existing R2 population is hard to follow. It sounds like the hypothesis proposed would be sensitive to R2(0); but is the default value realistic? The argument that delaying switching results in a smaller rescue population during the second treatment is hard to infer from the referenced figure, which shows varying R2(0), not R2(t).**

    Our hypothesis is that the difference in extinction probabilities between the before and after nadir regimes depends on the initial R_2 population. To illustrate this, we compare extinction probabilities with and without an initial R_2 population in Figure 2. To provide the reasoning for our hypothesis, we refer to Appendix A.1.2.4. We agree that the default value of the initial resistant population is large (compared to the reference case derived in the appendix). However, this allows us to study the worst-case scenario in which the treatment history leads to a large resistant population, giving us conservative bounds on the extinction probability. We also consider and state the consequences of choosing a large initial R_2 population, and compare it (wherever relevant) to the case with $R_2(0) = 0$.

12. **Section 3.3, first paragraph on p.7: I found this explanation very hard to follow. Not all of the referenced figures/curves within the plots clearly illustrate or agree with the statements made. The meaning of "optimal" extinction probability is unclear. It would also help to be clearer from the outset that the switching criterion here is a certain population size, not a certain time.**

    We have now restructured the Results section so that each result is illustrated by a corresponding figure (or figure panel). We also edited the contents of this section and we hope that it is clearer.

13. **Section 3.3, last paragraph: "intermediate" treatment dose is unclear – there are only two doses illustrated.**

    We replace "intermediate" by "low" throughout the main text. The low and high treatment efficacies are clearly defined.

14. **Section 3.4: Most of this section deals with a switch implemented before the nadir, where it turns out that a higher dose is not always best. However, earlier in the Results (section 3.2) it was already stated that a switch after the nadir is better. So why not just stick with switching after the nadir, then the highest possible dose is unambiguously best? (In other words, why**

do we still care about before-nadir results at this stage? This is interesting theoretically but the emphasis seems misplaced.)

There are a couple of motivations to study the effect of changing treatment efficacies in the before-nadir regime, even if switching after the nadir is optimal. Lowering treatment doses could be important when considering treatment toxicity. It is useful to know if and under what conditions is it possible (or even favourable in our case) to lower the efficacy of one of the two treatments. Moreover, it may not always be possible in practice to give the first treatment for very long. Another reason we explain this result and the intuition behind it in detail is that it depends on the way the treatment is administered. This result is observed if we switch at a fixed size, and not when we switch at a fixed time. It is important to know when and how the switching criteria affects the choice of treatment.

We have edited the paragraph to explain our motivation better, and we also discuss it in Section 4.

15. **Section 3.7 (second paragraph): the "good" region is too vaguely defined.**

    We have edited the first paragraph of Section 3.7 to clearly define the "good" region.

16. **Section 3.7 (last paragraph): "turnover may relate to the establishment probability"? Can the authors be more definitive?**

    We have replaced this sentence by "Another effect of turnover relates to the establishment probability of resistant mutants."

17. **Section 3.8: I found this section hard to follow. The referenced figure does not clearly illustrate the effects described in the text; e.g. K = 10 N(0) is not plotted. I don't follow why lower K results in "a lower decay rate for S cells and a higher growth rate for R1 cells". The phrase "both factors are present" is also unclear.**

    We have edited this section and the corresponding figure to make the message clearer. We plot the curve for $K = 10N(0)$ as well as the curve for $K = \infty$ in Figure 5 to show that the effect of changing the carrying capacity saturates. The sentence "a lower decay rate for S cells and a higher growth rate for R1 cells" was a typo and has been fixed.

18. **Discussion, section "When do we get the optimal extinction probabilities?", paragraph 2: What do you mean by an extinction "rate"? How does it relate to the "high-PE region"?**

    We edited this sentence and replaced extinction rate with extinction probability.

    Extinction probability is used to define the "high-P_E regions" (now called the "range of high-P_E switching sizes"). Switching sizes that lead to extinction probability greater than or equal to 0.8 are the high-P_E switching sizes. In this particular paragraph, we use the range of high-P_E switching sizes near the nadir to show that it is better to switch after the nadir than before, because the range is larger for after-nadir switching sizes.

19. **Discussion, 2nd-last paragraph on p.12: "the effect of changing the mutation rate might be different..." – I suggest either explaining in what way it will be different, or removing this sentence. The statement that "our results are subject to certain methodological assumptions and limitations" is extremely generic and could be applied to any modelling study, so again, either elaborate (highlighting the most important assumptions) or delete.**

    Thank you for the suggestions. We edited the paragraph to explain how our modelling assumptions can affect extinction probabilities. We remove the statement "our results are subject to certain methodological assumptions and limitations".

20. **Conclusion and future directions: "two-strike therapy...could plausibly increase cancer cure rates" - increase compared to what?**

    Compared to conventional single-strike or sequential treatment strategies. We edited the sentence to clarify this.

Other minor comments on appendices:

1. **General comment: It would be helpful to number more of the equations and refer back to them specifically whenever they are re-used/substituted in. (E.g. above Eq. 12, which expressions for Ri(t) are subbed in?)**

   Thank you for this advice. We agree. We have numbered more equations, introduced a couple of new ones, and clarified which expressions for $R_i(t)$ are used by referring to the corresponding equations.

2. **General comment: There is a lot of notation in the appendices and I would have found it helpful to have another table summarising all the symbols and their definitions.**

   In Appendix A.1, we now provide a table summarizing symbols used only in this Appendix and their definitions.

3. **General comment: Please ensure that wherever an approximation is made, the assumption/condition for it to hold is stated. This is often, but not always, done. Where possible, it would also help build intuition for the results to explain the interpretation of conditions required for approximations to hold. E.g. top of p.17,** $\exp(-(\delta_1 - \gamma_S)\tau) \ll 1$ **means the S population declines fast under treatment 1.**

   Thank you for pointing that out. We have clarified that $\exp(-(\delta_1 - \gamma_S)\tau) = S(\tau)/S(0)$, so assuming that this quantity is much lower than 1 is not really assuming that the S population declines quickly, but rather that it already declined a lot. We have also clarified when the approximation for $R_1(t)$ in former Eq. (6) is valid.

4. **Appendix A.1: Remind the reader that time 0 is the start of treatment 1 and define S0 := S(0) (presumably)**

   We now clarify that $S_0 = S(0)$.

5. **A.1.1, Eq. 11: The final approximation represents mutation-selection balance; I think this would be worth pointing out.**

   Indeed. We now mention mutation-selection balance.

6. **A.1.1, just after Eq. 12: "the R2 population is fully hidden…" – I don't understand what you mean here. Also when you say "This allows us to study…" what is "This"?**

   First, we suppressed the expression "the R_2 population is fully hidden". We meant to point out that though there is no explicit term in R_2 in the right-hand side of former Eq. (12) (now Eq. 19), R_2 rescue mutants are implicitly accounted for as part of the term $\alpha_S S$. This is because in the reference case of Eq. (18), $R_2(t) \approx \frac{\mu_2}{c_2} S(t)$ and is thus a function of $S(t)$. However, we now feel that pointing this out is not really necessary and we removed the sentence. The sentence "This allows us to study..." was indeed confusing due to a kind of typo (the sentence that should have been before it had been commented away by mistake). "This" referred to "assuming larger initial resistant populations as we do in our simulations". This is now corrected.

7. **Throughout: "Eq. 11" and "the reference case" seem to be used interchangeably; it would be helpful always to state "the reference case (Eq. 11)" instead of just one or the other (inconsistently).**

   Good point. Former Eq. 11 is now Eq. 18, and we now always refer to "the reference case (18)" when mentioning the reference case (except immediately after deriving this equation where we just refer to (18)).

8. **A.1.2.1: "Around the nadir, most tumour cells are either S cells or R1 cells" – this should be true for the reference case but not for arbitrary R2(0).**

   Thank you for this remark. Our writing was misleading. Indeed, it might be clearer to say that, for reasons described below, we expect most cells to be either S cells or R_1 cells not only close to the nadir, but throughout the administration of treatment 1. There are exceptions to this rule. For instance, if there is a negative cost of resistance, so that R_2 cells are fitter than S cells, and moreover R_2 cells appeared early in the history of the tumour. In that case, at the beginning of treatment (time 0), the tumour could consists mostly of R_2 cells, and not of S cells. A similar issue arises if $R_{1,2}$ cells are fitter than R_1 cells and appear early. Such cases are however very far not only from the reference case of the former Eq. (11) (Now Eq. (18)), but also from what we assume in our simulations.

   Unless R_2 cells are fitter than S cells before treatment begins, at time 0, R_2 cells should be much rarer than S cells: $R_2(0) \ll S(0)$. Unless R_2 cells are fitter than S cells during the administration of treatment 1, this will continue to be the case throughout the administration of treatment 1 (since the R_2 population then decays quicker than the S population), so we still have $R_2(t) \ll S_2(t)$. For similar reasons, we expect that $R_{1,2}(t) \ll R_1(t)$ during the whole administration of treatment 1. Therefore, during treatment 1, most tumours cells should be either S cells or R_1 cells.

We now write: "Unless we assume a negative resistance cost, we expect that at time 0 and throughout the administration of treatment 1, $R_2(t) \ll S(t)$ and $R_{1,2}(t) \ll R_1(t)$, so that most tumours cells are either S cells or R_1 cells. Thus …"

9. **A.1.2.4, 2nd line: What are "relevant" switching times?**

   By "relevant" switching times we meant those that correspond to intuitively reasonable treatments, that is, such that $N(\tau) \ll N(0)$. We rewrote this paragraph.

10. **A.1.2.4, 3rd paragraph: "if they hold in an appropriate average sense" – what do you mean by this? Simply that the inequality above holds?**

    We indeed simply meant that this equality holds, but we rewrote the whole passage as the logic behind this condition became clearer to us while reworking on it.

11. **A.1.2.4, 6th paragraph ("We conclude…"): Most of this paragraph seems like main text material in my opinion. However, the robustness of the result that "tumour size and the number of expected rescue mutants tends to decrease relatively quickly before the nadir, and then to rebound more slowly" should also be illustrated, and the mathematical result in footnote 6 should actually be shown.**

    We rephrased this passage, to clarify the reason why tumour size (and therefore the expression $\alpha_S S + \alpha_1 R_1$ and the number of expected rescue mutants in the reference case (18)) tends to decrease quickly before the nadir and then to rebound more slowly. This is because we assume that during treatment 1, the decay rate of S cells is larger than the growth-rate of R_1 cells (that is: $\delta_1 - \gamma_S > \gamma_1$). We now mention that this conclusion would be reversed for parameter values such that $\delta_1 - \gamma_S < \gamma_1$. We also mention that this result is now illustrated by Figure S3.

    Concerning footnote 6, we added the following proof: Indeed, with the approximation $\dot{R}_1 = \gamma_1 R_1$ close to the optimal switching time t_{opt}, and letting $\alpha = \delta_1 - \gamma_S$, $\beta = \gamma_1$, we get that $\alpha\alpha_S S(t_{\text{opt}}) = \beta\alpha_1 R_1(t_{\text{opt}})$, and that

    $$RM(t_{\text{opt}} + \Delta) = -S(t_{\text{opt}})e^{-\alpha\Delta} + R_1(t_{\text{opt}})e^{\beta\Delta} = c\left[-\frac{e^{-\alpha\Delta}}{\alpha} + \frac{e^{\beta\Delta}}{\beta}\right]$$

    where $c = \alpha\alpha_S S(t_{\text{opt}}) = \beta\alpha_1 R_1(t_{\text{opt}})$. It follows that for $\Delta > 0$,

    $$\begin{aligned} RM(t_{\text{opt}} + \Delta) < RM(t_{\text{opt}} - \Delta) \quad &\Leftrightarrow \quad 2\frac{\sinh(\beta\Delta)}{\beta} < 2\frac{\sinh(\alpha\Delta)}{\alpha} \\ &\Leftrightarrow \quad \frac{\sinh(\beta\Delta)}{\beta\Delta} < \frac{\sinh(\alpha\Delta)}{\alpha\Delta} \Leftrightarrow \beta < \alpha \end{aligned}$$

    where $\sinh(x) = \frac{e^x - e^{-x}}{2}$ is the hyperbolic sine function, and the last equivalence follows from the fact that $x \to \frac{\sinh(x)}{x}$ is increasing on $(0, +\infty)$. Since $\alpha = \delta_1 - \gamma_S > \gamma_1 = \beta$, the result follows.

12. **A.1.2.5, 1st sentence: "not taking into account treatment toxicity" – this seems like a very important caveat that should have been stated somewhere in the main text!**

    We added the following sentence at the beginning of Appendix A.1.2.5: "Treatment toxicity is a major concern in cancer therapy, and may justify to use relatively mild doses. Here, we discuss whether there might be other motivations to reduce the efficacy of treatment 1."

    In Section 2, we state the caveat that we do not consider treatment toxicity, and we mention it as a motivation to consider lower treatment doses in Section 3.3.

13. **A.1.2.5, 3rd paragraph: "a lower value of d1 leads to... a higher growth rate of the resistant populations" – do you mean specifically R2?**

    A lower efficacy of treatment 1 indeed directly leads to a higher growth-rate of the R_2 population (in this case a slower decay). But it also leads to a larger sensitive population, hence to more mutations from S to R_1 and a slightly larger R_1 population. The increase in the R_1 and R_2 populations then leads to a slightly larger $R_{1,2}$ population. So directly or indirectly, a lower efficacy of treatment 1 leads to a higher growth-rate of all resistant populations. We now explain this more carefully in the first paragraph and then build on this explanation in the third one.

14. **A.1.2.5: It was unclear what the conclusion of this section was.**

    We added a concluding sentence: "Thus, for a relatively large value of the R_2 population, as in our simulations, we expect that the main effect of a decrease in the efficacy of treatment 1 *when switching at a given size and before the nadir* is to decrease the number of SGV R_2 rescue mutants. This explains why a lower efficacy of treatment 1 may then be paradoxically beneficial (Figure 3(A), left, and comparison of Figures 2(A), left, and 3(B), right)."

15. **A.1.2.6: Be careful about defining "benefits" – there is particularly confusing language in this section around "benefit of costs" and "all costs of resistance are beneficial".**

    We rephrased part of this section to make the language less confusing. We write ”A key impact” instead of a ”A key benefit”, and later clarify that "beneficial for treatment" means a higher probability of tumour extinction.

16. **A.1.2.6, 2nd paragraph: costs "do not impact the sensitive population" – when neglecting competition?**

    Good point. If sensitive cells compete with resistant cells, then a decrease in the number of resistant cells would lead to an increase in the number of sensitive cells. However, since there are initially way more sensitive cells than resistant cells, this would have a very small impact, in the sense of multiplying the number of sensitive cells by a factor very close to 1. During the course of treatment, competition is reduced (at least in the model of the main text, since a lower tumour size leads to less competition). Thus the effect of a reduction of the

resistant population on the dynamics of sensitive cells is then even lower. We added a footnote: “In a model with competition between tumour cells (such as in the main text), resistance costs, by reducing the number of resistant cells, would slightly increase the number of sensitive cells, but we still expect resistance costs to lead to larger probabilities of extinction for a given switching time, as observed in our simulations (Figure S3, compare columns 1 and 2, see also Figure S4).”

17. **A.1.2.6, 3rd paragraph: “they do not significantly affect switching time” – how do we know this?**

    During the administration of treatment 1, $R_2 \ll S$ and $R_{1,2} \ll R_1$, so $N \approx R_1 + S$. Moreover, the dynamics of R_1 and S is not affected at all by resistance costs affecting R_2 and $R_{1,2}$ cells in the absence of competition (as in Appendix 1), and would be only very slightly affected during treatment 1 in the presence of competition (as in the main text), since $N \approx R_1 + S$. Therefore, such resistance costs would hardly affect the time at which the tumour reaches the switching size. We added a footnote explaining this.

18. **P.21, footnote 7: “the net effect should be estimated more carefully” – it is unclear whether you have actually done this somewhere? (It seems important, since the main text model does include competition.)**

    Thank you for this comment, which prompted us to explore this issue in greater details. We added the following text: ”For example, with a lower δ_1, the time before R_1 can grow at its competition-free rate increases, meaning that at a given time, there would be fewer R_1 cells, hence fewer R_{12} mutants arising from R_1 cells, whether before or after switching to treatment 2. So, if switching at a large time, when S cells and SGV R_2 mutants are insignificant, a lower value of δ_1 could increase the probability of extinction. This is observed comparing the probability of extinction between $\delta_1 = 1.2$ and $\delta_1 = 2.0$ at otherwise default parameter values when switching at $\tau = 20$ (see Figure S3). Similar issues are studied by [8] and [9], e.g., pp. E31-E32, and in the literature on tumour containment [10].”

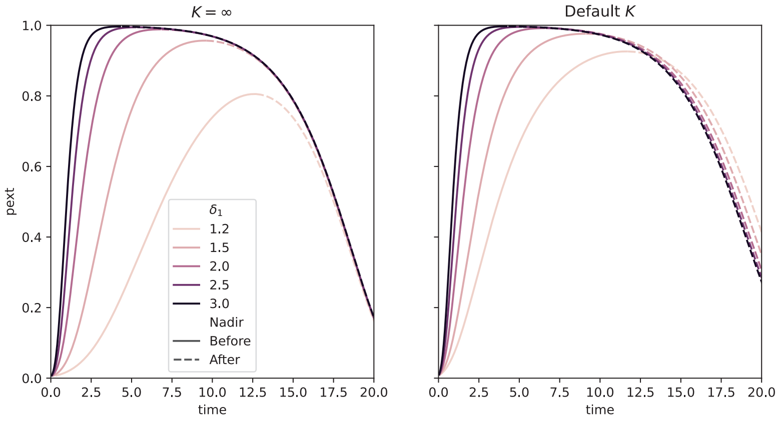

Figure 4: Probability of extinction as a function of switching size according to the analytical model, either without competition (left) or with competition (right). Treatment 1 is varied within $\delta_1 \in [1.2, 3]$

We have added Figure S5 (reproduced here as Figure 4) to illustrate this point. The left panel confirms that in the absence of competition, this subtlety disappears: when switching at a given time, a high efficacy of treatment 1 now always increases the probability of tumour extinction.

19. **A.1.2.6, final sentence: "We conclude that... a larger cost of resistance might..." – so was this effect indeed observed in the numerical results?**

    In the main text, the cost of resistance c is the same for all types. So when decreasing this cost of resistance, the potential decrease of the probability of extinction linked to the increase in c_1 is at least in part compensated by the increase of the probability of extinction linked to the increase in c_2 and $c_{1,2}$. Yet, we still observed situations where increasing the common cost of resistance c paradoxically decreased the probability of extinction, as discussed in Section 3.4. We now refer to this Section.

    Note also that we suppressed the paragraph on the impact of a cost of resistance when switching at a given size before the nadir. This is for consistency with the main text (Section 3.4) and because the situation when switching before the nadir is too complex to reach definite, easy-to-understand conclusions.

20. **Appendix A.2: Haldane's $2s$ is a poor approximation of establishment probability if s is large, as will often be the case for drug resistant mutants under effective drug treatment.**

    We have added this disclaimer.

21. **A.2: I suggest avoiding the term "stress", particularly "degree of stress", since this could be confused with other biological meanings of stress (e.g.**

related to physiological responses of cells). I suggest simply saying "drug treatment/dose".

We have rephrased.

22. **A.2, 3rd paragraph: "we make the assumption that the probability of establishment of a resistant mutant is constant throughout"... i.e. over time, within the second treatment phase? Please clarify and state why it is reasonable to assume this.**

    We add clarification and explain why it is okay to make this assumption.

23. **A.2, top of p.23: "t_{ext} is the time at which the population would go extinct if it is not rescued" - isn't this a random variable? (Or in the ODE model, the population never technically goes extinct.) Wouldn't it be easier to integrate to time infinity?**

    If population dynamics are deterministic then t_{ext} would in fact be deterministic (and in our model would be ∞ as a 0 population is never reached assuming we start with a non-zero population). In any case, we replaced t_{ext} with ∞ as it would make no difference and is likely clearer.

24. **A.2, top of p.23: I'm not following the wording here - what are "the first and second terms"? What do you mean by "a population that only becomes resistant to the stress induced at $t = 0$", or "a rescue population generated by mutation from N"?**

    We have modified the sentence to make it clearer.

25. **A.3, 1st paragraph: "This [pre-existing variants] gives us a good estimate of the rescue probability due to R2 mutants." Why can de novo R_2 mutations during treatment 2 be neglected?**

    This is a typo. Thank you for pointing it out! We have now edited the sentence: "Thus, we can obtain a good estimate of the rescue probability due to pre-existing R_2 mutants".

26. **A.3, below Eq. 15: "integrating them until the population size is equal to one" - which population? Why equal to one?**

    We numerically solve the last two integrals in Eq 28 until the size of the corresponding population is equal to 1. For example, we numerically integrate $\int_{\tau}^{\infty} S(t)dt$ until $S(t) = 1$. We do this to make the computation of the integral faster and easier. Since the number of S and R_1 cells never technically goes to zero in the solution of the system of ODEs (the asymptote is 0), we must set a threshold to define extinction. The threshold of 1 is chosen arbitrarily. Essentially, we chose a time to integrate until to approximate integrating to infinity.

    We rephrased the explanation in Section A.3 to make it clearer.

27. **A.3, last paragraph: "solve" the equations would be a clearer word choice than "evolve"**

    Done.

28. **A.4, top of p.24: "This assumption is generally valid for the established rescue lineages" - explain why.**

    Consider the dynamics of the established lineages, which are formed from $R_{1,2}$ rescue mutants during E_1 and $R_{1,2}/R_2$ rescue mutants during E_2. These lineages do not birth, and due to them being established do not die. Rather they only come about by immigration at rate $\pi_e \cdot r(t)$. $r(t)$ is the rate of formation of the relevant pre-rescue mutant: $\mu_1 \cdot R_2(t) + \mu_2 \cdot R_1(t)$ for $R_{1,2}$ and $\mu_2 \cdot S(t)$ for R_2 cells. Without loss of generality, we focus on the de novo R_2 lineages. Using the deterministic dynamics for the rate $r(t)$ is approximate, and it should hold when the generating population, S, is large. Otherwise, if S is small and hence stochasticity becomes important, note that whether or not we use deterministic or stochastic dynamics the rate $r(t)$ will be negligeable at reasonable parameters (as μ_2 is small). Denoting as L the random variable counting the number of established lineages:

    $$\frac{dP_L(n;t)}{dt} = (1 - \delta^k_{0,n})\pi_e \cdot r(t)P_L(n-1;t) - \pi_e \cdot r(t)P_L(n;t)$$

    This is solved using methods in the paper, and the generating function is:

    $$G_L(z;t) = \exp\left((z-1)\int_0^t \pi_e r(\tau)\, d\tau\right)$$

    So, L follows a Poisson distribution with the expected rate. A new section is added to the appendix which details the above (Section A.4.4)

29. **Eq. 18: What does the k superscript denote? What does "eff" denote?**

    k just denotes "Kronecker", we just want to differentiate the Kronecker delta from the treatment dosage δ. The superscript "eff" denotes the effective rate. For example, b^{eff} is the effective birth rate of a cell, considering the intrinsic birth rate b, the cost of resistance for a resistant cell c, and the effect from competition.

    We now explain this when we introduce these symbols.

30. **A.4, below Eq. 21: "holding for $k(z,t)$ large" - is that a reasonable assumption?**

    It is reasonable in the limit of small b_R^{eff} at fixed g_R^{eff} and whenever the binomial to Poisson distribution approximation becomes reasonable (for example at sufficiently small $p(t)$, which in any case occurs in most realistic switching sizes). We also see this approximation working well under the claimed assumptions from the new figure we share with a previous reviewer (Figure 1 in this document).

31. **A.4, end of paragraph below Eq. 21: It is unclear what are the "first" and "second" terms in Eq. 20, and what about in Eq. 21?**

    Thank you for pointing this out. We have changed the wording to make it clearer.

    In the Eq. (36), the terms referred to are the original exponential, and the second exponential, coming from the $\left(1 + \frac{1}{k(z,t)}\right)^{R_2(0)} \approx \exp\left(\frac{R_2(0)}{k(z,t)}\right)$ approximation.

32. **A.4, bottom of p.24: $G_{N_{R_2}}$ can be made arbitrarily close to 1 - what does this imply?**

    Note that $P_E^{SVG,R_2}(t) = G_{N_{R_2}}(1-\pi_e; t)$. The original statement was that it can be made arbitrarily close to 1 by increasing b_R^{eff} and keeping g_R^{eff} constant. But since $\exp(-\pi_e R_2(t))$ depends on the demographic event rates solely through $g_R^{\text{eff}}(t)$, our formula for the probability of extinction of SGV R_2 cells remains constant whilst the true probability of extinction is larger.

    We clarify what $G_{N_{R_2}}(1-\pi_e; t)$ represents in the main text.

33. **A.4, final paragraph (top of p.25): Was the discrepancy at intermediate treatment doses also expected?**

    Yes, birth rates and death rates are somewhat comparable. With $b = 1, d = 0.1$ and $\delta_1 = 1.2$, the ratio is $\dfrac{b-c}{d+\delta_1} \approx 0.77$.

34. **A.5: Define the abbreviations BGW and SDE**

    Thank you, we have now defined these abbreviations.

35. **A.5.1: It would be helpful to explain at the outset why you include two different derivations of the establishment probability. I did not understand why you invoked the Gillespie algorithm here instead of just referring to a birth-death process. The derivations leading up to Eq. 30 seemed unnecessary to include, since they are not novel, and Eq. 30 itself is a very well-known result. In the final paragraph, it is unclear what is the point of taking the limits and checking discrepancies. Please also clarify what is z here?**

    We initially presented two separate derivations for the establishment probability to compare the establishment probability we use to a more standard expression. As you mentioned, Eq. 30 is a well-known result for the birth-death process, while the expression derived in Appendix A.5 is a more general expression for a continuous-time, real valued branching process model. Since the Gillespie simulation is a numerical solution to the master stochastic equation corresponding to a linear birth-death process, the alternate expression for establishment probability in Eq. 30 is consistent with our simulations. However, we compared the two expressions for establishment probability and found negligible difference in the extinction probabilities.

    We now include the former Eq. 30 in Appendix A.5. The paragraph about checking discrepancies between the two expressions justifies why it is reasonable to use either expression for the computation of extinction probabilities. We also clarified the notation.

36. **A.6: Why is the simulation algorithm only considered "Gillespie-like" or "a variant of the Gillespie algorithm"? The description sounds like the exact Gillespie method. If there is some subtle difference, please highlight it and explain why you used a non-standard method.**

Thank you for pointing this out. Our simulation algorithm is indeed the same as the Gillespie algorithm. We now refer to it as the "Gillespie implementation" or "Gillespie simulations".

37. **Table A.1: Missing 0 < N(t) in the "Persistence" cases**

    We added the condition $0 < N(t)$ in Table S2.

38. **A.6.2: Why are there two different notations, gM(t) and gS/R(t)?**

    Thank you, we changed the notation to just g_M and clarified the use of other notation (g_S and g_R).

39. **A.6.2, below Eq. 35: what are "effective" birth and death rates?**

    The birth and death rates are implemented differently in the analytical approach and stochastic simulations. The analytical model is parameterised in terms of growth rates of cell lineages (g_S and g_R), whereas the stochastic simulation model requires separate birth and death rates. The "effective" birth and death rates used for the simulations take into account the effects of competition and treatment and are distinct from the intrinsic birth and death rates used in both models. Density dependence is implemented in the effective birth rates. We explain the motivation for introducing effective rates in Appendix A.7.2.

40. **A.6.2, 2nd-last paragraph: The choice of how to implement density dependence in birth vs. death rates is important, and should be stated in the main text as it could have an impact on the stochastic simulation results.**

    Thank you, we now added a sentence in the Methods section (Section 2.2) to state the choice of implementing density dependence in the birth rates.

41. **A.6.3: I'm confused by the approach of running multiple simulations with a single random seed. At which stage are you simulating in which environment(s), treatment 1 or 2? Why do you want to "eliminate differences due to stochasticity" (algorithm step 2) in a stochastic simulation?**

    For each run of the simulation, we use the same random seed so that the population trajectory before switching to the second treatment is the same for all switching sizes. For a given random seed, we switch from treatment 1 to treatment 2 at different switching sizes. Stochasticity before the second strike is eliminated so that all differences in the extinction probabilities are solely due to changes in switching sizes. Effectively, we run 100 independent simulation runs for each switching size.

    We have edited Section A.7 to include a better explanation of the simulation algorithm.

42. **A.6.3, algorithm step 6: Extinction probabilities are calculated using which equation? Wouldn't it be a better check on the analytical approximations also to simulate the occurrence of mutations and mutant lineage dynamics?**

    Extinction probabilities from the simulations are computed by dividing the number of simulation runs with an extinction event by the total number of simulation

runs performed. For each switching size and parameter combination, 100 independent replicates of the Gillespie algorithm are run. The extinction probability is only "calculated" in that we estimate it via $\frac{\text{\# of runs where } \exists t, N(t)=0}{\text{total number of runs}}$.

The primary quantity of interest is extinction probabilities, however, it is also useful to verify that the mean of the rescue population dynamics follow our analytical approximations, so we add a figure to this effect (Figure S9), along with a new appendix section (Section A.4.1) to relate the ODE-based and evolutionary rescue model with the linear birth-death-mutation model in the stochastic simulations.

We have also rephrased parts of Section A.7 to explain the computation of extinction probabilities better.

43. **A.7: This section is very brief and could easily (and usefully) have been included in the main text.**

    Thank you for the suggestion. We have now moved this section to the Methods section in the main text (Section 2.4).

44. **A.8: I'm struggling to follow this section. The drop in extinction probability is continual, not at a specific N: Do you mean an abrupt drop, maybe the steepest point of the curve? I don't understand what you mean by "favourable for ET" (please also define the abbreviation) or "beneficial in some cases". The end of the first paragraph is too vague: How is "ease of implementation" defined? Are "higher extinction probabilities" required for all N? Higher compared to what? Finally, what is the conclusion of this section? Which of the three metrics did you choose to use in the main text and why?**

    We remove this section from the appendix and add the following explanation in the Methods section (Section 2.3) describing the rule we use to compare N_q curves:

    When comparing different parameter values, a higher N_q curve indicates better treatment outcome. This is because it is favourable to reach high extinction probabilities at high switching sizes. For instance, a higher value of $N_{0.9}$ implies a wider window of opportunity due to a larger range of switching sizes for implementing a successful second strike. Furthermore, if a given N_q curve lies above the other, the same switching size can result in a higher extinction probability (see Figure S1). Therefore, the set of parameters for which the N_q curve lies above the others is expected to result in a better treatment outcome in terms of ease of implementation, higher extinction probabilities, or both.

References

[1] Kirill S. Korolev, Joao B. Xavier, and Jeff Gore. "Turning ecology and evolution against cancer". In: *Nature Reviews Cancer* 14.5 (May 2014), pp. 371–380. ISSN: 1474-1768. DOI: 10.1038/nrc3712. URL: https://doi.org/10.1038/nrc3712.

[2] Lotte Sewalt et al. "Influences of Allee effects in the spreading of malignant tumours". In: *Journal of Theoretical Biology* 394 (2016), pp. 77–92. ISSN: 0022-5193. DOI: https://doi.org/10.1016/j.jtbi.2015.12.024. URL: https://www.sciencedirect.com/science/article/pii/S002251931600031X.

[3] Philip Gerlee et al. "Autocrine signaling can explain the emergence of Allee effects in cancer cell populations". In: *PLOS Computational Biology* 18.3 (Mar. 2022), pp. 1–15. DOI: 10.1371/journal.pcbi.1009844. URL: https://doi.org/10.1371/journal.pcbi.1009844.

[4] Youness Azimzade, Abbas Ali Saberi, and Robert A. Gatenby. "Superlinear growth reveals the Allee effect in tumors". In: *Phys. Rev. E* 103 (4 Apr. 2021), p. 042405. DOI: 10.1103/PhysRevE.103.042405. URL: https://link.aps.org/doi/10.1103/PhysRevE.103.042405.

[5] *NCT04388839: Evolutionary Inspired Therapy for Newly Diagnosed, Metastatic, Fusion Positive Rhabdomyosarcoma*. May 2020. URL: https://clinicaltrials.gov/study/NCT04388839 (visited on 09/27/2020).

[6] *NCT05189457: A Phase IIA Study of Sequential ("First Strike, Second Strike") Therapies, Modeled on Evolutionary Dynamics of Anthropocene Extinctions, for High Risk Metastatic Castration Sensitive Prostate Cancer*. Dec. 2021. URL: https://clinicaltrials.gov/study/NCT05189457 (visited on 01/25/2022).

[7] *NCT06409390: A Pilot Study of Sequential ("First Strike, Second Strike") Therapies, Modeled on Evolutionary Dynamics of Anthropocene Extinctions, for Hormone Positive Metastatic Breast Cancer*. May 2024. URL: https://clinicaltrials.gov/study/NCT06409390 (visited on 04/26/2024).

[8] Troy Day and Andrew F. Read. "Does High-Dose Antimicrobial Chemotherapy Prevent the Evolution of Resistance?" In: *PLOS Computational Biology* 12.1 (Jan. 2016), pp. 1–20. DOI: 10.1371/journal.pcbi.1004689. URL: https://doi.org/10.1371/journal.pcbi.1004689.

[9] Hildegard Uecker, Sarah P. Otto, and Joachim Hermisson. "Evolutionary Rescue in Structured Populations". en. In: *The American Naturalist* 183.1 (Jan. 2014), E17–E35. ISSN: 0003-0147, 1537-5323.

[10] Yannick Viossat and Robert Noble. "A theoretical analysis of tumour containment". In: *Nature Ecology and Evolution* 5.6 (June 2021), pp. 826–835. ISSN: 2397334X.

[11] Robert A. Gatenby, Jingsong Zhang, and Joel S. Brown. "First strike-second strike strategies in metastatic cancer: Lessons from the evolutionary dynamics of extinction". In: *Cancer Research* 79.13 (2019), pp. 3174–3177. ISSN: 15387445.

[12] Robert A. Gatenby and Joel S. Brown. "Integrating evolutionary dynamics into cancer therapy". en. In: *Nature Reviews Clinical Oncology* 17.11 (Nov. 2020). Number: 11 Publisher: Nature Publishing Group, pp. 675–686. ISSN: 1759-4782. DOI: 10.1038/s41571-020-0411-1. URL: https://www.nature.com/articles/s41571-020-0411-1 (visited on 03/30/2023).

[13] Ivana Bozic et al. “Evolutionary dynamics of cancer in response to targeted combination therapy”. In: *eLife* 2 (June 2013). Ed. by Carl T Bergstrom, e00747. ISSN: 2050-084X. DOI: 10.7554/eLife.00747. URL: https://doi.org/10.7554/eLife.00747.

[14] Amjad Dabi et al. “Evolutionary rescue model informs strategies for driving cancer cell populations to extinction”. In: *bioRxiv* (2025). DOI: 10.1101/2024.11.26.625315. eprint: https://www.biorxiv.org/content/early/2025/01/13/2024.11.26.625315.full.pdf. URL: https://www.biorxiv.org/content/early/2025/01/13/2024.11.26.625315.

[15] Elysia C. Saputra et al. “Combination Therapy and the Evolution of Resistance: The Theoretical Merits of Synergism and Antagonism in Cancer”. In: *Cancer Research* 78.9 (Apr. 2018), pp. 2419–2431. ISSN: 0008-5472. DOI: 10.1158/0008-5472.CAN-17-1201. eprint: https://aacrjournals.org/cancerres/article-pdf/78/9/2419/2775336/2419.pdf. URL: https://doi.org/10.1158/0008-5472.CAN-17-1201.

[16] Christin Nyhoegen, Sebastian Bonhoeffer, and Hildegard Uecker. “The many dimensions of combination therapy: How to combine antibiotics to limit resistance evolution”. In: *Evolutionary Applications* 17.8 (2024). e13764 EVA-2023-262-OA.R1, e13764. DOI: https://doi.org/10.1111/eva.13764. eprint: https://onlinelibrary.wiley.com/doi/pdf/10.1111/eva.13764. URL: https://onlinelibrary.wiley.com/doi/abs/10.1111/eva.13764.

[17] Robert A. Gatenby et al. “Eradicating metastatic cancer and the eco-evolutionary dynamics of Anthropocene extinctions”. In: *Cancer Research* 80.3 (Feb. 2020), pp. 613–623. ISSN: 15387445.

August 17, 2025

GENETICS-2025-308287
Preventing evolutionary rescue in cancer using two-strike therapy

Dear Dr. Noble:

Three experts in the field have reviewed your manuscript, and I have read it as well. I am pleased to inform you that, with minor revisions, it is potentially suitable for publication in GENETICS. The reviewers have comments and concerns that need to be addressed in a revised manuscript. You can read their reviews at the end of this email.

As you will see, the reviewers still list many suggestions for improvement, although none stands as really major (perhaps with the exception of Reviewer 1 remarks about stating the novelty more clearly in comparison to previous work, and Reviewer 3 question about the rescue probability from R1,2 mutants). Addressing them will certainly further improve this nice study.

We look forward to receiving your revised manuscript. Please let the editorial office know approximately how long you expect to need for revisions.

Upon resubmission, please include:
1. A clean version of your manuscript;
2. A marked version of your manuscript in which you highlight significant revisions carried out in response to the major points raised by the editor/reviewers (track changes is acceptable if preferred);
3. A detailed response to the editor's/reviewers' comments and to the concerns listed above. Please reference line numbers in this response to aid the editors.

Additionally, please ensure that your resubmission is formatted for GENETICS.
https://academic.oup.com/genetics/pages/general-instructions

Follow this link to submit the revised manuscript: Link Not Available

Sincerely,

Thomas Lenormand
Associate Editor
GENETICS

Approved by:
Nicholas Barton
Senior Editor
GENETICS

Reviewer #1 :

Thanks to the authors for their extensive response and apologies for my slow reply, I hope I haven't slowed things down too much.

I'm generally quite happy with the current manuscript. My remaining minor comments are (line numbers in tracked changes pdf):

- lines 32-35: I think it is specifically the *term* "evolutionary rescue" that arose in a conservation context (first introduced by Gomulkiewicz & Holt 1995), and the same theory *has long been used* when extinction is the goal (eg, Coldman and Goldie's cancer models from the 70s and 80s)

- line 40: I'm not sure what "phenotypic heterogeneity" is and this term is not mentioned in the citation given (Alexander et al 2014). Do you mean "genetic variation"? More generally, this second paragraph could be more tailored to the approach you take, where two-strike therapy works at small populations sizes not due to genetic drift (loss of genetic variation) and Allee effects, but rather because the number of potential rescue mutants is minimized. My thought is just to better prepare readers for the model you next present.

- lines 65-66: I'm not sure you answer this first question or how answerable it is. Perhaps better to rephrase, e.g., how do we maximize the probability that the second strikle is successful?

- lines 73-74: It's unclear to me why relapse with a single strike is guaranteed in your model. I think there must always be some non-zero probability of extinction with a single strike in your simulations and you could also estimate the probability of extinction for a single strike with an analagous approach to the one you take for two strikes, i.e., \exp[-\pi_e R_1(0) - \pi_e \int_{0}^\infty \mu_1 S(t) dt], which is vanilla rescue theory (Orr a& Unckless 2008). It might be helpful to contrast your two-strike results with this to show how much the second strike helps?

- line 149: You mention considering R_2(0) at mutation selection balance in a few places and I at first worried this was assuming a constant population size as in classic population genetics. Of course the population size bit doesn't actually matter for the allele frequencies, as you state in the appendix, but it might be worth adding "in an exponentially growing tumour" to "mutation-selection balance" in the main text for clarity? For exponentially growing populations I tend to think about Luria-Delbruck-like calculations where mutations only happen at birth and there is no cost, in which case we'd have E[R_2(0)]=\mu_2 S(0) b t. However in your case of birth-independent mutations you have (footnote 6, page 24) E[R_2(0)] = \mu_2 S(0) t. I'm not sure this is worth mentioning but helps connect your model to others, like Coldman and Goldie.

- lines 219-251: The beginning of the results section still seems overly repetitive with the main text methods to me.

- lines 479-485: I like this summary of how your work updates rescue theory. However, I remain confused about the novelty of your study as explained. First, you say that you differ from previous theory by considering multiple (2) abrupt environmental changes, but the studies of cycling/fluctuating environments that you cite also have multiple abrupt environmental changes, and the cited model of Greenspoon and Mideo even has two types of stressful environments, just as you do. Second, you say that you model multiple (3) types of resistance to multiple (2) stressful environments, but the later cited Coldman and Goldie 1983 model allows for all types of resistance to n stressful environments that are applied sequentially (their n=2 case has 3 types of resistance, identical to your model). To be clear, I do think there is lots of novelty here, I just don't think it is precisely articulated yet.

Some very minor typos etc:

- lines 130-132: Super nitpicky, but the number of rescue mutants is -1 times the exponent.

- line 137 seems repetitive with line 128, can you merge the two sentences there?

- line 160: there is an extra "initial"

- line 182: increases steeply "with N"

- line 207: "the" literature

- line 264: "It" follows

- line 268: "the" rescue probability

- line 314: helpful to clarify you are talking about the efficacy of the first strike only?

- lines 343-344: Perhaps rephrase as stochastic extinction of a finite population always has a non-zero probability

- lines 558-562: helpful to add that assuming mutations occuring only at birth might change your turnover result, since larger b+d for a given b-d would not only reduce establishment but now also increase mutation?

Reviewer #2 :

The authors present an evolutionary rescue framework to model cancer cell survival under two consecutive chemotherapy ("strikes"). They navigate the parameter space with both analytical calculations and stochastic simulations to explore how the cancer cell pool responds to varying timing and efficacy of two "strikes". They show that the cell extinction probability (P_E) is usually optimized when the second "strike" is administered closely following the point where the first chemo achieved maximal effects ("nadir"), consistent with the clinical intuition. They also show that, under the two-strike strategy, the efficacy of the first "strike" doesn't need to be high to obtain robust treatment success (as measured by the range of pop sizes having high P_E). This solid work is a notable addition to the existing body of work on evolutionary rescue modelling of cancer, lending insights to both the modeling community and clinical practices.

In their revision, the authors comprehensively addressed mine and the other reviewers' comments and suggestions. They also

substantially improved the flow and clarity of their writing, as well as the readability of their figures. These changes made an already refreshing work even more enjoyable to read; general readers should now be able to appreciate the empirical implications of their models and results much better. I congratulate the authors on their good work and look forward to reading this manuscript in publication.

Reviewer #3 :

Please see attached document for comments. Regarding data availability, the issue is very minor: simply need to clarify where the code repository is available, with a URL (this is missing).

Comments on GENETICS-2025-308287 (Reviewer 3)

The authors have done a thorough and commendable job in addressing the many comments from the previous round of review. I believe these changes and the addition of several subsections and figures have made the manuscript substantially clearer. In particular, the restructuring of the Results with re-designed figures, combined with more precise definitions and more explanation of results, provides a much clearer narrative. I am satisfied with how the authors have addressed my previous major comments and almost all the minor comments.

I see only one possibly important issue remaining, regarding rescue probability from $R_{1,2}$ mutants. This relates to my previous major comment #4a and minor comment #28; and is mentioned in the revised manuscript on L139-142, addressed in detail in Appendix A.3, and also touched upon at the start of A.4.4.

- I'm afraid I'm still not on board with the calculation for rescue from $R_{1,2}$ mutants in the SGV. The equation in Appendix A3 (L985) takes account of the number of mutation events from either R_1 or R_2 to $R_{1,2}$ between time 0 and τ (i.e. in environment E1, i.e. pre-existing at the time of the second strike). However, each of these mutation events will give rise to a lineage generating possibly several $R_{1,2}$ cells within E1 by time τ. Each of these cells existing at time τ will then independently establish in E2 with probability π_e. This (π_e) is the establishment probability of an individual cell in environment E2, so it cannot be considered the probability of an entire lineage arising previously in E1 to have one or more established descendants in E2. Therefore it seems to me that the total number of $R_{1,2}$ mutants existing at time τ (accounting for replication within the lineages in E1), not only the number of mutations, needs to be accounted for. Likewise, in the description at the start of section A.4.4, it is claimed that lineages neither 'divide' nor 'die', but (subject to clarifying what it means for a lineage, as opposed to a cell, to divide/die), I don't think this is true in E1.

Otherwise, my remaining comments (though numerous, due to the substantive revisions and new material) are extremely minor.

- Intro: Confusing wording around what constitutes sequential or combination therapy. L49: "In conventional combination or sequential therapy, the second or subsequent treatments are usually given..." – Doesn't this only refer to sequential, not combination, therapy? L51: "Another conventional strategy is to simultaneously administer multiple drugs..." – isn't this simply combination therapy?
- Fig. 1, Part 2 is a nice addition, but shouldn't the rescued population in E2 be labelled $R_{1,2}$ (not R_1)?
- L248: I don't see how this is illustrated in Fig. 2, 2nd column – possibly the wrong figure reference?
- L340-341: It would be helpful to clarify whether you mean intrinsic growth rate (b-d) and turnover (b+d), as opposed to growth rate and turnover in presence of the drug ($b-d-\delta$ and $b+d+\delta$, respectively). Similar point on L446 of the Discussion.

- Fig. 4 caption: Please indicate which part is describing panel (C). Also, the last couple of sentences (“Each subplot shows…”) appears misplaced.
- L372-377: Please clarify which of these observations relate to before-nadir switching, after-nadir switching, or both. (For example, the second observation only holds for before-nadir as shown in Fig. 5C, 1st panel.)
- Fig. 5C: the caption refers to a solid black line, but I don’t see this on the plot.
- L382: Should this refer to “a smaller *resistant* population” or “*potential rescue*” rather than “rescue”? Same question on L454 (“the number of rescue mutants”).
- L416: If possible to state briefly, it would be helpful to explain why your results differ from the previous results by Gatenby et al.
- Data & code availability: Please provide a URL for the code repository (I assume this is on GitHub but it isn’t actually stated in the reference list).
- Appendix, L836: Typo? (Shouldn’t decreasing resistant populations lead to a *lower* probability of rescue?)
- Footnote 10: I am convinced by the result, but didn’t follow a couple of the details.
    - The approximation $\dot{R}_1 = \gamma_1 R_1$ close to t_{opt}: shouldn’t this only hold before switching (in E1), not after?
    - In the equation for RM, where does this equality come from? Shouldn’t there be a μ involved somewhere? Sorry if I’ve forgotten something; please refer back to an earlier equation if relevant.
- Around L881-2: “For a given resistant population $R_1(\tau)$, R_1 cells will generate more $R_{1,2}$ mutants in phase 1 when incurring a large cost of resistance.” I still don’t fully understand the intuition; consider (at your discretion) adding slightly more explanation. Specifically, I understand that the R1 population is increasing over treatment phase 1 (provided $c < b-d$), so it will take longer to reach a given population size if the cost is higher. However, it’s not obvious to me that the integral of R1 over this time (which should be proportional to the number of mutations generated under your model) will also be larger if cost is higher.
- L939 / Response to my previous comment #20 re: Haldane’s 2s approximation. This is fine, but then I’m not sure why you mention the approximation at all: It is not likely to hold for treatment-relevant parameters and it does not appear ever to be used in your results. Consider (at your discretion) removing.
- New appendix A.4.1.1: This is a good addition, but the set-up/definitions are a bit vague. It would be helpful to make the following clarifications:
    - State that you are setting up a master equation, define *P* explicitly (probability of…), and define $\{x_i\}$ explicitly (i.e. population sizes, not just “referring to” populations)
    - Write the expressions for each type of ‘event’ as equations (i.e. the probability of a given transition in state space = …)

  - L1023-4: Note that the *per capita* event rates – but not the total event rates as written – are independent of the state variables.
  - The two equations for mutation events appear repetitive: all combinations from *i* to *j* are covered by either equation.
  - L1031-2: "Eq. 31 can be converted into a PDE…" – this is too vague. This is explained better in a later section (L1076-7) but should rather be explained at first usage.
- Relating to my previous comment #29: The Kronecker delta still has not been defined the first time it appears (now in A.4.1.1). Also, while the effective birth and death rates are now loosely explained in A.4.1.1, ideally the reader would not have to look ahead to find equations, and Eq. 51-52 would have to be modified anyway by taking K $\rightarrow$ infinity for the case without competition.
- L1050 mentions "just one round of therapy", but refers to Fig. S9 (left column), which seems to show two strikes.
- A.4.2, L1056: It would be helpful to remind the reader here what RM means (number of rescue mutants in the ODE solution?)
- L1056: Typo? "Consider the cells rescue mutants…"
- L1068-9, with reference to Fig. S10: I'm not quite clear on the point you're making here, since Fig. S10 does not directly illustrate extinction probabilities, but rather rescue mutants. Is the point to show that $E[N_{RM}] \neq RM$ for these parameters, and this is why the analytical approximation for extinction probability is poor?
- L1100: "turnover is roughly proportional to the variance of the R2 population size" – this isn't obvious; it would help to refer to section A.5 where variance of a birth-death process is given.
- Figure S3 caption: Please add whether the population sizes indicated by the coloured bar are from the ODE solutions.
- Fig. S7: Please expand the caption, as currently only some of the panels are described. Please mention if all other parameters are fixed to their default values. Also, in panels (A) and (B) legends, confusingly the values of μ are not arranged in strictly increasing or decreasing order; ideally adjust this.
- Fig. S9: Please add to the caption what the black lines in the top panels represent (individual stochastic trajectories?). For the bottom panels, it would be helpful to compare the magnitude directly to the other term(s) of the ODE involving only the first moments, but I leave this to your discretion.
- Fig. S10 caption: "the calculated number of rescue mutants" – calculated from the analytical model?
- It would be useful to refer to Fig. S11 within the text of Appendix A3/A4 (currently it is only mentioned in the main text).

Reviewer 1

Comments

Thanks to the authors for their extensive response and apologies for my slow reply, I hope I haven't slowed things down too much. I'm generally quite happy with the current manuscript. My remaining minor comments are (line numbers in tracked changes pdf):

- **lines 32-35: I think it is specifically the *term* "evolutionary rescue" that arose in a conservation context (first introduced by Gomulkiewicz & Holt 1995), and the same theory *has long been used* when extinction is the goal (eg, Coldman and Goldie's cancer models from the 70s and 80s)**

  We have rephrased the sentence to clarify that the term, not the theory, arose in a conservation context. The sentence now cites Gomulkiewicz & Holt (1995), Coldman & Goldie (1983) and another paper that applies the theory to controlling bacteria.

- **line 40: I'm not sure what "phenotypic heterogeneity" is and this term is not mentioned in the citation given (Alexander et al 2014). Do you mean "genetic variation"? More generally, this second paragraph could be more tailored to the approach you take, where two-strike therapy works at small populations sizes not due to genetic drift (loss of genetic variation) and Allee effects, but rather because the number of potential rescue mutants is minimized. My thought is just to better prepare readers for the model you next present.**

  Replaced the term "phenotypic heterogeneity" with "genetic variation" and edited the paragraph to highlight that the advantage of a small population is that the probability of rescue decreases.

- **lines 65-66: I'm not sure you answer this first question or how answerable it is. Perhaps better to rephrase, e.g., how do we maximize the probability that the second strike is successful?**

  We have rephrased.

- **lines 73-74: It's unclear to me why relapse with a single strike is guaranteed in your model. I think there must always be some non-zero probability of extinction with a single strike in your simulations and you could also estimate the probability of extinction for a single strike with an analogous approach to the one you take for two strikes, i.e., $\exp[-\pi_e R_1(0) - \pi_e \int_0^\infty \mu_1 S(t)dt]$, which is vanilla rescue theory (Orr & Unckless 2008). It might be helpful to contrast your two-strike results with this to show how much the second strike helps?**

  We have rephrased the line to say that tumour relapse occurs with a high probability with single strike and conventional treatments. We quantify the superiority

of two-strike versus one-strike therapy in Appendix A.1.3, which we now reference in the Methods: “Equation 2 also assumes that the probability of extinction during E_1 in the expression for total extinction probability is negligible. We verify this assumption in Appendix A.1.3, where we compare extinction probabilities from single-strike and two-strike therapies. This holds for all our parameter values (see Appendix A.4.1 and A.4.4).”

- **line 149: You mention considering $R_2(0)$ at mutation selection balance in a few places and I at first worried this was assuming a constant population size as in classic population genetics. Of course the population size bit doesn’t actually matter for the allele frequencies, as you state in the appendix, but it might be worth adding “in an exponentially growing tumour” to “mutation-selection balance” in the main text for clarity? For exponentially growing populations I tend to think about Luria-Delbruck-like calculations where mutations only happen at birth and there is no cost, in which case we’d have $E[R_2(0)] = \mu_2 S(0) bt$. However in your case of birth-independent mutations you have (footnote 6, page 24) $E[R_2(0)] = \mu_2 S(0) t$. I’m not sure this is worth mentioning but helps connect your model to others, like Coldman and Goldie.**

  We have added “in an exponentially growing population” to the referred line and in the Appendix. We have also added the following sentence to footnote 6: “If the number of mutations from S to R_i is not only proportional to S but also to the birth rate of S cells, that is, $\dot{R}_i = \gamma_i R_i + \mu_i b_S S$, then μ_i should be replaced by $\mu_i b_S$ in the formula and we would get $R_i = \frac{\mu_i b_S}{\gamma_S} S \ln S$, or equivalently $R_i = \mu_i b_S S t$.”

- **lines 219-251: The beginning of the results section still seems overly repetitive with the main text methods to me.**

  We have removed the repetitive parts.

- **lines 479-485: I like this summary of how your work updates rescue theory. However, I remain confused about the novelty of your study as explained. First, you say that you differ from previous theory by considering multiple (2) abrupt environmental changes, but the studies of cycling/fluctuating environments that you cite also have multiple abrupt environmental changes, and the cited model of Greenspoon and Mideo even has two types of stressful environments, just as you do. Second, you say that you model multiple (3) types of resistance to multiple (2) stressful environments, but the later cited Coldman and Goldie 1983 model allows for all types of resistance to n stressful environments that are applied sequentially (their $n = 2$ case has 3 types of resistance, identical to your model). To be clear, I do think there is lots of novelty here, I just don’t think it is precisely articulated yet.**

  We have revised the first relevant sentence to better characterize the differences between our work and prior studies: “While previous studies have examined evolutionary rescue when the environment fluctuates between harsh and favourable states (Greenspoon and Mideo 2017; Marrec and Bank 2023; Marrec and Bitbol

2020), we consider a sequence of distinct harsh states, such that the timing of environmental change is a controllable parameter." We have also revised the second relevant sentence to acknowledge what is similar between our approach and that of Coldman and Goldie: "We have instead developed the case with three types of resistance to two types of environmental change, in the spirit of the classic chemotherapy model of Coldman and Goldie (1983)."

Minor Typos

- **lines 130-132: Super nitpicky, but the number of rescue mutants is -1 times the exponent.**

  We have added "the absolute value of. . . " before the statement.

- **line 137 seems repetitive with line 128, can you merge the two sentences there?**

  Done. Merged sentence: "A key assumption is that the number of rescue mutants is Poisson distributed, which introduces the negative exponential in the expression."

- **line 160: there is an extra "initial"**

  Replaced the first "initial" with "the".

- **line 182: increases steeply "with N"**

  Done

- **line 207: "the" literature**

  Done

- **line 264: "It" follows**

  Done

- **line 268: "the" rescue probability**

  Done

- **line 314: helpful to clarify you are talking about the efficacy of the first strike only?**

  Indeed, in this section we make the claim for treatment 1. The title was now changed to "Higher efficacy of the first treatment is not necessarily beneficial for treatment".

- **lines 343-344: Perhaps rephrase as stochastic extinction of a finite population always has a non-zero probability**

  Rephrased: "Note that if the first treatment's efficacy is too low then the population size will never become small enough to have a non-negligible probability of stochastic extinction."

- **lines 558-562: helpful to add that assuming mutations occurring only at birth might change your turnover result, since larger $b + d$ for a given $b - d$ would not only reduce establishment but now also increase mutation?**

  We have dded a sentence to the end of the paragraph: "Although we have used simple models with minimal assumptions to ensure that our main findings are qualitatively robust, we have not explored all plausible functional forms. For example, in a model in which mutations occur only at the time of cell division, the number of potential rescue mutants (and therefore extinction probability) would depend on the number of divisions, while in our model it depends on the population size. The dependence of mutations on the birth rate would change some of our results, especially those given in 3.7."

Reviewer 2

The authors present an evolutionary rescue framework to model cancer cell survival under two consecutive chemotherapy ("strikes"). They navigate the parameter space with both analytical calculations and stochastic simulations to explore how the cancer cell pool responds to varying timing and efficacy of two "strikes". They show that the cell extinction probability (P_E) is usually optimized when the second "strike" is administered closely following the point where the first chemo achieved maximal effects ("nadir"), consistent with the clinical intuition. They also show that, under the two-strike strategy, the efficacy of the first "strike" doesn't need to be high to obtain robust treatment success (as measured by the range of pop sizes having high P_E). This solid work is a notable addition to the existing body of work on evolutionary rescue modelling of cancer, lending insights to both the modeling community and clinical practices.

In their revision, the authors comprehensively addressed mine and the other reviewers' comments and suggestions. They also substantially improved the flow and clarity of their writing, as well as the readability of their figures. These changes made an already refreshing work even more enjoyable to read; general readers should now be able to appreciate the empirical implications of their models and results much better. I congratulate the authors on their good work and look forward to reading this manuscript in publication.

Thank you for the summary and your comments.

Reviewer 3

The authors have done a thorough and commendable job in addressing the many comments from the previous round of review. I believe these changes and the addition of several subsections and figures have made the manuscript substantially clearer. In particular, the restructuring of the Results with re-designed figures, combined with more precise definitions and more explanation of results, provides a much clearer narrative. I am satisfied with how the authors have addressed my previous major comments and almost all the minor comments.

We appreciate your many detailed comments.

Major Comments

I see only one possibly important issue remaining, regarding rescue probability from $R_{1,2}$ mutants. This relates to my previous major comment #4a and minor comment #28; and is mentioned in the revised manuscript on L139-142, addressed in detail in Appendix A.3, and also touched upon at the start of A.4.4.

- **I'm afraid I'm still not on board with the calculation for rescue from $R_{1,2}$ mutants in the SGV. The equation in Appendix A3 (L985) takes account of the number of <u>mutation events</u> from either R_1 or R_2 to $R_{1,2}$ between time 0 and τ (i.e. in environment E_1, i.e. pre-existing at the time of the second strike). However, each of these mutation events will give rise to a lineage generating possibly several $R_{1,2}$ cells within E_1 by time τ. Each of these cells existing at time τ will then independently establish in E_2 with probability π_e. This (π_e) is the establishment probability of an individual cell in environment E_2, so it cannot be considered the probability of an entire lineage arising previously in E_1 to have one or more established descendants in E_2. Therefore it seems to me that the total number of $R_{1,2}$ <u>mutants</u> existing at time τ (accounting for replication within the lineages in E_1), not only the number of mutations, needs to be accounted for. Likewise, in the description at the start of section A.4.4, it is claimed that lineages neither 'divide' nor 'die', but (subject to clarifying what it means for a lineage, as opposed to a cell, to divide/die), I don't think this is true in E_1.**

  The reviewer is right but so are we! There is a conceptual difference between R_2 cells and $R_{1,2}$ cells. Indeed, R_2 cells have a positive growth rate only in the second environment (E_2). Therefore, in E_1, R_2 cells should not yet be considered as potential rescue mutants. It is only in E_2 that they get the ability to rescue the population. So indeed, as potential rescue mutants, we count all R_2 cells existing when switching to treatment 2 (time τ), and those that appear by mutation of an S cell during E_2.

  By contrast, $R_{1,2}$ cells are favoured by selection in both environments E_1 and E_2. Moreover, the dynamics of $R_{1,2}$ cells do not change between these two environments. That is, the parameters for the $R_{1,2}$ cells (birth, death) are the same in both environment, so the probability of establishment is the same whether a $R_{1,2}$ cell occurs in E_1 or E_2. It follows that there are two ways to compute the probability that $R_{1,2}$ cells will rescue the population. The first one is to do as the reviewers suggest, that is, to proceed as we do for R_2 cells. The second one, which we chose, is to make no difference between E_1 and E_2 for $R_{1,2}$ cells: each time a $R_{1,2}$ cell appears by mutation, whether during E_1 or E_2, it has a probability π_e of establishing and founding a rescue lineage.

  In other words, for $R_{1,2}$ cells, it is as if there were just one strike, occurring at time 0. So we count the $R_{1,2}$ cells present at time 0 (but this quantity is negligible), and those that appear by mutations after time 0.

We have added the following sentences in the next to last paragraph of Section 2.1 (Analytical methods) : “Note that we do not compute the number of rescue mutants in the same way for $R_{1,2}$ cells and R_2 cells. As R_2 cells have a positive fitness only in environment E_2, we consider as potential founders of rescue lineages the R_2 cells that are present when switching to treatment 2 and those that arise later by mutation of S cells. By contrast, $R_{1,2}$ cells have positive fitness and the same establishment probability in both environments E_1 and E_2. Thus, the expected number of $R_{1,2}$ rescue mutants is simply this establishment probability multiplied by the expected number of $R_{1,2}$ lineages, which is the expected number of $R_{1,2}$ cells present at time 0 or arising by mutation, whether in E_1 or E_2.”

We have also added, in the second paragraph of Section 2.1, the parenthesis in the following sentence: “In our model [potential rescue] lineages comprise all $R_{1,2}$ cells (since they have a positive fitness in both E_1 and E_2), R_2 cells present at the switching time, and R_2 cells that arise after the switch.”

Lastly, we have removed the reference to division or death of a lineage.

Minor Comments

Otherwise, my remaining comments (though numerous, due to the substantive revisions and new material) are extremely minor.

- **Intro: Confusing wording around what constitutes sequential or combination therapy. L49: “In conventional combination or sequential therapy, the second or subsequent treatments are usually given…” – Doesn’t this only refer to sequential, not combination, therapy? L51: “Another conventional strategy is to simultaneously administer multiple drugs…” – isn’t this simply combination therapy?**

  We have edited the paragraph to clarify the differences between sequential, combination and multi-strike therapy:

  “The main differences between conventional cancer treatment strategies and multi-strike therapy are in the timing of the strikes and the use of evolutionary principles to guide clinical decision making. In combination therapy, multiple drugs with collateral sensitivities are administered simultaneously (Chakrabarti and Michor 2017). In conventional sequential therapy, a second treatment is typically started during relapse, when the first treatment has evidently failed. In multi-strike therapy (also known as extinction therapy (Gatenby, Artzy-Randrup, et al. 2020)), the idea is instead to switch treatments when the tumour is at its weakest, when it may well be clinically undetectable. The success rate of multi-strike therapy is expected to be highly sensitive to the timing and severity of the second and any subsequent strikes. Based on computational modelling, it has been suggested that the best time to switch to the second treatment occurs while the tumour is still shrinking in response to the first treatment (Gatenby, Artzy-Randrup, et al. 2020).”

- **Fig. 1, Part 2 is a nice addition, but shouldn't the rescued population in E_2 be labelled $R_{1,2}$ (not R_1)?**

  The solid line in Figure 1 shows rescue in the absence of a second treatment (so, with treatment 1 only). The dashed line shows a possible extinction if the second strike is applied. This is mentioned in the figure caption.

  We have now edited the figure to clearly show that the rescued population is without a second strike. While it would be composed mainly of R_1 cells and some $R_{1,2}$ cells, we do not show this in the figure because the aim is to illustrate before-nadir and after-nadir switching points.

- **L248: I don't see how this is illustrated in Fig. 2, 2nd column – possibly the wrong figure reference?**

  Replaced with the correct figure reference.

- **L340-341: It would be helpful to clarify whether you mean intrinsic growth rate ($b - d$) and turnover ($b + d$), as opposed to growth rate and turnover in presence of the drug ($b - d - \delta$ and $b + d + \delta$, respectively). Similar point on L446 of the Discussion.**

  Yes, in these cases we mean intrinsic growth rate and turnover. We have edited the text accordingly.

- **Fig. 4 caption: Please indicate which part is describing panel (C). Also, the last couple of sentences ("Each subplot shows…") appears misplaced.**

  Removed the last line of the caption and indicated which part describes panel (C).

- **L372-377: Please clarify which of these observations relate to before-nadir switching, after-nadir switching, or both. (For example, the second observation only holds for before-nadir as shown in Fig. 5C, 1st panel.)**

  In this paragraph, we make statements that hold for both before-nadir and after-nadir switching points, or those that are just about the optimal $N(\tau)$ or the optimal extinction probability. For instance, in the second observation, we say that the optimal extinction probability decreases as the birth rate increases by observing the optimal extinction probability contours shown in white. This observation is followed by an explanation of why the population nadir would be larger for a higher birth rate, which is true regardless of the switching size.

- **Fig. 5C: the caption refers to a solid black line, but I don't see this on the plot.**

  Removed mention of the solid black line.

- **L382: Should this refer to "a smaller resistant population" or "potential rescue" rather than "rescue"? Same question on L454 ("the number of rescue mutants").**

  Done

- L416: If possible to state briefly, it would be helpful to explain why your results differ from the previous results by Gatenby et al.

  We have added a subsection to the Discussion detailing how important differences between our model and that of Gatenby et al. account for our contrasting conclusions.

- **Data & code availability: Please provide a URL for the code repository (I assume this is on GitHub but it isn't actually stated in the reference list).**

  We now provide the URL in the "Data & code availability" section.

- **Appendix, L836: Typo? (Shouldn't decreasing resistant populations lead to a *lower* probability of rescue?)**

  Done

- **Footnote 10: I am convinced by the result, but didn't follow a couple of the details.**

  We have corrected some typos and omissions.

  – **The approximation $\dot{R}_1 = \gamma_1 R_1$ close to t_{opt}: shouldn't this only hold before switching (in E_1), not after?**

    Indeed, the approximation $\dot{R}_1 = \gamma_1 R_1$ only holds before switching. We now write: "With the approximations $\dot{R}_1 = \gamma_1 R_1$ close to t_{opt} (before switching)". We also clarified that by $S(t_{opt})$, $R_1(t_{opt})$, we mean the size of these populations at time t_{opt} if we switch at or after t_{opt}.

  – **In the equation for RM, where does this equality come from? Shouldn't there be a μ involved somewhere? Sorry if I've forgotten something; please refer back to an earlier equation if relevant.**

    The end result is correct but we omitted additive and multiplicative constants and there were typos on the sign of some quantities. We now write: In the reference case (18), for any $\Delta > 0$, $RM(t_{\text{opt}} + \Delta) \leq RM(t_{\text{opt}} - \Delta)$. Indeed, let $S(t_{opt})$ and $R_1(t_{opt})$ denote the S and R_1 populations at the optimal switching time t_{opt} if switching at (or after) this time. With the approximations $\dot{R}_1 = \gamma_1 R_1$ close to t_{opt} (before switching), and letting $\alpha = \delta_1 - \gamma_S$, $\beta = \gamma_1$, we get that $\alpha\alpha_S S(t_{\text{opt}}) = \beta\alpha_1 R_1(t_{\text{opt}})$ and (recall (20)):

$$\frac{1}{\mu_2}\left[RM(t_{\text{opt}} + \Delta) - \pi_{12}R^*_{12}(0)\right] = S(t_{\text{opt}})e^{-\alpha\Delta} + R_1(t_{\text{opt}})e^{\beta\Delta}$$
$$= c\left[\frac{e^{-\alpha\Delta}}{\alpha} + \frac{e^{\beta\Delta}}{\beta}\right]$$

    where $c = \alpha\alpha_S S(t_{\text{opt}}) = \beta\alpha_1 R_1(t_{\text{opt}})$. It follows that for $\Delta > 0$,

$$RM(t_{\text{opt}} + \Delta) < RM(t_{\text{opt}} - \Delta) \Leftrightarrow \frac{e^{-\alpha\Delta}}{\alpha} + \frac{e^{\beta\Delta}}{\beta} < \frac{e^{\alpha\Delta}}{\alpha} + \frac{e^{-\beta\Delta}}{\beta}$$
$$\Leftrightarrow 2\frac{\sinh(\beta\Delta)}{\beta} < 2\frac{\sinh(\alpha\Delta)}{\alpha} \Leftrightarrow \frac{\sinh(\beta\Delta)}{\beta\Delta} < \frac{\sinh(\alpha\Delta)}{\alpha\Delta} \Leftrightarrow \beta < \alpha$$

where $\sinh(x) = \frac{e^x - e^{-x}}{2}$ is the hyperbolic sine function, and the last equivalence follows from the standard fact that $x \to \frac{\sinh(x)}{x}$ is increasing on $(0, +\infty)$. Since $\alpha = \delta_1 - \gamma_S > \gamma_1 = \beta$, the result follows.

- **Around L881-2: "For a given resistant population $R_1(\tau)$, R_1 cells will generate more $R_{1,2}$ mutants in phase 1 when incurring a large cost of resistance." I still don't fully understand the intuition; consider (at your discretion) adding slightly more explanation. Specifically, I understand that the R_1 population is increasing over treatment phase 1 (provided $c < b - d$), so it will take longer to reach a given population size if the cost is higher. However, it's not obvious to me that the integral of R_1 over this time (which should be proportional to the number of mutations generated under your model) will also be larger if cost is higher.**

  We have added in a footnote the following text: "Let T be the time at which the R_1 population reaches a given size. In our model, the expected number of $R_{1,2}$ cells generated by mutation of R_1 cells between 0 and T is $\int_0^T \mu_2 R_1(t)\, dt = \mu_2 T \bar{R}_1$ where $\bar{R}_1 = \frac{1}{T}\int_0^T R_1(t)\, dt$ is the average size of the R_1 population until time T. A larger cost of resistance leads to a slower growth of the R_1 population, hence a larger duration T to grow up to a given size (i.e. more time to generate mutations). This thus leads to a larger number of mutations from R_1 to $R_{1,2}$."

  In case the reviewer does not find this explanation convincing, here are other attempts at explaining what happens, which we did not include in order to remain concise.

  Here is an intuition. Consider a discrete model. Assume that the R_1 population takes q_n units of time to grow from size n to size $n + 1$. During this time, we assume that the expected number of mutations from R_1 to R_{12} it generates per time unit is proportional to the R_1 population, that is to n. So the expected total number of mutations it generates during the q_n units of time it takes to go from $R_1 = n$ to $R_1 = n + 1$ is proportional to $n \times q_n$. The larger q_n, the larger the expected number of mutations. More precisely, if the growth occurs twice slower, then the expected number of mutations doubles.

  Here is a more formal explanation. Consider first two functions $f_1(t) = t$ and $f_2(t) = t/2$. The first one reaches size 1 at time $t_1 = 1$. The second one takes twice as long to grow to a given size and so reaches size 1 at time $t_2 = 2$. The integral of f_2 between 0 and t_2 is equal to 1, which is twice the integral of f_1 between 0 and t_1. Geometrically, this is because the graph of f_2 has the same shape as the graph of f_1, except that time is dilated for f_2, so that for a given increment dy, it takes twice as much time to grow from y to $y + dy$ than for f_1. As a result, the area under the curve of f_2 between 0 and t_2 is twice the area under the curve of f_1 between 0 and t_1. More generally, letting $M \in \mathbb{R}$, for any function f_1, letting $f_2(t) = f_1(t/\lambda)$, the integral of f_2 between time 0 and the first time $t > 0$ such that $f_2(t) = M$ is λ times the integral of f_1 between time

0 and the first time such that $f_1(t) = M$. This may be shown using a change of variable in the integral.

In our case, during treatment 1, the number of R_1 cells grows roughly exponentially: $R_1(t) \approx \tilde{R}_1(0)e^{g_1 t}$ (this approximation is not good for small t but is quite good for large t, which is the part that contributes most for the integral). Fix a threshold $\bar{R}_1$, and let τ denote the time at which $R_1(t) = \bar{R}_1$, so that $\tilde{R}_1(0)e^{g_1\tau} = \bar{R}_1$. Then :

$$\int_0^\tau R_1(t)dt \approx \int_0^\tau \tilde{R}_1(0)e^{g_1 t}dt = \left[\frac{\tilde{R}_1(0)e^{g_1 t}}{g_1}\right]_0^\tau = \frac{1}{g_1}\left(\bar{R}_1 - \tilde{R}_1(0)\right)$$

so the integral is inversely proportional to g_1. If there is a large cost of resistance, then g_1 is smaller, $1/g_1$ larger, and so the integral is larger.

So indeed, a large cost of resistance, by slowing down the growth of R_1 cells, increases the integral of R_1 during the time it takes for R_1 to grow to a given size. Of course, the reason why we consider the integral R_1 is that in our model, the number of mutations from R_1 to R_{12} is proportional to R_1. If the number of mutations was proportional to the net growth-rate, i.e., to dR_1/dt, then the number of mutations during the time needed for R_1 to grow up uo a given size would be independent of the speed at which this growth occurs.

- **L939 / Response to my previous comment #20 re: Haldane's $2s$ approximation. This is fine, but then I'm not sure why you mention the approximation at all: It is not likely to hold for treatment-relevant parameters and it does not appear ever to be used in your results. Consider (at your discretion) removing.**

  We have removed the references to Handane's $2s$ approximation and have slightly rephrased the next sentence.

- **New appendix A.4.1.1: This is a good addition, but the set-up/definitions are a bit vague. It would be helpful to make the following clarifications:**

  - **State that you are setting up a master equation, define P explicitly (probability of...), and define $\{x_i\}$ explicitly (i.e. population sizes, not just "referring to" populations)**

    P is now defined explicitly (and now it is clearer what x_i are).
  - **Write the expressions for each type of 'event' as equations (i.e. the probability of a given transition in state space = ...)**

    Done
  - **L1023-4: Note that the *per capita* event rates – but not the total event rates as written – are independent of the state variables.**

    We now specify that per capita event rates are independent of the state variables.

  - **The two equations for mutation events appear repetitive: all combinations from i to j are covered by either equation.**

    The last equation with ν transitions are removed.

  - **L1031-2: "Eq. 31 can be converted into a PDE…" – this is too vague. This is explained better in a later section (L1076-7) but should rather be explained at first usage.**

    We provide intermediate steps for Eq. 32 of the main text to make the statement clearer.

- **Relating to my previous comment #29: The Kronecker delta still has not been defined the first time it appears (now in A.4.1.1). Also, while the effective birth and death rates are now loosely explained in A.4.1.1, ideally the reader would not have to look ahead to find equations, and Eq. 51-52 would have to be modified anyway by taking $K \to \infty$ for the case without competition.**

  Thank you. We define the Kronecker delta in its first use. Also, we remove reference to the effective birth and death rate notation in this section (since in the case without competition, the birth and death rates are simply b, d respectively).

- **L1050 mentions "just one round of therapy", but refers to Fig. S9 (left column), which seems to show two strikes.**

  This was a typo. We change this to "Significant deviations between the rescuing population and its ODE counterpart occur when there is a non-negligible chance of extinction, for example from applying two rounds of therapy"

- **A.4.2, L1056: It would be helpful to remind the reader here what RM means (number of rescue mutants in the ODE solution?)**

  We now refer back to the equation in the main text that first defines RM(τ).

- **L1056: Typo? "Consider the cells rescue mutants…"**

  This has been removed and we now directly mention "We consider the R_1 cell contribution towards $R_{1,2}$ rescue mutants."

- **L1068-9, with reference to Fig. S10: I'm not quite clear on the point you're making here, since Fig. S10 does not directly illustrate extinction probabilities, but rather rescue mutants. Is the point to show that $E[N_{\textbf{RM}}] \neq$ RM for these parameters, and this is why the analytical approximation for extinction probability is poor?**

  The point was to mention that despite the mean number of rescue mutants generated in the stochastic model matching with that calculated from the ODE model, there is still a discrepancy in the probability of extinction at low c. We break off this point into a separate paragraph:

  "Recall that there is a discrepancy between the analytical formula for the extinction probability and the simulation results at low costs of resistance (Figure 4). Even though RM(τ) is still the mean number of rescue mutants, at low costs

of resistance the distribution of the number of rescue mutants is not necessarily Poisson (which is shown in the below section)."

- **L1100: "turnover is roughly proportional to the variance of the R_2 population size" – this isn't obvious; it would help to refer to section A.5 where variance of a birth-death process is given.**

  Yes, we refer to section A.5, and slightly modify the statement to say that the variance of the R_2 population size increases with turnover.

- **Figure S3 caption: Please add whether the population sizes indicated by the coloured bar are from the ODE solutions.**

  We specify the population sizes in the colored bar is indeed from the ODE model.

- **Fig. S7: Please expand the caption, as currently only some of the panels are described. Please mention if all other parameters are fixed to their default values. Also, in panels (A) and (B) legends, confusingly the values of μ are not arranged in strictly increasing or decreasing order; ideally adjust this.**
  Done

- **Fig. S9: Please add to the caption what the black lines in the top panels represent (individual stochastic trajectories?). For the bottom panels, it would be helpful to compare the magnitude directly to the other term(s) of the ODE involving only the first moments, but I leave this to your discretion.**

  Thank you. We clarify in the caption that the black lines are individual stochastic traces. Additionally, we add a footnote to section A.4.1.2 to clarify what is meant for the normalized covariance term to be "negligeable".

  Added as footnote: "It suffices that CoV$[X, N_S + N_{R_1} + N_{R_2} + N_{R_{1,2}}]/(\mathbb{E}[X]K)$ for $X \in \{R_2, R_1, S\}$ be much smaller than unity. And indeed, all such terms remain below $\sim$0.05"

- **Fig. S10 caption: "the calculated number of rescue mutants" – calculated from the analytical model?**

  We now mention that it is calculated using the ODE model.

- **It would be useful to refer to Fig. S11 within the text of Appendix A3/A4 (currently it is only mentioned in the main text).**

  Added figure reference in Appendix A.3 and A.4.3.

October 23, 2025
RE: GENETICS-2025-308622

Dr. Robert Noble
City University of London Department of Mathematics
Department of Mathematics
City St George's, University of London
Northampton Square
London, N/A EC1V 0HB
United Kingdom

Dear Dr. Noble:

Congratulations, your manuscript titled "Preventing evolutionary rescue in cancer using two-strike therapy" is accepted for publication in GENETICS! Many thanks for submitting your research to the journal and for this nice contribution.
One reviewer suggests to double check one equation in footnote 10 in the appendix. You can view this comment at the bottom of this email, and address this point when providing a finalized version of your paper.

To Proceed to Publication:
1. Format your article according to GENETICS style: https://academic.oup.com/genetics/pages/author-guidelines

2. Ensure that you comply with data and community resource citation guidelines:
https://academic.oup.com/genetics/pages/author-guidelines#section-5-9-2

3. Upload your final files at https://genetics.msubmit.net

4. Add oupsupport@scipris.com and genetics.oup@novatechset.com (or the domains @scipris.com and @novatechset.com) to your email program's "safe senders" list. You will be contacted by both at various points during the production process.

Notes:
- Your currently-accepted manuscript (unedited, as submitted, reviewed, and accepted) will be published at GENETICS and deposited into PubMed as an Advance Access article. Notify sourcefiles@thegsajournals.org before signing your license if you do not wish to publish your article via Advance Access.

- We invite you to submit an original color figure related to your paper for consideration as cover art. Please email your submission to the editorial office or upload it with your final files. You can submit a small-sized image for evaluation, and if selected, the final image must be a TIFF file 2513px wide by 3263px high (8.375 by 10.875 inches; resolution of 600ppi). Please avoid graphs and small type.

- After files are sent to Oxford University Press we use SciPris to manage article licensing and payment. If you do not have a SciPris account, you will receive an email from no-reply@scipris.com to sign up to use Oxford University Press' author portal. After logging in, follow the online instructions to sign your license and arrange any payment due.

If you have any questions or encounter any problems while uploading your accepted manuscript files, please email the editorial office at sourcefiles@thegsajournals.org.

Sincerely,

Thomas Lenormand
Associate Editor
GENETICS

Approved by:
Nicholas Barton
Senior Editor
GENETICS

Review comments (if applicable):

Reviewer #1 :

I think this is a very nice paper.

Reviewer #3 :

I am satisfied with the authors' responses to my previous comments. Thanks in particular for the explanation regarding the calculation of rescue from R1,2 mutants.

I think there is a minor error remaining in one equation in Footnote 10; please double check: In the equation starting 1/mu2 [RM(t_opt + Delta) ...], in the middle equality, shouldn't there be coefficients alpha_S in front of S(t_opt) and alpha_1 in front of R1(t_opt)?
